# Trends in predominant causes of death in individuals with and without diabetes in England from 2001 to 2018: an epidemiological analysis of linked primary care records

**DOI:** 10.1016/S2213-8587(20)30431-9

**Published:** 2021-03

**Authors:** Jonathan Pearson-Stuttard, James Bennett, Yiling J Cheng, Eszter P Vamos, Amanda J Cross, Majid Ezzati, Edward W Gregg

**Affiliations:** a Department of Epidemiology and Biostatistics, School of Public Health, Imperial College London, London, UK; b MRC Centre for Environment and Health, Imperial College London, London, UK; c Department of Primary Care and Public Health, Imperial College London, London, UK; d Cancer Screening and Prevention Research Group, Department of Surgery and Cancer, Imperial College London, London, UK; e Division of Diabetes Translation, US Centers for Disease Control and Prevention, Atlanta, GA, USA

## Abstract

**Background:**

The prevalence of diabetes has increased in the UK and other high-income countries alongside a substantial decline in cardiovascular mortality. Yet data are scarce on how these trends have changed the causes of death in people with diabetes who have traditionally died primarily of vascular causes. We estimated how all-cause mortality and cause-specific mortality in people with diabetes have changed over time, how the composition of the mortality burden has changed, and how this composition compared with that of the non-diabetes population.

**Methods:**

In this epidemiological analysis of primary care records, we identified 313 907 individuals with diabetes in the Clinical Practice Research Datalink, a well described primary care database, between 2001 to 2018, and linked these data to UK Office for National Statistics mortality data. We assembled serial cross sections with longitudinal follow-up to generate a mixed prevalence and incidence study population of patients with diabetes. We used discretised Poisson regression models to estimate annual death rates for deaths from all causes and 12 specific causes for men and women with diabetes. We also identified age-matched and sex matched (1:1) individuals without diabetes from the same dataset and estimated mortality rates in this group.

**Findings:**

Between Jan 1, 2001, and Oct 31, 2018, total mortality declined by 32% in men and 31% in women with diagnosed diabetes. Death rates declined from 40·7 deaths per 1000 person-years to 27·8 deaths per 1000 person-years in men and from 42·7 deaths per 1000 person-years to 29·5 deaths per 1000 person-years in women with diagnosed diabetes. We found similar declines in individuals without diabetes, hence the gap in mortality between those with and without diabetes was maintained over the study period. Cause-specific death rates declined in ten of the 12 cause groups, with exceptions in dementia and liver disease, which increased in both populations. The large decline in vascular disease death rates led to a transition from vascular causes to cancers as the leading contributor to death rates in individuals with diagnosed diabetes and to the gap in death rates between those with and without diabetes.

**Interpretation:**

The decline in vascular death rates has been accompanied by a diversification of causes in individuals with diagnosed diabetes and a transition from vascular diseases to cancers as the leading contributor to diabetes-related death. Clinical and preventative approaches must reflect this trend to reduce the excess mortality risk in individuals with diabetes.

**Funding:**

Wellcome Trust.

## Introduction

The number of adults living with diabetes has increased globally from 108 million in 1980, to 422 million in 2014,^1^ due to a rise in prevalence, population growth, and ageing. Diabetes represents a substantial health challenge for individuals, the health system, and wider economy.

Diabetes has a well-established association with increased risk of vascular conditions, such as ischaemic heart disease,^2^ stroke,^2^, ^3^ renal,^4^ and neuropathic complications,^5^ that has led to widely implemented specific guidelines for secondary prevention for patients with these typical diabetes complications.^6^ Death rates from these causes, particularly cardiovascular disease, have declined by as much as 50% in the general UK population and other high-income countries over the past four decades.^7^, ^8^, ^9^, ^10^ Some evidence suggests that these improvements are accompanied by a diversification or even a worsening of other outcomes,^7^, ^11^ including infections, liver disease,^12^ dementia,^13^, ^14^ and certain cancers.^15^ However, few studies, and none in the UK, have examined trends in specific causes of death, leaving the contemporary burden of diabetes-related mortality poorly characterised. Understanding these trends could have important implications for public health, preventative, and clinical measures.

We estimated how all-cause and cause-specific mortality rates in people with diabetes have changed over time, how the composition of the mortality burden has changed, and how this composition compared with that of the non-diabetes population

**Research in context**

**Evidence before this study**

We searched PubMed for reports of population-based analyses of trends in causes of death in individuals with diabetes from Jan 1, 1990, to June 22, 2020, using the terms “mortality trends”, “diabetes mellitus”, “cause-specific mortality”, and “diabetes mellitus”, without language restrictions. Research outside of the UK suggests that all-cause death rates have declined in individuals with diabetes, leading to a diversification of causes of death; however, the composition of this diversification has varied across countries.

**Added value of this study**

Large declines in vascular disease death rates have been accompanied by cancer becoming the largest contributor to death rates and the diabetes-related gap in all-cause death rates. In line with previous work, we found that all-cause death rates have declined in individuals with and without diabetes from 2001 to 2018, whereas death rates declined in ten of 12 causes in those with diabetes. Death rates have increased in patients with liver disease and dementia.

**Implications of all the available evidence**

The decline in vascular disease death rates has led to a diversification of causes of death in individuals with diabetes and a transition from vascular diseases to cancers as the leading contributor to diabetes-related death. Preventative approaches in individuals with diabetes should be redressed to reflect the diversification of cause of death, with a particular focus on cancers.

## Methods

### Study design and participants

In this epidemiological analysis of linked primary care data, we estimated death rates and the cause composition of deaths in adults in England with and without diabetes from 2001 to 2018 using the Clinical Practice Research Datalink (CPRD) GOLD. CPRD is a primary care database of over 45 million patients^16^ that was initiated in 1994. CPRD contains pseudo-anonymised patient data from 674 general practices in England covering approximately 7% of the population. The database is broadly representative of the population in terms of age, sex, and ethnicity.^16^ Primary care practices who participate in CPRD follow an agreed protocol for the collection of demographic, clinical, laboratory, and prescription data, and regularly submit anonymised records to the database. The data from each practice, and of each contributing patient, are assessed by CPRD to check the quality and completeness for inclusion in research studies.

### Analytic dataset and sample selection

We extracted records from Jan 1, 2001, Oct 31, 2018, of patients with diabetes. Within CPRD, we assembled serial cross sections with longitudinal follow-up to generate a mixed prevalence and incidence study population of patients with diabetes. We used 2001 as the earliest date because mortality coding switched from International Statistical Classification of Diseases (ICD)-9 to ICD-10 in 2001. We identified 313 907 individuals with diabetes during the study period. The annual number of people living with diabetes increased from 68 319 in 2001, to 219 547 in 2018 (appendix pp 1–3).

We identified individuals with diabetes using diagnostic (C10) and management (66A) codes from the Read and Oxford Medical Information System for prescription data for diabetes^17^ and glucose-lowering therapy. We included individuals with either type 1 or type 2 diabetes. We included those with prevalent diabetes in the study from the date of diabetes diagnosis if (1) they were aged 18 years or older (or joined the cohort when they became 18 years old during study follow-up), (2) the patient record was marked as acceptable by CPRD for research, and (3) the participating primary care practice was deemed to be contributing up-to-standard data by CPRD. The CPRD registered study protocol number was 19_118.

### Diabetes diagnosis and follow-up

We included patients with diabetes in the study population from the earliest of either (1) event date of a clinically recorded (Read code) diagnosis of diabetes or diabetes-related clinical encounter, or (2) event date of prescription of a glucose-lowering therapy, providing that the patient record had at least two such prescriptions. We included patients without diabetes from the date that they entered the CPRD population if the patient record was marked as acceptable by CPRD for research, and the participating primary care practice was deemed to be contributing up-to-standard data by CPRD. The individuals remained in the study population until either the date of death, end of the study period (Oct 31, 2018), or they were transferred out of CPRD. Therefore, the diabetes population (patients diagnosed with diabetes) comprised individuals with varying diabetes durations, including those who had diabetes at the beginning of the study period and those who were diagnosed in a given year during the study period. Year (calendar period), age, diabetes duration, and death status were time-dependent variables and diabetes status (yes or no) and sex (male or female) were non time-dependent variables.

### Matched non-diabetes participants

We initially, by age (year of birth) and sex, participants within the CPRD dataset without diabetes, who were present in the dataset from the same start year (year joining the cohort) or earlier, and were linked to Office for National Statistics (ONS) mortality data. This method provided matches for less than 70% of the diabetes population. Therefore, for the analysis, we matched for decade of birth rather than year of birth, resulting in 313 907 matched non-diabetes individuals. 178 702 non-diabetes individuals were in the study cohort in 2001, increasing to 232 811 in 2018 (appendix pp 1–3). Individuals with a diabetes diagnosis were eligible for inclusion in the non-diabetes population until their diagnosis date, at which point they were censored.

### Mortality cause linkage and groupings

We extracted records of individuals with and without diabetes linked to mortality data from national death registries from the ONS, which uses a coding system equivalent to that of the ICD-10. In addition to all-cause mortality, we structured 12 clusters of causes of death, with each grouping being comprehensive and mutually exclusive (appendix pp 4–6). These clusters were ischaemic heart disease, stroke, other circulatory disease, renal, liver, respiratory, diabetes, injuries, dementia, diabetes-associated cancers, all-other cancers, and other causes. The diabetes-associated cancers were those that have been identified as diabetes likely being a causal risk factor for the given cancer,^15^ and included colorectal, pancreatic, liver, gallbladder, breast, and endometrial cancers.

### Statistical analyses

We used a discrete Poisson regression model^18^ to estimate annual death rates and compare rates between the diabetes and non-diabetes population. We split the study period into discrete 1-year periods.

Each individual was allocated an exposure length for each discrete year ranging between 0 and 1 (with the exception of the final study year, 2018, for which maximum exposure was 10 months). For example, an individual who died on Jan 31 would be given an exposure length of 31/365 for that year and 0 in subsequent years. The age, diabetes duration, and death status of each individual were updated for each discrete year period.

We used the marginal rate from the Poisson regression to estimate adjusted all-cause and cause-specific death rates. For the sex-specific analysis we used separate (stratified) models for men and women. This model included interaction terms between diabetes status and discrete year and age. The reported rates for each year in the study period correspond to a population that has the same age distribution as the entire sample, those with and without diabetes, over the entire analysis period. For the sex-specific analysis, we used separate models for men and women. Similarly, for the age-specific analysis, we used three separate models for age groups (<55 years, 55–75 years, >75 years).

For the aggregate (ie, non sex-specific) analysis, we included in the Poisson model interaction terms between diabetes status and discrete year, age and sex, which allowed analyses of whether differences in the rate of change (increase or decline) in death rates existed between the diabetes and non-diabetes population. We further included an interaction term between sex and discrete year.

Using calendar years as a continuous variable, we calculated the absolute change (average difference), expressed as a rate per 10 years, for each cause (table 1). All data management and assembly of datasets were done using R Studio (version 1.2.5042), and the statistical analysis were done using Stata (version 15.1).

### Role of the funding source

The funder of the study had no role in study design, data collection, data analysis, data interpretation, or writing of the report. JP-S and EV had access to and verified the extracted raw data from CPRD. JP-S and YC verified all study analyses. JP-S had final responsibility for the decision to submit for publication.

## Results

**Figure 1:**
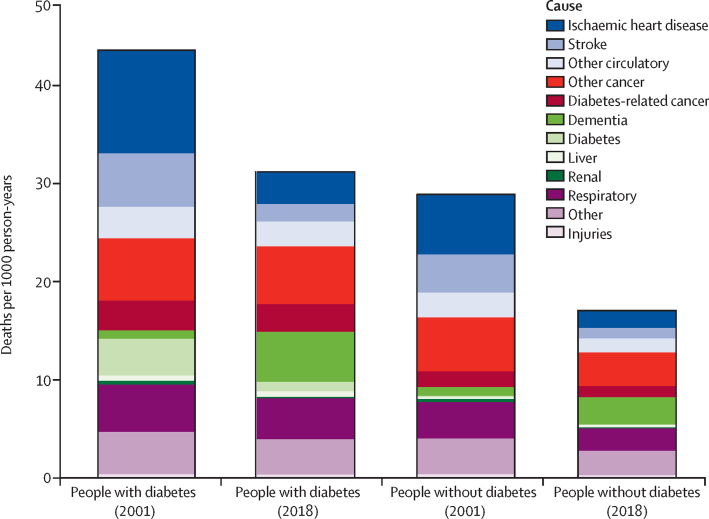
Death rates by cause in those with and without diabetes in 2001 and 2018

From Jan 1, 2001 to Oct 31, 2018, all-cause death rates declined in individuals with and without diabetes (figure 1). Specifically, all-cause death rates declined by 32% (from 40·7 deaths per 1000 person-years to 27·8 per 1000 person-years) in men and 31% (42·7 deaths per 1000 person-years to 29·5 per 1000 person-years) in women with diabetes and by 41% (28·4 deaths per 1000 person-years to 16·7 per 1000 person-years) in men and 33% (28·1 deaths per 1000 person-years to 18·7 per 1000 person-years) in women without diabetes (table; appendix p 17). Thus, the absolute gap in death rates between individuals with and without diabetes was maintained throughout this period, with a difference of 12·3 deaths per 1000 person-years in 2001, and a difference of 11·1 deaths per 1000 person-years in 2018, in men, and a difference of 14·5 deaths per 1000 person-years in 2001, and a difference of 10·8 deaths per 1000 person-years in 2018, in women. We found a consistent social gradient throughout the study period, with mortality rates being higher in the most deprived compared with the most affluent quintiles across both the diabetes and non-diabetes populations. The mortality gap between those quintiles declined over the 18-year period (appendix p 7).

**Table:** Adjusted all-cause and cause-specific mortality in 2001 and 2018 per 1000 person years and average 10-year absolute change in death rate in people with and without diabetes, by sex

		**People with diabetes**			**People without diabetes**		
		2001: rate per 1000 person years (95% CI)	2018: rate per 1000 person years (95% CI)	Average 10-year absolute change (95% CI)	2001: rate per 1000 person years (95% CI)	2018: rate per 1000 person years (95% CI)	Average 10-year absolute change (95% CI)
**Men**							
All-cause		40·7 (40·0 to 41·5)	27·8 (27·3 to 28·3)	−9·0 (−8·8 to −9·3)	28·4 (27·7 to 29·0)	16·7 (16·3 to 17·0)	−7·2 (−7·1 to −7·4)
Vascular causes		18·5 (18·0 to 19·1)	7·5 (7·3 to 7·7)	−6·0 (−5·9 to −6·1)	12·2 (11·7 to 12·7)	4·3 (4·2 to 4·5)	−4·2 (−4·1 to −4·3)
	Ischaemic heart disease	11·2 (10·7 to 11·6)	4·0 (3·8 to 4·1)	−4·3 (−4·2 to −4·4)	6·2 (6·0 to 7·0)	2·2 (2·1 to 2·3)	−2·8 (−2·7 to −2·8)
	Stroke	4·7 (4·4 to 5·0)	1·5 (1·4 to 1·6)	−1·9 (−1·9 to −2·0)	3·3 (3·0 to 3·5.3)	0·9 (0·8 to 1·0)	−1·4 (−1·3 to −1·4)
	Other circulatory	2·9 (2·7 to 3·1)	2·2 (2·1 to 2·4)	−0·6 (−0·5 to −0·6)	2·3 (2·1 to 2·5)	1·2 (1·1 to 1·3)	−0·8 (−0·7 to −0·8)
Cancers		10·5 (10·1 to 10·9)	9·3 (9·0 to 9·7)	−0·7 (−0·5 to −0·8)	8·1 (7·8 to 8·4)	4·8 (4·6 to 5·0)	−1·9 (−1·8 to −2·0)
	Other cancer	8·5 (8·1 to 8·8)	7·7 (7·4 to 8·0)	−0·5 (−0·3 to −0·6)	7·2 (6·9 to 7·5)	4·4 (4·3 to 4·6)	−1·6 (−1·5 to −1·7)
	Diabetes-related cancer	2·8 (2·6 to 3·0)	2·5 (2·4 to 2·7)	−0·2 (−0·1 to −0·2)	1·3 (1·2 to 1·4)	0·9 (0·8 to 1·0)	−0·2 (−0·2 to −0·3)
Renal		0·4 (0·3 to 0·5)	0·2 (0·1 to 0·2)	−0·2 (−0·1 to −0·2)	0·4 (0·3 to 0·5)	0·1 (0·1 to 0·2)	−0·1 (−0·1 to −0·2)
Liver		0·6 (0·5 to 0·7)	0·7 (0·6 to 0·8)	0·1 (0·0 to 0·1)	0·3 (0·2 to 0·3)	0·3 (0·2 to 0·3)	0·0 (−0·0 to 0·0)
Respiratory		4·7 (4·4 to 4·9)	4·0 (3·8 to 4·2)	−0·3 (−0·3 to −0·4)	3·5 (3·3 to 3·8)	2·2 (2·1 to 2·4)	−0·9 (−0·8 to −0·9)
Diabetes		3·1 (2·7 to 3·3)	0·8 (0·8 to 0·9)	−1·2 (−1·2 to −1·3)	0·1 (0·1 to 0·1)	0·1 (0·1 to 0·1)	−0·0 (−0·0 to −0·0)
Dementia		0·6 (0·5 to 0·7)	3·8 (3·5 to 4·1)	1·8 (1·7 to 1·8)	0·7 (0·6 to 0·7)	1·8 (1·7 to 1·9)	0·8 (0·8 to 0·9)
Injuries		0·4 (0·3 to 0·5)	0·4 (0·3 to 0·5)	−0·0 (−0·0 to −0·1)	0·4 (0·3 to 0·6)	0·4 (0·3 to 0·4)	−0·1 (−0·0 to −0·1)
Other		3·4 (2·2 to 3·6)	3·2 (3·1 to 3·4)	−0·2 (−0·2 to −0·3)	3·1 (2·9 to 3·3)	2·3 (2·1 to 2·4)	−0·5 to (−0·5 to −0·6)
**Women**							
All-cause		42·7 (41·8 to 43·5)	29·5 (28·9 to 30·0)	−7·2 (−7·1 to −7·4)	28·1 (27·5 to 28·9)	18·7 (18·3 to 19·1)	−5·1 (−5·0 to −5·3)
Vascular causes		19·3 (18·7 to 20·0)	7·1 (6·9 to 7·4)	−6·6 (−6·4 to −6·7)	13·0 (12·5 to 13·6)	4·4 (4·2 to 4·6)	−4·5 (−4·4 to −4·6)
	Ischaemic heart disease	10·0 (9·4 to 10·3)	2·5 (2·4 to 2·6)	−3·2 (−3·2 to −3·3)	5·7 (5·3 to 6·1)	1·4 (1·3 to 1·5)	−1·8 (−1·7 to −1·8)
	Stroke	6·4 (6·0 to 6·8)	2·1 (2·0 to 2·3)	−1·9 (−1·9 to −2·0)	4·7 (4·3 to 5·0)	1·3 (1·2 to 1·4)	−1·5 (−1·4 to −1·5)
	Other circulatory	3·5 (3·2 to 3·7)	2·8 (2·6 to 3·0)	−0·3 (−0·2 to −0·3)	2·7 (2·5 to 2·9)	1·6 (1·5 to 1·7)	−0·5 (−0·5 to −0·6)
Cancers		8·3 (7·9 to 8·7)	8·1 (7·7 to 8·4)	−0·1 (−0·0 to −0·3)	5·9 (5·7 to 6·2)	4·2 (4·1 to 4·4)	−1·0 (−0·9 to −1·1)
	Other cancer	7·2 (6·9 to 7·5)	4·2 (4·1 to 4·4)	−0·1 (−0·1 to −0·2)	3·9 (3·7 to 4·1)	2·5 (2·4 to 2·7)	−0·8 (−0·8 to −0·9)
	Diabetes-related cancer	4·5 (4·2 to 4·7)	3·1 (2·9 to 3·3)	−0·0 (−0·1 to 0·0)	1·8 (1·7 to 1·9)	1·4 (1·3 to 1·4)	−0·3 (−0·2 to −0·3)
Renal		0·5 (0·4 to 0·6)	0·2 (0·2 to 0·2)	−0·1 (−0·1 to −0·2)	0·3 (0·2 to 0·4)	0·1 (0·1 to 0·2)	−0·1 (−0·1 to −0·1)
Liver		0·4 (0·3 to 0·4)	0·5 (0·4 to 0·6)	0·1 (0·1 to 0·1)	0·1 (0·1 to 0·2)	0·2 (0·1 to 0·2)	0·0 (0·0 to 0·0)
Respiratory		5·1 (4·8 to 5·4)	0·4 (4·2 to 4·7)	−0·4 (−0·4 to −0·5)	4·0 (3·7 to 4·3)	2·4 (2·2 to 2·5)	−0·8 (−0·7 to −0·8)
Diabetes		4·5 (4·2 to 4·8)	1·1 (1·0 to 1·2)	−1·5 (−1·5 to −1·6)	0·1 (0·1 to 0·2)	0·1 (0·0 to 0·1)	−0·0 (−0·0 to −0·0)
Dementia		1·2 (1·1 to 1·3)	6·6 (6·2 to 7·0)	2·1 (2·1 to 2·2)	1·2 (1·1 to 1·3)	3·9 (3·7 to 4·1)	1·2 (1·1 to 1·2)
Injuries		0·4 (0·4 to 0·5)	0·3 (0·3 to 0·4)	−0·0 (−0·0 to −0·1)	0·4 (0·3 to 0·5)	0·2 (0·2 to 0·3)	−0·1 (−0·1 to −0·1)
Other		5·3 (5·0 to 5·6)	4·0 (3·8 to 4·2)	−0·6 to (−0·5 to −0·6)	4·2 (4·0 to 4·5)	2·8 (2·6 to 2·9)	−0·8 (−0·7 to −0·8)

Rates in 2001 and 2018 correspond to a population that has the same age distribution as the entire sample—ie, those with and without diabetes—over the entire analysis period. Diabetes-associated cancers are colorectal, pancreatic, liver, gallbladder, breast, and endometrial cancers.

**Figure 2:**
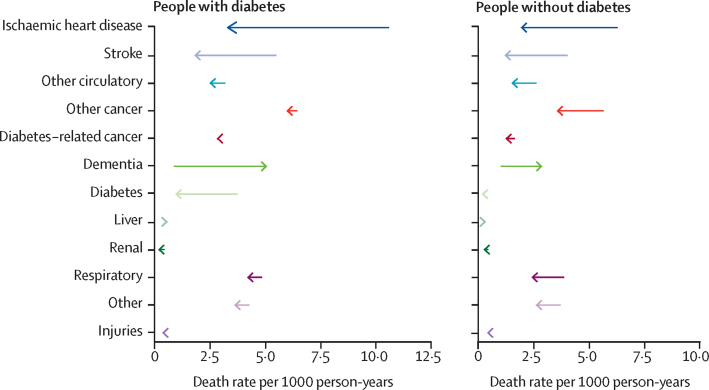
Absolute change in cause-specific death rates from 2001 to 2018 in those with and without diabetes

Among the 12 pre-specified causes, the death rate per year for every cause was higher in the diabetes population compared with in the non-diabetes population at both the beginning and end of the study period, with the exception of dementia (both sexes) and injuries (men only) in 2001 (table; figure 2; appendix p 18). Death rates declined in ten of our 12 cause-specific groups in both populations (table; figure 2).

**Figure 3:**
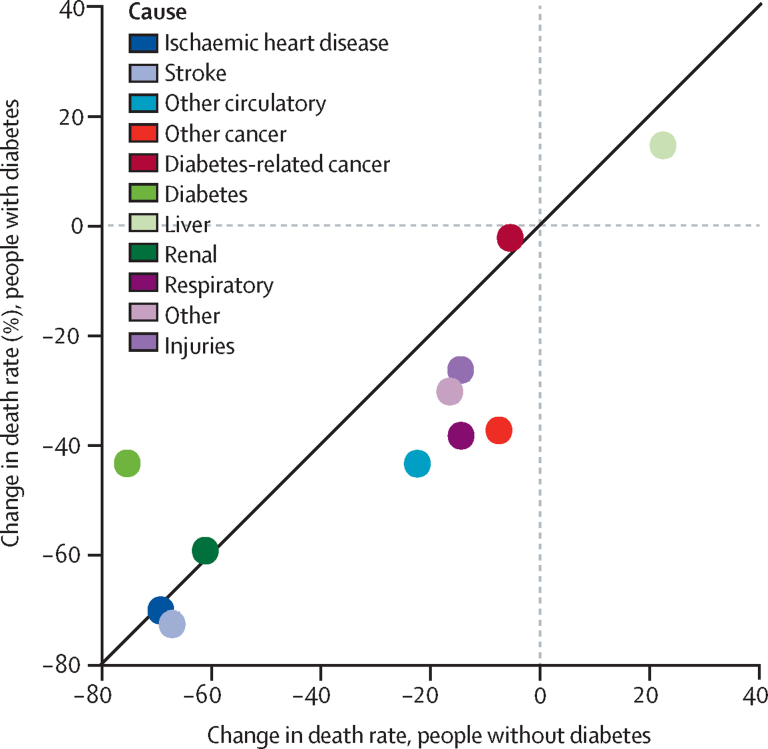
Relative change in cause-specific death rate from 2001 to 2018 in people with diabetes compared with those without diabetes

Among individuals with diabetes, the largest absolute decline in specific causes of death was seen in ischaemic heart disease (7·2 deaths per 1000 person-years), stroke (3·6 deaths per 1000 person-years) and diabetes (2·8 deaths per 1000 person-years; figure 2), with similar improvements in men and women (appendix p 18). Similar patterns were observed in the non-diabetes population (table). By contrast with these improvements in vascular-related deaths, rates of death due to dementia and liver disease increased over the study period in both groups, although by a larger amount in the diabetes population compared with in the non-diabetes population. Death rates increased from 0·9 deaths per 1000 person-years to 5·1 deaths per 1000 person-years for dementia and from 0·5 deaths per 1000 person-years to 0·6 deaths per 1000 person-years for liver disease in individuals with diabetes (figure 2). Cancer deaths decreased in both populations over the study period but with much larger declines in the non-diabetes population (declines of 2·1 deaths per 1000 person-years from all other cancers and 0·4 deaths per 1000 person-years from diabetes-related cancers, compared with declines of just 0·5 deaths per 1000 person-years and 0·2 deaths per 1000 person-years in the diabetes population; figure 2). Generally, relative declines in death rates were larger in the non-diabetes population with the exception of death due to diabetes and we found similar trends in men and women (figure 3; appendix p 19).

In 2001, the gap in cause-specific death rate between individuals (men and women combined) with and without diabetes was largest in ischaemic heart disease (difference of 4·4 deaths per 1000 person-years), diabetes (3·6 deaths per 1000 person-years), stroke (1·6 deaths per 1000 person-years), and diabetes-related cancers (1·5 deaths per 1000 person-years; figure 1). This diabetes-associated gap in death rates between diabetes and non-diabetes populations declined substantially from 2001 to 2018 for ischaemic heart disease and stroke but increased in several causes including dementia, other cancers, diabetes-associated cancers, and respiratory diseases. As a result, by 2018, cancers had replaced vascular diseases as the greatest cause of excess death associated with diabetes (table). Specifically, the greatest diabetes-related gap in death rates between those with and without diabetes in 2018 was seen in other cancers (difference of 2·5 deaths per 1000 person-years), dementia (2·3 deaths per 1000 person-years), diabetes-associated cancers (1·7 deaths per 1000 person-years), and respiratory deaths (1·9 deaths per 1000 person-years), with much reduced gaps in ischaemic heart disease (1·5 deaths per 1000 person-years) and stroke (0·7 deaths per 1000 person-years; figure 2). This finding was driven by a combination of a major reduction in vascular disease mortality in the diabetes population, with declines greater than in the non-diabetes population, alongside much more modest improvements in cancer deaths that were more modest than in the non-diabetes population (figure 2). The 10-year absolute difference in mortality rate was significantly greater in those with diabetes for all-cause mortality and several specific causes, namely ischaemic heart disease, stroke, diabetes, and dementia across both sexes, and renal and liver diseases in women (table). By contrast, the 10-year decline in mortality rate was substantially greater in the non-diabetes population for respiratory diseases, all-other cancers, other circulatory diseases, and other causes, and diabetes-associated cancers in women only.

**Figure 4:**
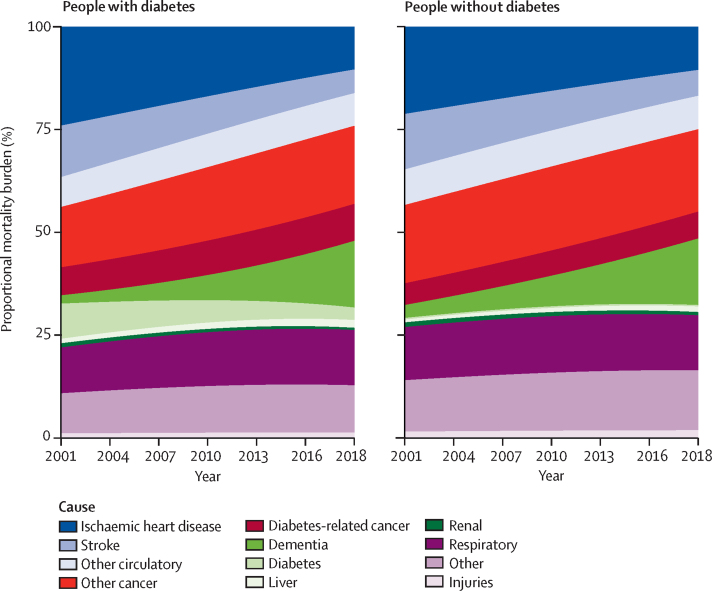
Proportional contribution to mortality burden of causes of death in people with and without diabetes

The proportion of deaths due to vascular diseases declined from 44% in 2001 to 24% in 2018 in individuals with diabetes and from 43% to 25% in those without diabetes (figure 4). Conversely, the proportion of deaths due to cancer increased from 22% in 2001 to 28% in 2018 in individuals with diabetes and from 24% in 2001 to 27% in 2018 in those without diabetes (figure 4). Thus, cancers accounted for a larger proportion of deaths than did vascular diseases by 2018 in individuals with and without diabetes. The similar proportion of deaths attributable to cancer, however, masks the differences in cancer composition. Diabetes-related cancers were attributable for almost 50% more mortality burden in those with diabetes compared to in those without diabetes (9·0% vs 6·5%; appendix pp 8–9). Dementia accounted for an increasing proportion of deaths throughout the period, in 2018 accounting for 16% of all deaths in individuals with and without diabetes compared with just 2% in those with diabetes and 3% in those without diabetes in 2001. The cause-specific contributions to the mortality burden varied by age but were similar between those with and without diabetes across sex and age groups (appendix pp 20, 23).

## Discussion

We found that death rates declined considerably in individuals with and without diabetes from 2001 to 2018, accompanying a substantial reduction in the rate of vascular disease mortality. The diabetes-associated gap in death rates, however, increased in several diseases including dementia, respiratory disease, and other cancers. This increase resulted in a transition from vascular disease causes to cancer as the leading contributor to the gap in death rates between individuals with and without diabetes and a general diversification of causes of death away from vascular causes towards dementia, cancer, and liver disease.

Our findings, which used more comprehensive and detailed estimates compared with other studies, are generally consistent with and build upon previous findings on trends in all-cause and cause-specific death rates. The declines in all-cause mortality in individuals with and without diabetes are greater than those found in national estimates;^18^ however, when standardised, the declines in our study are more similar (appendix p 16). Death rates for vascular disease have declined over the past four decades in individuals with^7^, ^11^, ^19^ and without diabetes,^7^, ^8^ which is generally attributed to improvements in treatment pathways, risk factor management, and lifestyle behaviours.^10^, ^20^ Our findings are also consistent with previous observations that the gap in absolute death rates between individuals with and without diabetes has generally declined.^21^ Our findings that the share of the mortality burden due to cancers has increased across all age groups in individuals with diabetes differs from previous work in the US population with diabetes,^7^ but is consistent with findings in the Australian population with diabetes.^11^ The identification of cancer as the leading contributor to death in individuals with diabetes and the leading contributor to the gap in death rates between those with and without diabetes is different to the US and Australian findings and is novel. Our findings of worsening death rates for dementia in individuals without as well as those with diabetes is consistent with previous work analysing the general population in England^22^ and those with diabetes in the USA.^7^ We found death rates for patients with liver disease increased in both populations whereas the US study found a small decline in death rates from liver disease in those with diabetes and no change in those without diabetes.

Several of our findings were expected while some were not. First, substantial and comparatively larger declines in cardiovascular disease death rates among individuals with diabetes resulted in a halving of the diabetes gap in rates of deaths due to vascular disease over this period. This success probably reflects the improvements in behavioural risk factors such as smoking, cholesterol, and salt consumption, additional targeting of prevention measures in individuals with diabetes according to risk,^6^ and improved survival in those with the disease. The introduction of the Quality Outcomes Framework in 2004, which incentivised primary care physicians to identify, monitor, and treat risk factors in individuals with chronic diseases such as diabetes, might have contributed to lowering vascular disease mortality rates and identified those with diabetes at an early stage in their disease resulting in a lead-time bias; however, previous analyses have had mixed findings, suggesting that this method identified more patients with diabetes, but a greater proportion of those with HbA_1C_ below 7·5%, compared with those with HbA_1C_ above 7·5%.^23^

Second, unlike other causes of death, death rates for dementia and liver disease increased over the study period. Dementia became the leading cause of death in the general population in England in 2018,^24^ in part because of the success in reducing deaths from other leading diseases such as ischaemic heart disease and stroke, which tend to occur earlier in life but share risk factors with dementia, as well as increasing age of death throughout the study period in individuals with and without diabetes—individuals with diabetes have a 1·5–2·5-times increased risk of dementia compared with those without diabetes.^25^ The increase in liver disease death rates could be explained by an increase in common risk factors, such as obesity^26^ and persistently high alcohol consumption.^27^ The association could also be bi-directional; fatty liver disease increases the risk of diabetes, and diabetes itself is associated with a range of non-cancer liver conditions including cirrhosis and hepatitis.^12^

Third, because of improvements in cancer death rates lagging behind the vast improvements in vascular disease death rates, cancer became the leading contributor to the gap in death rates between individuals with and without diabetes and replaced vascular disease the leading cause of death in those with and without diabetes. Decreases in cancer death rates were modest across all groups, more modest in the diabetes population than in the non-diabetes population, and death rates increased in older adults (>75 years). The UK continues to lag behind many European countries in cancer survival rates, with earlier diagnosis and more equitable care pathways cited as important areas to address. Also, clinical and secondary prevention guidelines for individuals with diabetes must be updated to reflect the increased risk of several common cancers^15^ and as the leading killer in this patient group.

Finally, death rates in women with diabetes were higher than in men. This finding is partially explained by the older age of the population of women (with and without diabetes) and the fact that separate models were used for sex-specific analyses given the evidence of cause composition and that causal^28^ and non-causal effects differ by sex. In women with diabetes, we found larger absolute declines in rates of death from diabetes compared with in men, but found the reverse for ischaemic heart disease and other cancers, for which declines were larger in men than women.

Clinical and preventative efforts should broaden to encompass the diversity of mortality risk in individuals with diabetes. Traditionally, such efforts have focused on controlling vascular risk factors such as hypertension and cholesterol.^6^ Our findings suggest that this focus has been effective in reducing vascular disease mortality and excess risk in individuals with diabetes. This approach of targeting clinical and preventive approaches towards excess risk should be expanded to take a much broader approach accounting for the wider causes of death, especially for patients with specific cancers, liver diseases, and dementia. Some evidence, for example, suggests that specific therapeutics could delay the onset or worsening of cognitive impairments in individuals with diabetes.^29^ This finding will have implications for care pathways coalescing around patient's health needs rather than organ-based pathology.^30^, ^31^

A strength of our study is the use of a large representative dataset enabling stratification by age, sex, and deaths into 12 cause groups. Further, although longitudinal primary care, administrative, and real-world databases can be problematic through coding variation and missingness, CPRD has been used and detailed extensively for epidemiological research^16^ and is representative of the English population,^16^ enabling generalisability of our findings. Still, these types of datasets do have limitations as real-world, non-trial conditions of capturing the data. Miscoding, misdiagnosis, and misclassification in administrative datasets such as CPRD have been extensively assessed and documented in previous work,^32^ and we highlight trends in demographic and risk factor data and missingness (appendix p 1). For these reasons, we did not solely rely upon diagnostic codes for diabetes and instead used a pragmatic approach including algorithms that considered the combination of diagnostic codes, administrative codes, and medications; and overcame conflicting recordings; and used a previously used approach for identifying individuals with diabetes within CPRD.^33^ However, this method did not capture individuals with undiagnosed diabetes, which is estimated to account for around 20% of those with diabetes in the UK today^34^ and 5–10% of the general population in England,^35^ thus our findings most accurately pertain to the individuals with diagnosed diabetes. For these reasons, our method of identifying diabetes duration from first code recorded in patient records is imperfect. Further, we did not stratify by diabetes type (1 or 2) because we did not have data to accurately distinguish type 1 from type 2 diabetes consistently over the entire period of study and more important, the number of individuals with type 1 diabetes would be insufficient to reliably examine trends in cause-specific mortality over the 18-year period.

Our Poisson model accounted for several covariates that are likely to influence mortality risk, including age, sex, year, and, unlike many previous analyses, duration of diabetes. Administrative datasets, however, such as CPRD have higher rates of missing data regarding covariates than clinical trials data do. For example, in the early years of our study (2001–05), approximately 60% of individuals in CPRD have blood pressure or smoking status recorded for the past 3 years and corresponding figures for BMI are even lower at 30%.^16^ Covariate measurements are even less present annually as would be required for this analysis, hence we were unable to include cardiometabolic risk factors into our Poisson model, nor were we able to explore whether associations were likely to be causal, and underlying mechanisms.

Death data were obtained through linkage to ONS death statistics and thus deaths that occurred outside of the UK (emigration or during holidays) would not have been captured; however these deaths were likely to represent a small portion of all deaths. We also did analyses by age (appendix pp 22–24) and in more detailed younger adult stratifications (appendix p 24) given the finding that young-adult onset of type 2 diabetes has increased^36^ and found generally similar mortality trends across age groups with the exception of worsening of cancer death rates in individuals younger than 45 years.

The large declines in all-cause death rates and vascular disease death rates have led to cancer being the leading contributor to the gap in death rates between those with and without diabetes. Further longevity gains and reducing the gap between individuals with and without diabetes will require a shift in clinical and preventative approaches to encompass the wider set of disease risks.

## Data sharing

Study data will not be made available as CPRD data requires an application and permissions to access the patient level data.
