# Supplementary material for: Trends in predominant causes of death in individuals with and without diabetes in England from 2001 to 2018: an epidemiological analysis of linked primary care records
Source: Lancet Diabetes Endocrinol. 2021 Mar;9(3):165–73. doi: 10.1016/S2213-8587(20)30431-9 (PMC7886654; doi:10.1016/S2213-8587(20)30431-9)
Supplement: Supplementary appendix [file mmc1.pdf]

THE LANCET
Diabetes & Endocrinology

Supplementary appendix

This appendix formed part of the original submission and has been peer reviewed. We post it as supplied by the authors.

Supplement to: Pearson-Stuttard J, Bennett, J Cheng Y J, et al. Trends in predominant causes of death in individuals with and without diabetes in England from 2001 to 2018: an epidemiological analysis of linked primary care records. *Lancet Diabetes Endocrinol* 2021; published online Feb 4. https://doi.org/10.1016/S2213-8587(20)30431-9.

Supplemental Table 1. Characteristics of English adults in the Clinical Research Practice Datalink with and without diabetes 2001-2018 in A) Men, B) Women. *Note: missingness of body mass index measurements annually is variable over time and across diabetes/non-diabetes population. Median (interquartile range in parentheses) for continuous variables*

A)

	Diabetes				Non-diabetes			
**Year**	**2001**	**2007**	**2013**	**2018**	**2001**	**2007**	**2013**	**2018**
**Prevalent cohort, n**	36776	78391	115251	117345	93471	107606	117438	114464
**Cohort age (years)**	64 (54-74)	64 (54 - 74)	65 (55 - 76)	67 (57-77)	56 (45 - 67)	60 (50 - 71)	63 (53 - 74)	66 (56 - 76)
**Deaths**	1252	2659	3821	3665	1433	1836	2332	2047
**Age at death**	79 (72-86)	79 (73 - 86)	80 (73 - 87)	80 (73 - 88)	79 (72 - 86)	80 (73 - 87)	81 (74 - 88)	82 (75 - 89)
**Body-mass index (%)**								
**< 25 kg/m2**	20.92%	17.18%	16.14%	16.91%	31.0%	28.8%	29.9%	34.3%
**Count**	4548	9771	10141	3164	2248	3660	3439	581
**25-30 kg/m2**	43.31%	39.18%	36.98%	37.21%	45.0%	44.1%	42.9%	42.5%
**Count**	9415	22289	23234	6964	3267	5607	4938	719
**>30 kg/m2**	35.77%	43.64%	46.88%	45.89%	24.0%	27.0%	27.2%	23.2%
**Count**	7776	24826	29456	8588	1740	3433	3133	393
**No BMI measurement**	40.9%	27.4%	45.5%	84.1%	92.2%	88.2%	90.2%	98.5%
**Age of DM diagnosis (years)**	58 (48 - 68)	57 (47 - 67)	56 (47 - 66)	54 (45 - 64)				
**Diabetes duration**								
**0-2 years**	26.8%	18.0%	11.5%	3.3%				
**Count**	9871	14098	13224	3824				
**2-5 years**	25.1%	26.1%	18.4%	9.9%				
**Count**	9220	20479	21218	11663				
**5-10 years**	26.2%	30.3%	30.5%	27.9%				
**Count**	9643	23717	35173	32685				
**10-20 years**	20.5%	22.7%	32.0%	45.6%				
**Count**	7550	17784	36853	53486				

**>20 years**	1.3%	3.0%	7.6%	13.4%	
**Count**	492	2313	8783	15687	

B)

	Diabetes				Non-diabetes			
**Year**	**2001**	**2007**	**2013**	**2018**	**2001**	**2007**	**2013**	**2018**
**Prevalent cohort, n**	31543	68596	99754	102202	79370	93947	105569	103046
**Cohort age (years)**	68 (57 - 79)	67 (55 - 80)	66 (53-79)	66 (53 - 79)	59 (47 - 72)	61 (48 - 74)	64 (50 - 78)	66 (53 - 80)
**Deaths**	1252	2450	3329	3089	1296	1762	2285	1996
**Age at death**	82 (76 - 89)	83 (72 - 95)	84 (78 - 91)	84 (77 - 91)	84 (78 - 90)	85 (79 - 91)	86 (80 - 92)	86 (80 - 92)
**Body-mass index (%)**								
**< 25 kg/m2**	21.4%	19.2%	17.7%	17.6%	40.6%	38.6%	40.1%	51.8%
**Count**	3800	9141	8995	2651	2668	4233	3901	536
**25-30 kg/m2**	33.0%	29.4%	28.4%	27.8%	33.0%	32.1%	31.7%	29.9%
**Count**	5859	13961	14431	4196	2171	3528	3085	309
**>30 kg/m2**	45.5%	51.4%	53.9%	54.6%	26.4%	29.3%	28.1%	18.3%
**Count**	8076	24421	27425	8223	1733	3219	2735	189
**No BMI measurement**	43.8%	30.7%	49.0%	85.3%	91.7%	88.3%	90.8%	99.0%
**Age of DM diagnosis (years)**	62 (51 - 73)	60 (48 - 72)	57 (45 - 70)	54 (42 - 67)				
**Diabetes duration**								
**0-2 years**	27.0%	18.9%	11.8%	3.4%				
**Count**	8520	12995	11810	3467				
**2-5 years**	25.1%	27.4%	18.4%	10.8%				
**Count**	7933	18781	18375	10993				
**5-10 years**	26.4%	29.5%	31.6%	27.9%				
**Count**	8315	20229	31509	28471				
**10-20 years**	20.3%	21.5%	30.9%	45.7%				
**Count**	6407	14770	30846	46659				
**>20 years**	1.2%	2.7%	7.2%	12.3%				
**Count**	368	1821	7214	12612				

Supplemental Table 2. ICD-10 codes for each underlying cause of death

**Underlying causes of death**	**ICD-10 Codes**
1) Malignant neoplasm of liver and intrahepatic bile ducts	C22
2) Malignant neoplasm of colon, rectosigmoid junction and rectum (i.e. colorectal)	C18, C19, C20
3) Malignant neoplasms of digestive organs except liver and intrahepatic bile ducts or colorectal	C15-C17,C21,C24, C26
Malignant neoplasm of gallbladder	C23
Malignant neoplasm of pancreas	C25
4) Malignant neoplasms of lymphoid, haematopoietic and related tissue	C81-C96
5) Malignant neoplasm of trachea, bronchus and lung	C33-C34
6) Malignant neoplasm of prostate	C61
7) Malignant neoplasm of breast	C50
8) Malignant neoplasm of cervix	C53
9) Malignant neoplasm of corpus uteri	C54
10) All other Neoplasms	C00-C14, C27-C32, C35-C49, C51-C52, C55-C60, C62-C80, C97-D48
11) Respiratory	J00-J99
12) Diabetes	E10-E14
13) Ischaemic heart disease	I20-I25
14) Stroke (cerebrovascular)	I60-I69
15) Other circulatory	I00-I19, I26-I59, I70-I99
16) Renal disease	N00-28
17) Liver disease	K70-77
18) Intentional Injury - Assault	X85-Y09, Y871
19) Intentional Injury - Self-harm	X60-X84, Y870
20) Intentional Injury - Other intentional injury	Y35, Y36
21) Unintentional Injury	V01-X59, Y40-Y86, Y88, Y89

22) Dementia and Alzheimers	F00-F03, G30
23) All other ICD codes	

Supplemental Table 3. Cause groupings according to underlying cause of death.

Underlying causes of death	Cause grouping - tier 1	Cause grouping - tier 2
1) Malignant neoplasm of liver and intrahepatic bile ducts	Cancer	DM-related cancers
2) Malignant neoplasm of colon, rectosigmoid junction and rectum (i.e. colorectal)	Cancer	DM-related cancers
3) Malignant neoplasms of digestive organs except liver and intrahepatic bile ducts or colorectal	Cancer	All other cancers
Malignant neoplasm of gallbladder	Cancer	DM-related cancers
Malignant neoplasm of pancreas	Cancer	DM-related cancers
4) Malignant neoplasms of lymphoid, haematopoietic and related tissue	Cancer	All other cancers
5) Malignant neoplasm of trachea, bronchus and lung	Cancer	All other cancers
6) Malignant neoplasm of prostate	Cancer	All other cancers
7) Malignant neoplasm of breast	Cancer	DM-related cancers
8) Malignant neoplasm of cervix	Cancer	All other cancers
9) Malignant neoplasm of corpus uteri	Cancer	DM-related cancers
10) All other Neoplasms	Cancer	All other cancers
11) Respiratory	Other	Respiratory
12) Diabetes	Other	Diabetes
13) Ischaemic heart disease	Vascular	Ischaemic heart disease
14) Stroke (cerebrovascular)	Vascular	Stroke
15) Other circulatory	Vascular	Other circulatory
16) Renal disease	Other	Renal disease
17) Liver disease	Other	Liver disease
18) Intentional Injury - Assault	Other	Accidents
19) Intentional Injury - Self-harm	Other	Accidents
20) Intentional Injury - Other intentional injury	Other	Accidents
21) Unintentional Injury	Other	Accidents
22) Dementia and Alzheimers	Other	Dementia
23) All other ICD codes	Other	Other

Supplemental Table 4. Adjusted all-cause mortality in 2001 and 2018 per 1,000 and average 10-year absolute change in death rate, in men and women with and without diabetes across Index of Multiple Deprivation Quintiles. The reported rates in 2001 and 2018 correspond to a population that has the same age distribution as the entire sample, those with and without diabetes, over the entire analysis period.

	Diabetes			Non-Diabetes		
	**2001**	**2018**		**2001**	**2018**	
Index of Multiple Deprivation Quintiles	**Rate per 1,000 (95% CI)**	**Rate per 1,000 (95% CI)**	**Average 10-year absolute change**	**Rate per 1,000 (95% CI)**	**Rate per 1,000 (95% CI)**	**Average 10-year absolute change**
**Quintile 1**	30.7 (27.6 - 33.7)	21.4 (19.3 - 23.6)	-5.4 (-4.4, -6.4)	20.6 (19.9 - 21.3)	13.0 (12.7 - 13.4)	-4.3 (-4.1, -4.5)
**Quintile 2**	35.7 (31.8 - 39.5)	26.9 (24.0 - 19.9)	-5.1 (-3.8, -6.4)	25.3 (24.3 - 26.3)	15.8 (15.3 - 16.3)	-5.5 (-5.2, -5.7)
**Quintile 3**	44.2 (40.0 - 48.4)	32.4 (29.3 - 35.5)	-6.9 (-5.4, -8.3)	31.0 (29.9 - 32.0)	19.7 (19.1 - 20.3)	-6.5 (-6.2, -6.8)
**Quintile 4**	40.8 (39.6 - 42.2)	28.0 (27.2 - 28.8)	-7.5 (-7.1, -7.8)	30.0 (28.9 - 31.0)	19.3 (18.7 - 19.9)	-6.1 (-5.8, -6.4)
**Quintile 5**	46.4 (44.9 - 47.8)	32.8 (31.8 - 33.8)	-7.9 (-7.4, -8.3)	33.6 (32.3 - 35.0)	22.0 (21.2 - 22.8)	-6.7 (-6.3, -7.1)

Supplemental Table 5. Proportional contribution to mortality burden of cause-specific groupings in diabetes and non-diabetes population.

	2001	2002	2003	2004	2005	2006	2007	2008	2009	2010	2011	2012	2013	2014	2015	2016	2017	2018
**Diabetes population**																		
**IHD**	24.1%	23.3%	22.4%	21.6%	20.8%	19.9%	19.1%	18.3%	17.4%	16.6%	15.8%	15.0%	14.2%	13.4%	12.7%	11.9%	11.2%	10.5%
**Stroke**	12.5%	12.1%	11.7%	11.3%	10.9%	10.5%	10.1%	9.7%	9.3%	8.9%	8.5%	8.1%	7.7%	7.3%	6.9%	6.5%	6.1%	5.8%
**Renal**	1.0%	1.0%	1.0%	1.0%	0.9%	0.9%	0.9%	0.9%	0.8%	0.8%	0.8%	0.7%	0.7%	0.7%	0.7%	0.6%	0.6%	0.6%
**Liver**	1.1%	1.1%	1.2%	1.3%	1.3%	1.4%	1.4%	1.5%	1.5%	1.6%	1.6%	1.7%	1.7%	1.8%	1.8%	1.8%	1.9%	1.9%
**Respiratory**	11.1%	11.4%	11.6%	11.9%	12.1%	12.4%	12.6%	12.8%	12.9%	13.1%	13.2%	13.3%	13.4%	13.5%	13.5%	13.5%	13.5%	13.4%
**Diabetes**	8.6%	8.2%	7.8%	7.4%	7.0%	6.7%	6.3%	6.0%	5.6%	5.3%	5.0%	4.7%	4.4%	4.1%	3.8%	3.5%	3.3%	3.0%
**Dementia**	2.0%	2.3%	2.6%	3.0%	3.5%	3.9%	4.5%	5.1%	5.8%	6.5%	7.4%	8.3%	9.3%	10.5%	11.7%	13.1%	14.6%	16.2%
**Injuries**	0.9%	1.0%	1.0%	1.0%	1.0%	1.1%	1.1%	1.1%	1.1%	1.1%	1.1%	1.1%	1.1%	1.2%	1.2%	1.2%	1.2%	1.1%
**Other cancer**	14.7%	15.1%	15.6%	16.0%	16.4%	16.7%	17.1%	17.4%	17.8%	18.1%	18.3%	18.5%	18.7%	18.9%	19.0%	19.1%	19.1%	19.0%
**Diabetes-related cancer**	6.8%	7.0%	7.2%	7.4%	7.6%	7.8%	8.0%	8.2%	8.3%	8.5%	8.6%	8.7%	8.8%	8.9%	9.0%	9.0%	9.0%	9.0%
**Other circulatory**	7.3%	7.4%	7.6%	7.7%	7.8%	7.9%	8.0%	8.1%	8.1%	8.2%	8.2%	8.2%	8.2%	8.2%	8.2%	8.1%	8.0%	8.0%
**Other**	9.8%	10.0%	10.2%	10.4%	10.6%	10.8%	11.0%	11.2%	11.3%	11.4%	11.5%	11.6%	11.7%	11.7%	11.7%	11.7%	11.6%	11.5%
**Non-diabetes population**																		
**IHD**	21.3%	20.6%	19.9%	19.2%	18.6%	17.9%	17.3%	16.6%	16.0%	15.3%	14.7%	14.1%	13.5%	12.9%	12.3%	11.7%	11.1%	10.6%
**Stroke**	13.5%	13.0%	12.6%	12.1%	11.6%	11.2%	10.7%	10.3%	9.9%	9.4%	9.0%	8.6%	8.2%	7.8%	7.4%	7.0%	6.7%	6.3%
**Renal**	1.2%	1.1%	1.1%	1.1%	1.1%	1.1%	1.1%	1.0%	1.0%	1.0%	1.0%	0.9%	0.9%	0.9%	0.9%	0.8%	0.8%	0.8%
**Liver**	0.7%	0.7%	0.8%	0.8%	0.8%	0.9%	0.9%	1.0%	1.0%	1.0%	1.1%	1.1%	1.1%	1.2%	1.2%	1.3%	1.3%	1.3%
**Respiratory**	12.9%	13.1%	13.2%	13.3%	13.4%	13.5%	13.6%	13.7%	13.7%	13.8%	13.8%	13.8%	13.8%	13.7%	13.7%	13.6%	13.5%	13.4%
**Diabetes**	0.4%	0.4%	0.4%	0.4%	0.4%	0.4%	0.4%	0.4%	0.4%	0.4%	0.4%	0.4%	0.4%	0.4%	0.4%	0.4%	0.4%	0.4%
**Dementia**	3.1%	3.5%	3.9%	4.3%	4.8%	5.3%	5.8%	6.4%	7.1%	7.8%	8.6%	9.5%	10.4%	11.4%	12.5%	13.6%	14.9%	16.2%
**Injuries**	1.4%	1.4%	1.4%	1.5%	1.5%	1.5%	1.5%	1.6%	1.6%	1.6%	1.6%	1.7%	1.7%	1.7%	1.7%	1.7%	1.7%	1.7%
**Other cancer**	19.1%	19.3%	19.5%	19.7%	19.9%	20.1%	20.2%	20.3%	20.4%	20.5%	20.5%	20.6%	20.6%	20.5%	20.5%	20.4%	20.2%	20.1%
**Diabetes-related cancer**	5.3%	5.4%	5.5%	5.6%	5.7%	5.8%	5.9%	6.0%	6.1%	6.2%	6.3%	6.3%	6.4%	6.5%	6.5%	6.5%	6.6%	6.6%

**Other circulatory**	8.6%	8.7%	8.7%	8.8%	8.8%	8.8%	8.8%	8.8%	8.8%	8.7%	8.7%	8.7%	8.6%	8.5%	8.5%	8.4%	8.3%	8.1%
**Other**	12.4%	12.7%	12.9%	13.1%	13.3%	13.5%	13.6%	13.8%	14.0%	14.1%	14.2%	14.3%	14.4%	14.5%	14.5%	14.5%	14.5%	14.5%

Supplemental Table 6. Site-specific proportions, and number of deaths, of cancer mortality burden in 2001 and 2018 in A) men and B) women with and without diabetes. Denominator for site-specific proportions of cancer mortality is total number of cancer deaths for given year, sex and population group.

A)

	**Diabetes**				**Non-Diabetes**			
	**2001**		**2018**		**2001**		**2018**	
Cancer site	Proportion	Count	Proportion	Count	Proportion	Count	Proportion	Count
**Liver**	4.4%	12	5.9%	56	1.4%	7	3.1%	20
**Colorectal**	8.8%	24	8.3%	79	7.9%	41	7.8%	50
**Other digestive organs**	7.3%	20	10.8%	103	9.5%	49	10.6%	68
**Lymphoid**	9.1%	25	8.5%	81	10.4%	54	11.0%	71
**Gallbladder**	0.0%	0	0.0%	0	0.2%	1	0.2%	1
**Pancreas**	12.8%	35	8.0%	76	4.1%	21	4.5%	29
**Lung**	21.2%	58	19.3%	184	22.1%	114	21.3%	137
**Prostate**	8.4%	23	15.0%	143	15.7%	81	15.4%	99
**Breast**	0.4%	1	0.0%	0	0.2%	1	0.3%	2
**All other cancers**	27.7%	76	24.2%	230	28.6%	148	25.9%	167

B)

	Diabetes				Non-Diabetes			
	2001		2018		2001		2018	
Cancer site	Proportion	Count	Proportion	Count	Proportion	Count	Proportion	Count
**Liver**	0.4%	1	3.0%	29	1.2%	6	1.9%	12
**Colorectal**	8.0%	22	7.9%	75	5.8%	30	7.6%	49
**Other Digestive organs**	5.8%	16	5.0%	48	5.2%	27	5.6%	36
**Lymphoid**	8.4%	23	4.1%	39	5.6%	29	6.4%	41
**Gallbladder**	1.1%	3	0.4%	4	0.2%	1	0.3%	2
**Pancreas**	9.1%	25	4.6%	44	2.3%	12	3.9%	25
**Lung**	9.1%	25	12.0%	114	13.2%	68	14.4%	93
**Breast**	15.0%	41	9.0%	86	11.0%	57	11.8%	76
**Endometrial**	1.5%	4	1.9%	18	1.7%	9	2.3%	15
**Cervix**	0.4%	1	0.4%	4	0.8%	4	1.1%	7
**All other cancers**	27.4%	75	15.0%	143	23.4%	121	18.3%	118

Supplemental Table 7. Adjusted all-cause and cause-specific annual mortality rates from 2001-2018 per 1,000 in men and women with and without diabetes. Table 7a. Diabetes Men; Table 7b. Diabetes Women; 7c. Non-Diabetes Men; 7d. Non-Diabetes Women. The reported rates in each year correspond to a population that has the same age distribution as the entire sample, those with and without diabetes, over the entire analysis period. Diabetes-associated cancers= colorectal, pancreatic, liver, gallbladder, breast and endometrial cancers.

7a.

	2001	2002	2003	2004	2005	2006	2007	2008	2009	2010	2011	2012	2013	2014	2015	2016	2017	2018
**Cause**	**Rate per 1,000**	**Rate per 1,000**	**Rate per 1,000**	**Rate per 1,000**	**Rate per 1,000**	**Rate per 1,000**	**Rate per 1,000**	**Rate per 1,000**	**Rate per 1,000**	**Rate per 1,000**	**Rate per 1,000**	**Rate per 1,000**	**Rate per 1,000**	**Rate per 1,000**	**Rate per 1,000**	**Rate per 1,000**	**Rate per 1,000**	**Rate per 1,000**
All-cause	40.7	39.8	38.9	38.0	37.2	36.4	35.6	34.8	34.0	33.2	32.5	31.8	31.1	30.4	29.7	29.1	28.4	27.8
IHD	11.2	10.5	9.9	9.3	8.7	8.2	7.7	7.3	6.8	6.4	6.1	5.7	5.4	5.0	4.7	4.5	4.2	3.9
Stroke	4.7	4.4	4.1	3.9	3.6	3.4	3.2	3.0	2.8	2.6	2.4	2.3	2.1	2.0	1.9	1.7	1.6	1.5
Renal	0.4	0.4	0.4	0.4	0.3	0.3	0.3	0.3	0.3	0.3	0.2	0.2	0.2	0.2	0.2	0.2	0.2	0.2
Liver	0.6	0.6	0.6	0.6	0.6	0.6	0.6	0.6	0.6	0.6	0.6	0.6	0.7	0.7	0.7	0.7	0.7	0.7
Respiratory	4.7	4.6	4.6	4.5	4.5	4.5	4.4	4.4	4.3	4.3	4.2	4.2	4.2	4.1	4.1	4.0	4.0	4.0
Diabetes	3.1	2.9	2.7	2.5	2.3	2.1	1.9	1.8	1.7	1.5	1.4	1.3	1.2	1.1	1.0	1.0	0.9	0.8
Dementia	0.6	0.7	0.8	0.9	1.0	1.1	1.2	1.3	1.5	1.6	1.8	2.0	2.2	2.5	2.7	3.1	3.4	3.8
Injuries	0.4	0.4	0.4	0.4	0.4	0.4	0.4	0.4	0.4	0.4	0.4	0.4	0.4	0.4	0.4	0.4	0.4	0.4
Other cancer	7.9	7.9	7.8	7.8	7.7	7.7	7.6	7.6	7.5	7.5	7.4	7.4	7.3	7.3	7.2	7.2	7.1	7.1
DM-related cancer	2.6	2.6	2.5	2.5	2.5	2.5	2.5	2.4	2.4	2.4	2.4	2.4	2.4	2.3	2.3	2.3	2.3	2.3
Other circulatory	2.9	2.9	2.8	2.8	2.7	2.7	2.6	2.6	2.6	2.5	2.5	2.4	2.4	2.4	2.3	2.3	2.2	2.2
Other	3.4	3.4	3.4	3.4	3.3	3.3	3.3	3.3	3.3	3.3	3.3	3.3	3.3	3.3	3.3	3.2	3.2	3.2

7b.

Cause	2001 Rate per 1,000	2002 Rate per 1,000	2003 Rate per 1,000	2004 Rate per 1,000	2005 Rate per 1,000	2006 Rate per 1,000	2007 Rate per 1,000	2008 Rate per 1,000	2009 Rate per 1,000	2010 Rate per 1,000	2011 Rate per 1,000	2012 Rate per 1,000	2013 Rate per 1,000	2014 Rate per 1,000	2015 Rate per 1,000	2016 Rate per 1,000	2017 Rate per 1,000	2018 Rate per 1,000
All-cause	42.7	41.7	40.8	40.0	39.1	38.3	37.4	36.6	35.8	35.1	34.3	33.6	32.8	32.1	31.4	30.8	30.1	29.5
IHD	9.9	9.1	8.4	7.7	7.1	6.6	6.1	5.6	5.2	4.8	4.4	4.1	3.8	3.5	3.2	3.0	2.7	2.5
Stroke	6.4	6.0	5.6	5.3	4.9	4.6	4.3	4.1	3.8	3.6	3.3	3.1	2.9	2.8	2.6	2.4	2.3	2.1
Renal	0.5	0.4	0.4	0.4	0.4	0.4	0.3	0.3	0.3	0.3	0.3	0.3	0.2	0.2	0.2	0.2	0.2	0.2
Liver	0.3	0.4	0.4	0.4	0.4	0.4	0.4	0.4	0.4	0.4	0.4	0.4	0.4	0.5	0.5	0.5	0.5	0.5
Respiratory	5.1	5.0	5.0	4.9	4.9	4.9	4.8	4.8	4.7	4.7	4.7	4.6	4.6	4.6	4.5	4.5	4.4	4.4
Diabetes	4.5	4.1	3.8	3.5	3.2	3.0	2.7	2.5	2.3	2.1	2.0	1.8	1.7	1.5	1.4	1.3	1.2	1.1
Dementia	1.2	1.3	1.4	1.6	1.8	2.0	2.2	2.4	2.6	2.9	3.2	3.6	4.0	4.4	4.8	5.4	5.9	6.6
Injuries	0.4	0.4	0.4	0.4	0.4	0.4	0.4	0.4	0.4	0.4	0.4	0.4	0.3	0.3	0.3	0.3	0.3	0.3
Other cancer	4.8	4.8	4.8	4.8	4.8	4.8	4.8	4.7	4.7	4.7	4.7	4.7	4.7	4.7	4.7	4.7	4.7	4.6
DM-related cancer	3.5	3.5	3.5	3.5	3.5	3.5	3.5	3.5	3.5	3.5	3.5	3.5	3.5	3.5	3.5	3.5	3.5	3.5
Other circulatory	3.5	3.4	3.4	3.4	3.3	3.3	3.2	3.2	3.1	3.1	3.1	3.0	3.0	3.0	2.9	2.9	2.8	2.8
Other	5.3	5.2	5.1	5.1	5.0	4.9	4.8	4.7	4.7	4.6	4.5	4.4	4.4	4.3	4.2	4.2	4.1	4.0

7c.

Cause	2001 Rate per 1,000	2002 Rate per 1,000	2003 Rate per 1,000	2004 Rate per 1,000	2005 Rate per 1,000	2006 Rate per 1,000	2007 Rate per 1,000	2008 Rate per 1,000	2009 Rate per 1,000	2010 Rate per 1,000	2011 Rate per 1,000	2012 Rate per 1,000	2013 Rate per 1,000	2014 Rate per 1,000	2015 Rate per 1,000	2016 Rate per 1,000	2017 Rate per 1,000	2018 Rate per 1,000
All-cause	28.4	27.5	26.6	25.8	25.0	24.3	23.5	22.8	22.1	21.4	20.7	20.1	19.5	18.9	18.3	17.7	17.2	16.7
IHD	6.6	6.2	5.8	5.4	5.1	4.8	4.5	4.2	3.9	3.7	3.4	3.2	3.0	2.8	2.6	2.5	2.3	2.2
Stroke	3.3	3.0	2.8	2.6	2.4	2.2	2.1	1.9	1.8	1.7	1.5	1.4	1.3	1.2	1.1	1.0	1.0	0.9
Renal	0.4	0.3	0.3	0.3	0.3	0.3	0.3	0.2	0.2	0.2	0.2	0.2	0.2	0.2	0.2	0.1	0.1	0.1
Liver	0.3	0.3	0.3	0.3	0.3	0.3	0.3	0.3	0.3	0.3	0.3	0.3	0.3	0.3	0.3	0.3	0.3	0.3
Respiratory	3.5	3.4	3.4	3.3	3.2	3.1	3.0	2.9	2.9	2.8	2.7	2.6	2.6	2.5	2.4	2.4	2.3	2.2
Diabetes	0.1	0.1	0.1	0.1	0.1	0.1	0.1	0.1	0.1	0.1	0.1	0.1	0.1	0.1	0.1	0.1	0.1	0.1
Dementia	0.7	0.7	0.7	0.8	0.8	0.9	0.9	1.0	1.1	1.1	1.2	1.3	1.3	1.4	1.5	1.6	1.7	1.8
Injuries	0.4	0.4	0.4	0.4	0.4	0.4	0.4	0.4	0.4	0.4	0.4	0.4	0.4	0.4	0.4	0.4	0.3	0.3
Other cancer	7.8	7.5	7.3	7.1	6.9	6.7	6.4	6.3	6.1	5.9	5.7	5.5	5.3	5.2	5.0	4.9	4.7	4.6
DM-related cancer	0.3	0.3	0.3	0.3	0.3	0.3	0.3	0.3	0.3	0.3	0.3	0.2	0.2	0.2	0.2	0.2	0.2	0.2
Other circulatory	2.3	2.2	2.1	2.1	2.0	1.9	1.8	1.8	1.7	1.6	1.6	1.5	1.4	1.4	1.3	1.3	1.2	1.2
Other	3.1	3.0	3.0	2.9	2.8	2.8	2.7	2.7	2.6	2.6	2.6	2.5	2.5	2.4	2.4	2.3	2.3	2.3

7d.

Cause	2001 Rate per 1,000	2002 Rate per 1,000	2003 Rate per 1,000	2004 Rate per 1,000	2005 Rate per 1,000	2006 Rate per 1,000	2007 Rate per 1,000	2008 Rate per 1,000	2009 Rate per 1,000	2010 Rate per 1,000	2011 Rate per 1,000	2012 Rate per 1,000	2013 Rate per 1,000	2014 Rate per 1,000	2015 Rate per 1,000	2016 Rate per 1,000	2017 Rate per 1,000	2018 Rate per 1,000
All-cause	28.2	27.5	26.8	26.2	25.6	25.0	24.4	23.8	23.2	22.7	22.1	21.6	21.1	20.6	20.1	19.6	19.1	18.7
IHD	5.7	5.3	4.8	4.5	4.1	3.8	3.5	3.2	3.0	2.7	2.5	2.3	2.1	2.0	1.8	1.7	1.5	1.4
Stroke	4.6	4.3	4.0	3.7	3.4	3.2	3.0	2.7	2.5	2.4	2.2	2.0	1.9	1.7	1.6	1.5	1.4	1.3
Renal	0.3	0.3	0.3	0.3	0.3	0.2	0.2	0.2	0.2	0.2	0.2	0.2	0.2	0.2	0.2	0.2	0.1	0.1
Liver	0.1	0.1	0.1	0.1	0.1	0.1	0.1	0.1	0.1	0.2	0.2	0.2	0.2	0.2	0.2	0.2	0.2	0.2
Respiratory	4.0	3.9	3.8	3.6	3.5	3.4	3.3	3.2	3.1	3.0	2.9	2.8	2.7	2.7	2.6	2.5	2.4	2.4
Diabetes	0.1	0.1	0.1	0.1	0.1	0.1	0.1	0.1	0.1	0.1	0.1	0.1	0.1	0.1	0.1	0.1	0.0	0.0
Dementia	1.2	1.3	1.4	1.5	1.6	1.7	1.8	2.0	2.1	2.2	2.4	2.6	2.8	2.9	3.2	3.4	3.6	3.9
Injuries	0.4	0.4	0.4	0.4	0.3	0.3	0.3	0.3	0.3	0.3	0.3	0.3	0.3	0.3	0.3	0.3	0.2	0.2
Other cancer	4.8	4.7	4.6	4.5	4.4	4.3	4.2	4.1	4.0	4.0	3.9	3.8	3.7	3.7	3.6	3.5	3.4	3.4
DM-related cancer	1.2	1.2	1.2	1.2	1.2	1.1	1.1	1.1	1.1	1.1	1.0	1.0	1.0	1.0	1.0	0.9	0.9	0.9
Other circulatory	2.7	2.6	2.6	2.5	2.4	2.3	2.3	2.2	2.1	2.1	2.0	2.0	1.9	1.8	1.8	1.7	1.7	1.6
Other	4.2	4.1	4.0	3.9	3.8	3.7	3.6	3.5	3.5	3.4	3.3	3.2	3.1	3.0	3.0	2.9	2.8	2.8

Supplemental Table 8. Mortality rates per 1,000 and decline in mortality over study periods in CPRD study populations (unstandardised and standardised) and in the PHE report.

	2001	2016	2018	Decline in mortality rate 2001-2016	Decline in mortality rate 2001-2018
	Rate per 1,000 (95% CI)	Rate per 1,000 (95% CI)	Rate per 1,000 (95% CI)		
**Unstandardised CPRD study populations**					
Our study DM population	41.6 (41.0 - 42.2)		28.6 (28.3 - 29.0)		31.4%
Our study NDM population	28.3 (27.8 - 28.8)		17.6 (17.3 - 17.8)		37.8%
**Standardised CPRD study populations**					
Our study DM population standardised to the European standard population	19.9 (19.5 - 20.0)	14.0 (13.8 - 14.2)		29.7%	
Our study NDM population standardised to the European standard population	12.9 (12.7 - 13.1)	9.0 (8.8 - 9.1)		30.7%	
**PHE report**					
PHE	12.3 (12.3-12.3)	9.6 (9.6 - 9.6)		22.0%	

*PHE report = Public Health England. A review of recent trends in mortality in England. December 2018. https://assets.publishing.service.gov.uk/government/uploads/system/uploads/attachment_data/file/827518/Recent_trends_in_mortality_in_England.pdf

Supplemental Figure 1. Death rates due to vascular (blue), cancer (red), other-causes (green-orange) among those with diabetes (DM) and without diabetes (NDM), men and women in 2001 and 2018.

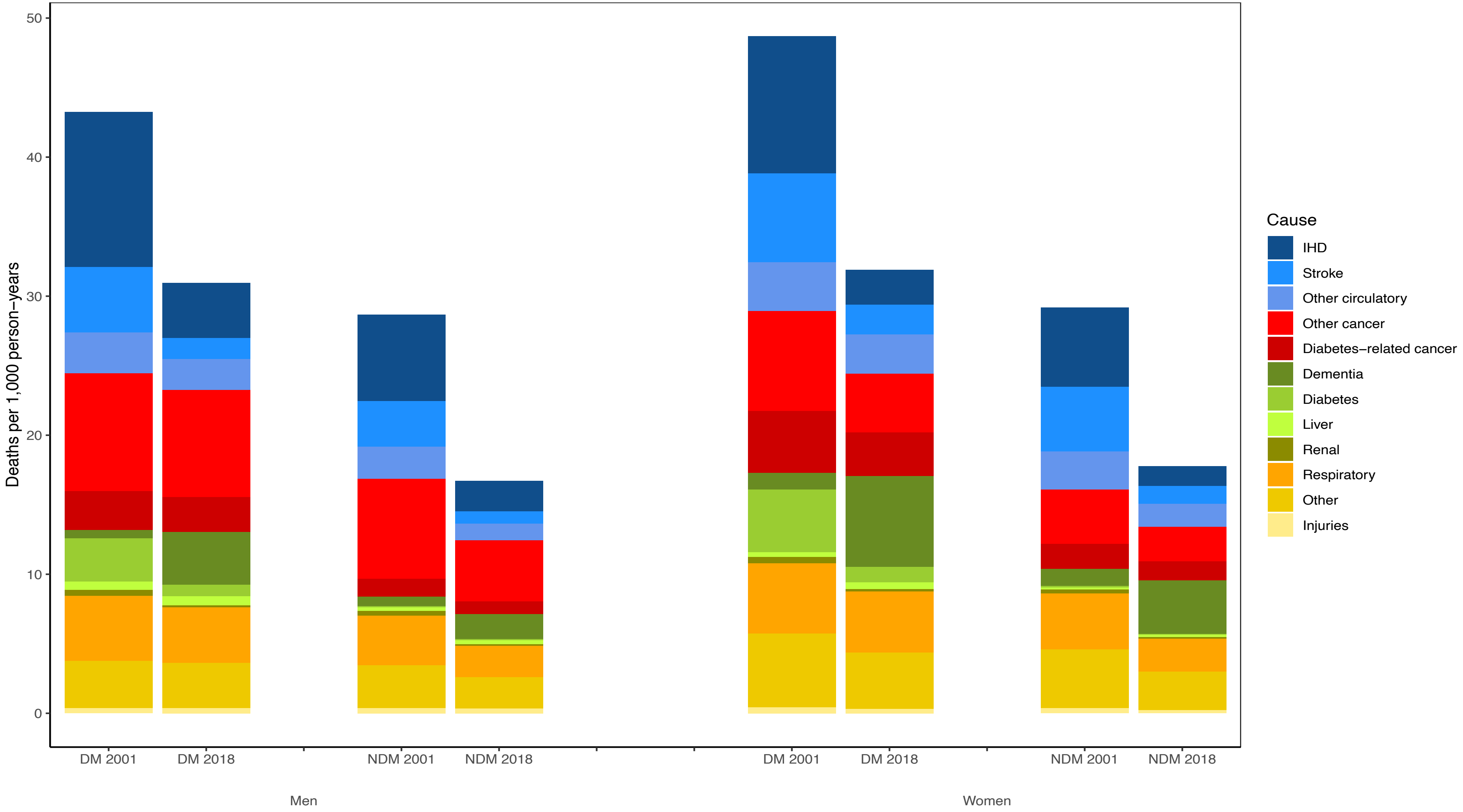

Supplemental Figure 2. Absolute change in cause-specific death rates from 2001 to 2018 by sex among those with diabetes (DM) and without diabetes (NDM).

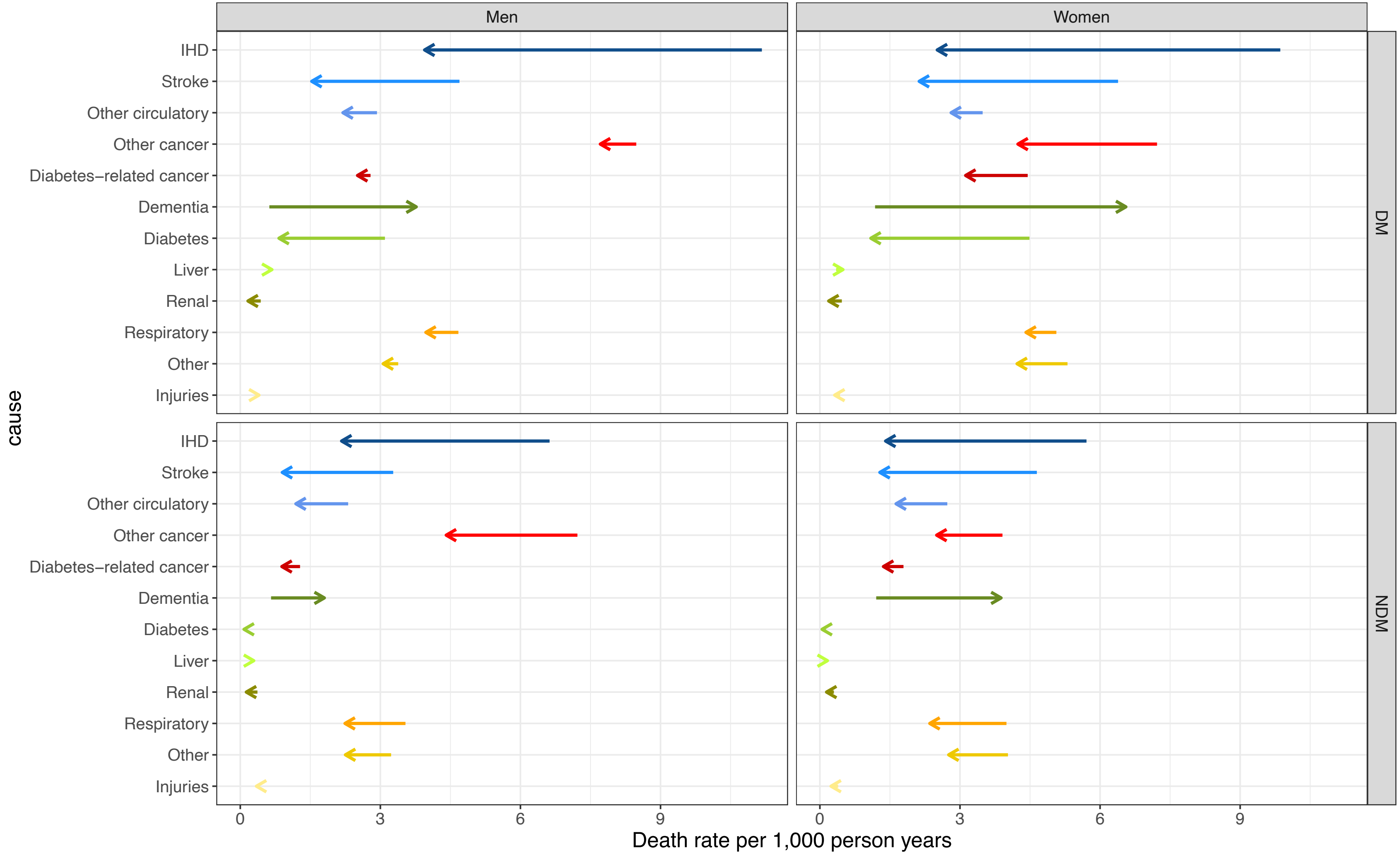

Supplemental Figure 3. Relative change in cause-specific death rates from 2001-2018 by sex in those with diabetes compared to those without diabetes

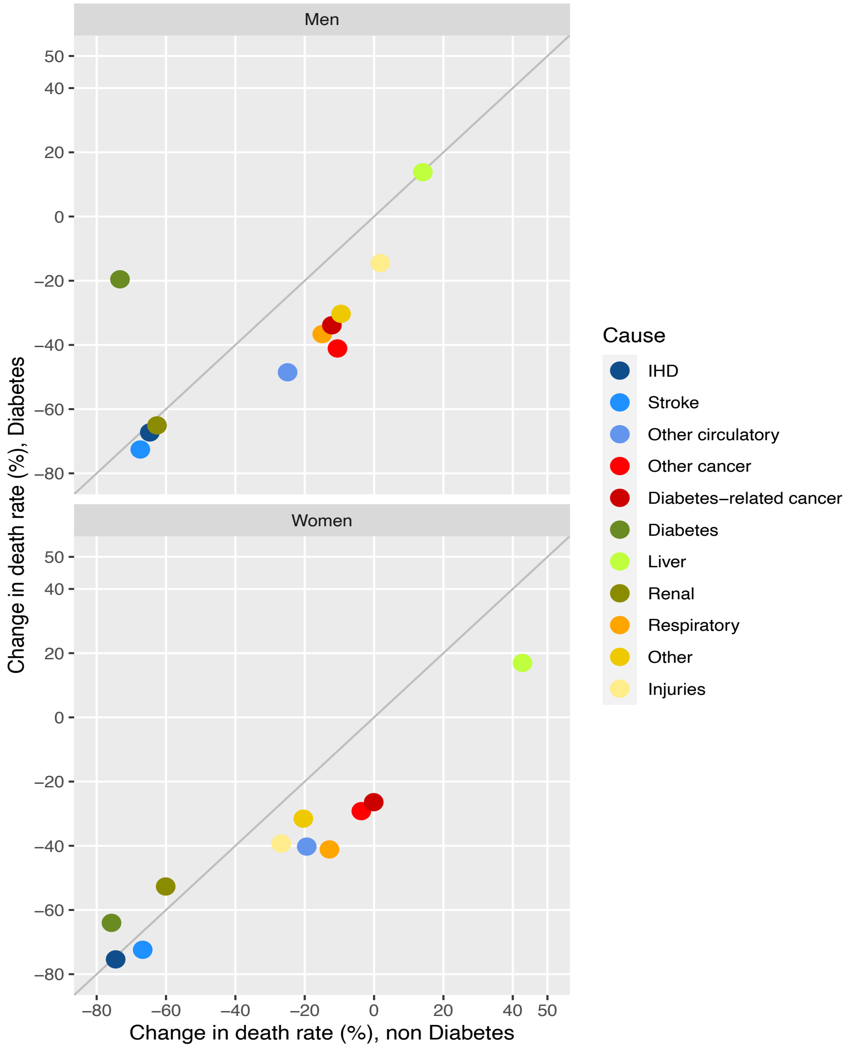

Supplemental Figure 4. Proportional contribution to mortality burden of cause-specific groupings by age group in diabetes (DM) and non-diabetes (NDM) population by age group

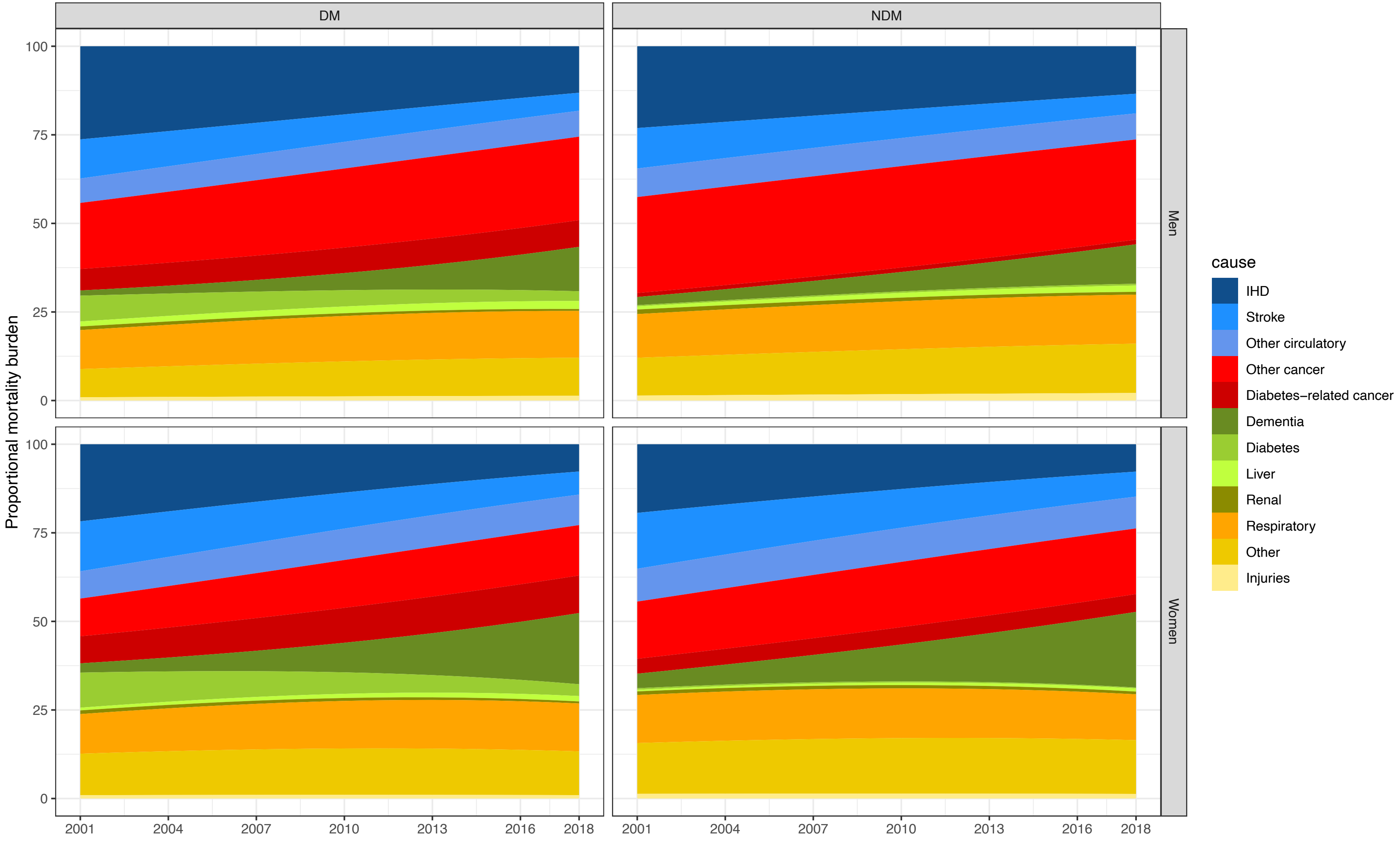

Supplemental Figure 5. Absolute change in cause-specific death rates from 2001 to 2018 by age among those with diabetes (DM) and without diabetes (NDM).

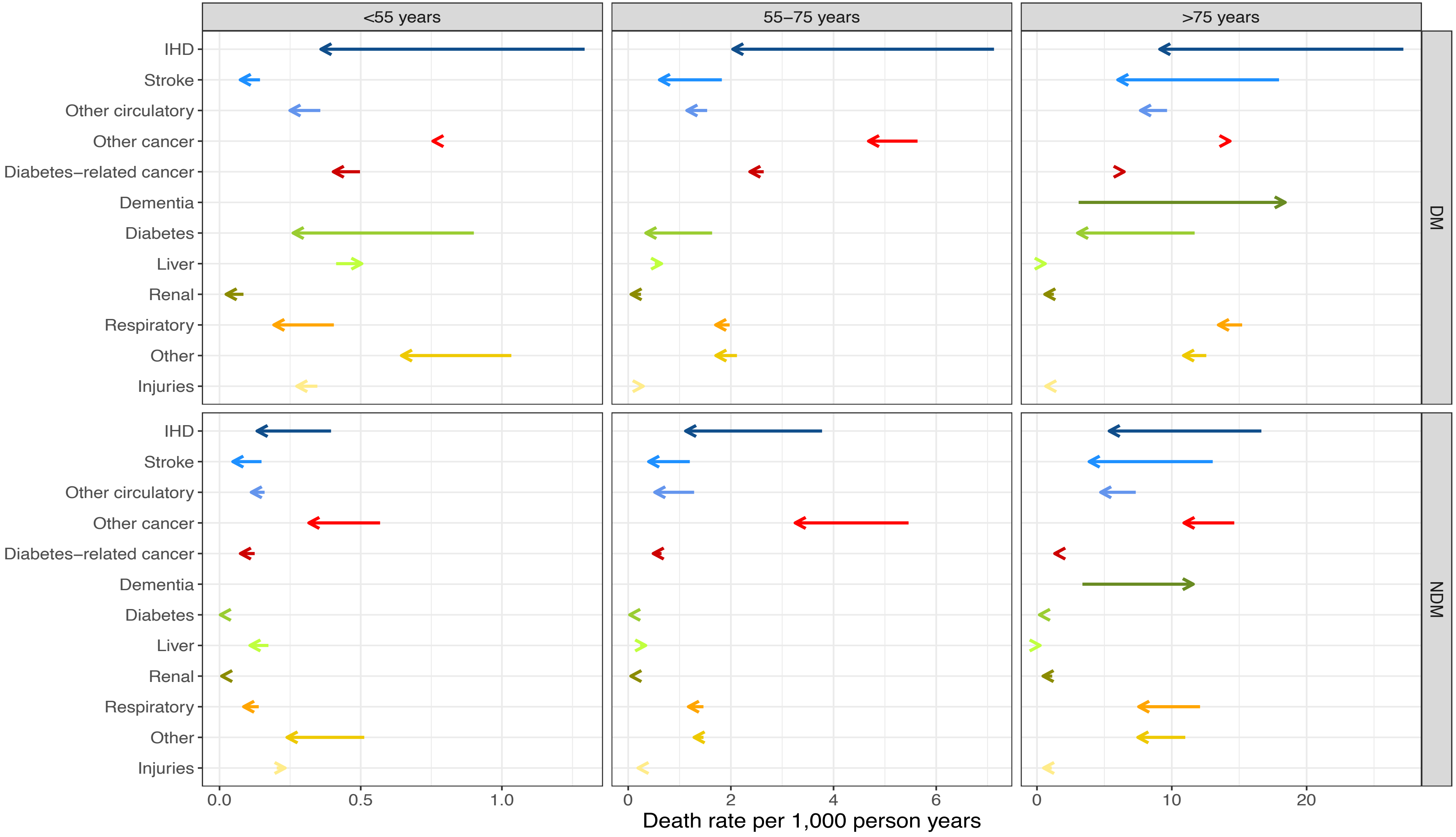

Supplemental Figure 6. Relative change in cause-specific death rates from 2001-2018 by age group in those with diabetes compared to those without diabetes.

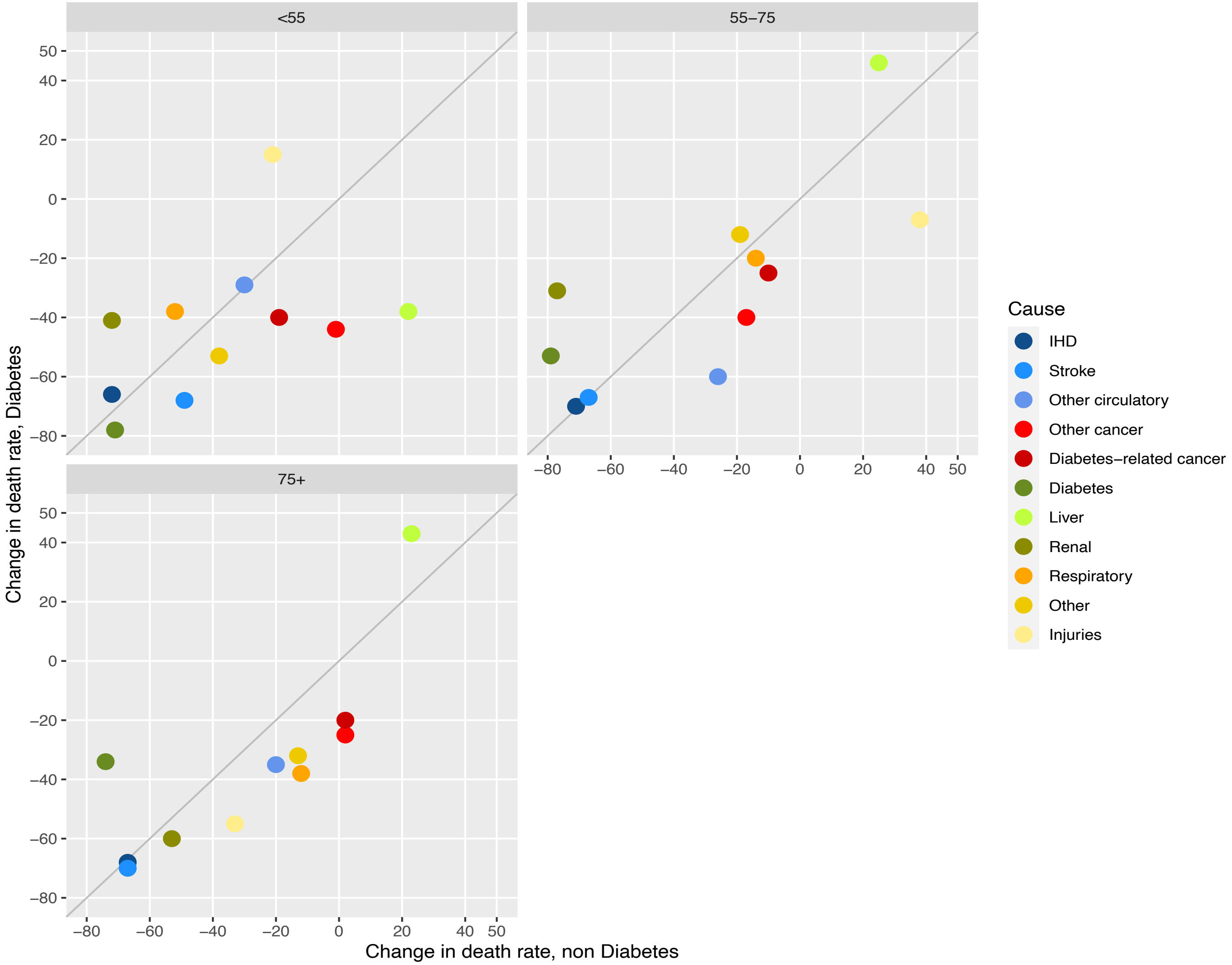

Supplemental Figure 7. Proportional contribution to mortality burden of cause-specific groupings by age group in diabetes (DM) and non-diabetes (NDM) population by age group.

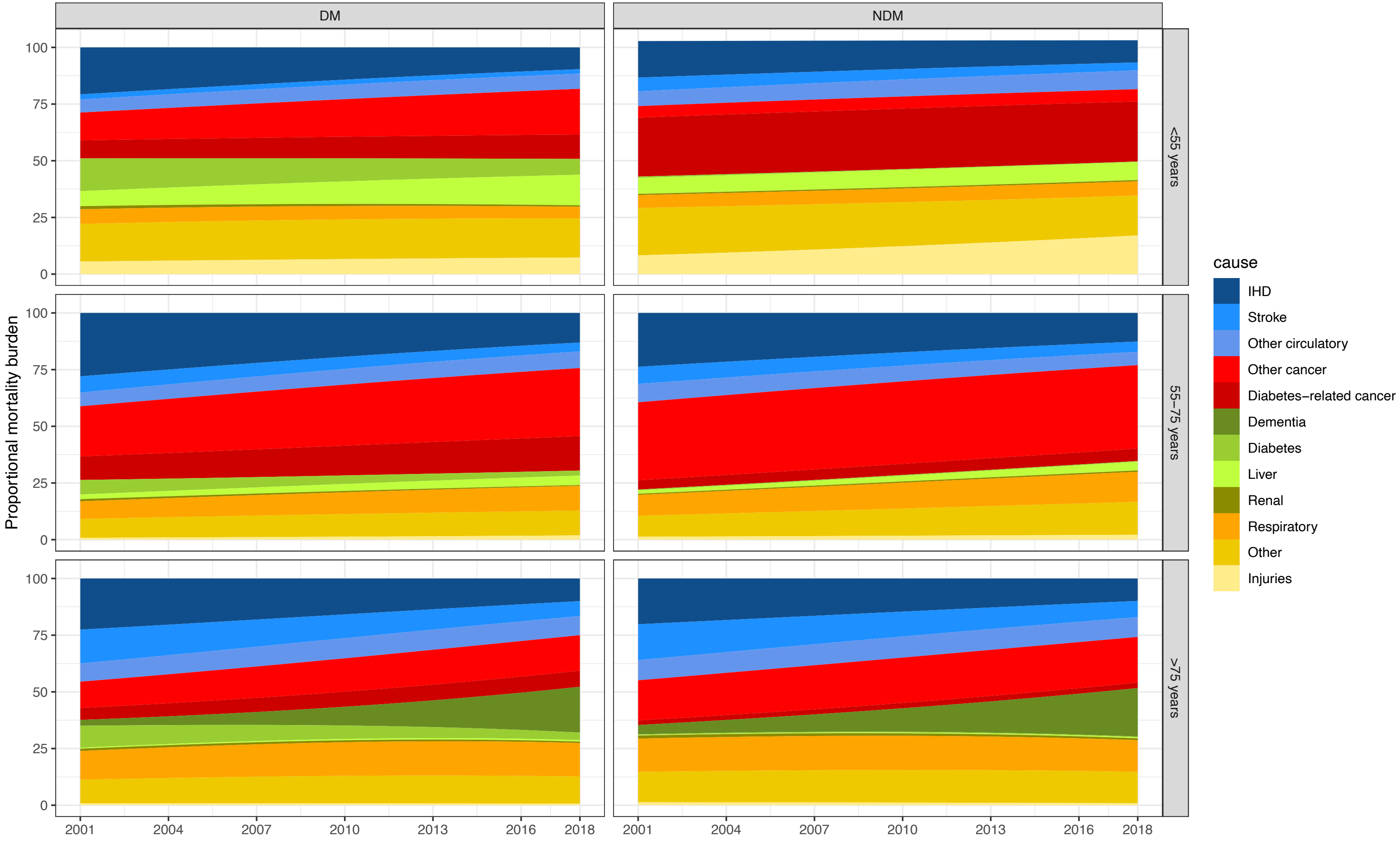

Supplemental Figure 8. Absolute change in cause-specific death rates from 2001 to 2018 by age among those with diabetes (DM) and without diabetes (NDM).

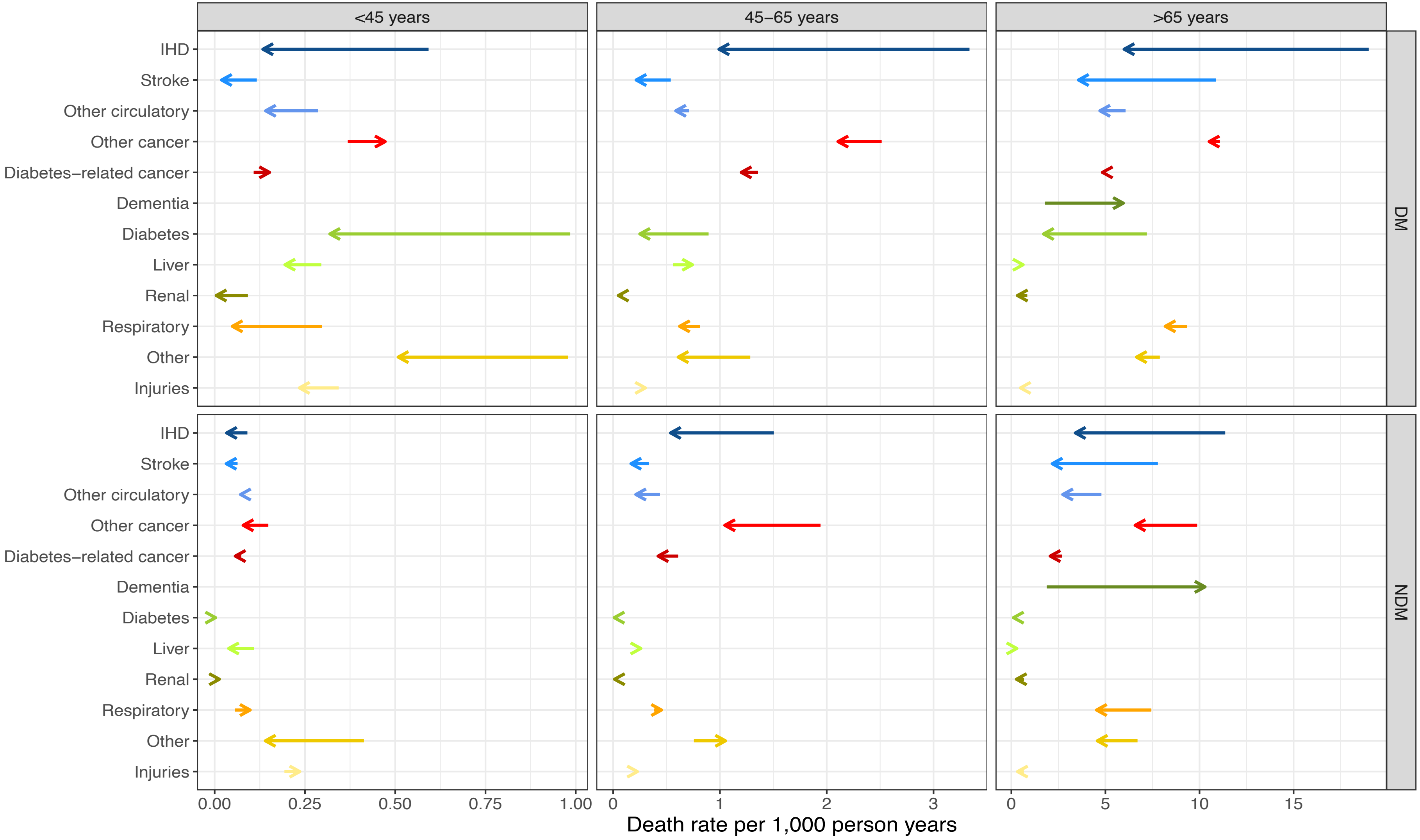

Supplemental Table 9. Clinical 'readcodes' and 'prodcodes' used to identify individuals with diabetes in the CPRD database

Readcodes

medcode	readcode	readterm	databasebuild
506	C100112	Non-insulin dependent diabetes mellitus	Feb-09
758	C10F.00	Type 2 diabetes mellitus	Feb-09
1407	C10FJ00	Insulin treated Type 2 diabetes mellitus	Feb-09
1684	66A4.00	Diabetic on oral treatment	Feb-09
4513	C109.00	Non-insulin dependent diabetes mellitus	Feb-09
5884	C109.11	NIDDM - Non-insulin dependent diabetes mellitus	Feb-09
8403	C109700	Non-insulin dependent diabetes mellitus - poor control	Feb-09
11047	66AH000	Conversion to insulin	Feb-09
12640	C10FC00	Type 2 diabetes mellitus with nephropathy	Feb-09
12736	C10F500	Type 2 diabetes mellitus with gangrene	Feb-09
14803	C100100	Diabetes mellitus	
17262	C109600	Non-insulin-dependent diabetes mellitus with retinopathy	Feb-09
17859	C109.12	Type 2 diabetes mellitus	Feb-09
18143	C109G11	Type II diabetes mellitus with arthropathy	Feb-09
18209	C109012	Type 2 diabetes mellitus with renal complications	Feb-09
18219	C109.13	Type II diabetes mellitus	Feb-09
18264	C109J12	Insulin treated Type II diabetes mellitus	Feb-09
18278	C109J00	Insulin treated Type 2 diabetes mellitus	Feb-09
18390	C10FM00	Type 2 diabetes mellitus with persistent microalbuminuria	Feb-09
18425	C10FB00	Type 2 diabetes mellitus with polyneuropathy	Feb-09
18496	C10F600	Type 2 diabetes mellitus with retinopathy	Feb-09

18777	C10F000	Type 2 diabetes mellitus with renal complications	Feb-09
22884	C10F.11	Type II diabetes mellitus	Feb-09
24458	C109711	Type II diabetes mellitus - poor control	Feb-09
24693	C109G00	Non-insulin dependent diabetes mellitus with arthropathy	Feb-09
24836	C109C12	Type 2 diabetes mellitus with nephropathy	Feb-09
25591	C10FQ00	Type 2 diabetes mellitus with exudative maculopathy	Feb-09
25627	C10F700	Type 2 diabetes mellitus - poor control	Feb-09
26054	C10FL00	Type 2 diabetes mellitus with persistent proteinuria	Feb-09
29979	C109900	Non-insulin-dependent diabetes mellitus without complication	Feb-09
32627	C10FN00	Type 2 diabetes mellitus with ketoacidosis	Feb-09
33807	C107200	Diabetes mellitus	
34268	C10F200	Type 2 diabetes mellitus with neurological complications	Feb-09
34450	C10FK00	Hyperosmolar non-ketotic state in type 2 diabetes mellitus	Feb-09
34912	C109400	Non-insulin dependent diabetes mellitus with ulcer	Feb-09
35105	C104100	Diabetes mellitus	
35385	C10FH00	Type 2 diabetes mellitus with neuropathic arthropathy	Feb-09
36633	C109K00	Hyperosmolar non-ketotic state in type 2 diabetes mellitus	Feb-09
37648	C109J11	Insulin treated non-insulin dependent diabetes mellitus	Feb-09
37806	C10FF00	Type 2 diabetes mellitus with peripheral angiopathy	Feb-09
39317	C106100	Diabetes mellitus	

40401	C109500	Non-insulin dependent diabetes mellitus with gangrene	Feb-09
40962	C109H00	Non-insulin dependent d m with neuropathic arthropathy	Feb-09
41389	C105100	Diabetes mellitus	
42762	C109612	Type 2 diabetes mellitus with retinopathy	Feb-09
43139	C102100	Diabetes mellitus	
43227	C10F311	Type II diabetes mellitus with multiple complications	Feb-09
43785	C109D00	Non-insulin dependent diabetes mellitus with hypoglyca coma	Feb-09
44779	C109E12	Type 2 diabetes mellitus with diabetic cataract	Feb-09
44982	C10FE00	Type 2 diabetes mellitus with diabetic cataract	Feb-09
45467	C109B00	Non-insulin dependent diabetes mellitus with polyneuropathy	Feb-09
45913	C109712	Type 2 diabetes mellitus - poor control	Feb-09
45919	C109212	Type 2 diabetes mellitus with neurological complications	Feb-09
46150	C109512	Type 2 diabetes mellitus with gangrene	Feb-09
46917	C10FD00	Type 2 diabetes mellitus with hypoglycaemic coma	Feb-09
47315	C10F711	Type II diabetes mellitus - poor control	Feb-09
47321	C10F100	Type 2 diabetes mellitus with ophthalmic complications	Feb-09
47409	C109B11	Type II diabetes mellitus with polyneuropathy	Feb-09
47816	C109H11	Type II diabetes mellitus with neuropathic arthropathy	Feb-09
47954	C10F900	Type 2 diabetes mellitus without complication	Feb-09
48192	C109E11	Type II diabetes mellitus with diabetic cataract	Feb-09
49074	C10F400	Type 2 diabetes mellitus with ulcer	Feb-09

49655	C10F611	Type II diabetes mellitus with retinopathy	Feb-09
49869	C109G12	Type 2 diabetes mellitus with arthropathy	Feb-09
50225	C109011	Type II diabetes mellitus with renal complications	Feb-09
50429	C109100	Non-insulin-dependent diabetes mellitus with ophthalm comps	Feb-09
50527	C10FB11	Type II diabetes mellitus with polyneuropathy	Feb-09
50813	C109A11	Type II diabetes mellitus with mononeuropathy	Feb-09
51756	C10FP00	Type 2 diabetes mellitus with ketoacidotic coma	Feb-09
52303	C109000	Non-insulin-dependent diabetes mellitus with renal comps	Feb-09
53392	C10F911	Type II diabetes mellitus without complication	Feb-09
54212	C109F00	Non-insulin-dependent d m with peripheral angiopath	Feb-09
54899	C109F11	Type II diabetes mellitus with peripheral angiopathy	Feb-09
55075	C109411	Type II diabetes mellitus with ulcer	Feb-09
55842	C109200	Non-insulin-dependent diabetes mellitus with neuro comps	Feb-09
56268	C109D11	Type II diabetes mellitus with hypoglycaemic coma	Feb-09
56803	C107400	NIDDM with peripheral circulatory disorder	Feb-09
57278	C10F011	Type II diabetes mellitus with renal complications	Feb-09
58604	C109611	Type II diabetes mellitus with retinopathy	Feb-09
59253	C10FG00	Type 2 diabetes mellitus with arthropathy	Feb-09
59365	C109C00	Non-insulin dependent diabetes mellitus with nephropathy	Feb-09
59725	C109111	Type II diabetes mellitus with ophthalmic complications	Feb-09
60699	C109F12	Type 2 diabetes mellitus with peripheral angiopathy	Feb-09
60796	C10FL11	Type II diabetes mellitus with persistent proteinuria	Feb-09
61071	C109D12	Type 2 diabetes mellitus with hypoglycaemic coma	Feb-09

62107	C109511	Type II diabetes mellitus with gangrene	Feb-09
62146	C109300	Non-insulin-dependent diabetes mellitus with multiple comps	Feb-09
62674	C10FA00	Type 2 diabetes mellitus with mononeuropathy	Feb-09
63357	C107100	Diabetes mellitus	
63690	C10FR00	Type 2 diabetes mellitus with gastroparesis	Feb-09
64571	C109C11	Type II diabetes mellitus with nephropathy	Feb-09
64668	C10FJ11	Insulin treated Type II diabetes mellitus	Feb-09
65267	C10F300	Type 2 diabetes mellitus with multiple complications	Feb-09
65704	C109412	Type 2 diabetes mellitus with ulcer	Feb-09
66965	C109H12	Type 2 diabetes mellitus with neuropathic arthropathy	Feb-09
67905	C109211	Type II diabetes mellitus with neurological complications	Feb-09
69278	C109E00	Non-insulin depend diabetes mellitus with diabetic cataract	Feb-09
70316	C109112	Type 2 diabetes mellitus with ophthalmic complications	Feb-09
72320	C109A00	Non-insulin dependent diabetes mellitus with mononeuropathy	Feb-09
83532	66Ao.00	Diabetes type 2 review	Feb-09
85991	C10FM11	Type II diabetes mellitus with persistent microalbuminuria	Feb-09
91646	C10F411	Type II diabetes mellitus with ulcer	Feb-09
93529	9OLK.00	DESMOND diabetes structured education programme completed	Feb-09
93530	9OLE.00	Attended DESMOND structured programme	Feb-09
93657	8Hj4.00	Referral to DESMOND diabetes structured education programme	Feb-09

93727	C10FE11	Type II diabetes mellitus with diabetic cataract	Feb-09
95093	8I83.00	Did not complete DESMOND diabetes structured educat program	Feb-09
95159	9NiD.00	Did not attend DESMOND diabetes structured education program	Feb-09
95351	C10FA11	Type II diabetes mellitus with mononeuropathy	Feb-09
98616	C10F211	Type II diabetes mellitus with neurological complications	Mar-10
98723	C10FD11	Type II diabetes mellitus with hypoglycaemic coma	Mar-10
100964	C10F111	Type II diabetes mellitus with ophthalmic complications	Nov-10
101801	66At100	Type II diabetic dietary review	Feb-11
102201	C10FC11	Type II diabetes mellitus with nephropathy	May-11
102611	66At111	Type 2 diabetic dietary review	Jul-11
103543	8IE9.00	Referral to DESMOND structured programme declined	Jan-12
103902	C10FG11	Type II diabetes mellitus with arthropathy	Mar-12
104323	C10F511	Type II diabetes mellitus with gangrene	Jun-12
104639	C10FF11	Type II diabetes mellitus with peripheral angiopathy	Aug-12
105784	C109912	Type 2 diabetes mellitus without complication	Feb-13
106061	C10FP11	Type II diabetes mellitus with ketoacidotic coma	May-13
106528	C10FN11	Type II diabetes mellitus with ketoacidosis	Aug-13
107331	66AH100	Conversion to insulin in secondary care	Dec-13
107508	66AH200	Conversion to insulin by diabetes specialist nurse	Dec-13
107701	C10FK11	Hyperosmolar non-ketotic state in type II diabetes mellitus	Feb-14
108005	C109312	Type 2 diabetes mellitus with multiple complications	May-14
85660	66An.00	Diabetes type 1 review	Feb-09
102704	66At000	Type I diabetic dietary review	Aug-11

104453	66At011	Type 1 diabetic dietary review	Jun-12
24490	C100000	Diabetes mellitus, juvenile type, no mention of complication	Feb-09
1038	C100011	Insulin dependent diabetes mellitus	Feb-09
53200	C101000	Diabetes mellitus, juvenile type, with ketoacidosis	Feb-09
40023	C102000	Diabetes mellitus, juvenile type, with hyperosmolar coma	Feb-09
42567	C103000	Diabetes mellitus, juvenile type, with ketoacidotic coma	Feb-09
93922	C104000	Diabetes mellitus, juvenile type, with renal manifestation	Feb-09
69748	C105000	Diabetes mellitus, juvenile type, + ophthalmic manifestation	Feb-09
67853	C106000	Diabetes mellitus, juvenile, + neurological manifestation	Feb-09
70448	C107000	Diabetes mellitus, juvenile +peripheral circulatory disorder	Feb-09
69124	C107300	IDDM with peripheral circulatory disorder	Feb-09
1647	C108.00	Insulin dependent diabetes mellitus	Feb-09
18505	C108.11	IDDM-Insulin dependent diabetes mellitus	Feb-09
17858	C108.12	Type 1 diabetes mellitus	Feb-09
24423	C108.13	Type I diabetes mellitus	Feb-09
46963	C108000	Insulin-dependent diabetes mellitus with renal complications	Feb-09
61344	C108011	Type I diabetes mellitus with renal complications	Feb-09
21983	C108012	Type 1 diabetes mellitus with renal complications	Feb-09
49276	C108100	Insulin-dependent diabetes mellitus with ophthalmic comps	Feb-09
102740	C108112	Type 1 diabetes mellitus with ophthalmic complications	Aug-11
52283	C108200	Insulin-dependent diabetes mellitus with neurological comps	Feb-09
49146	C108211	Type I diabetes mellitus with neurological complications	Feb-09
61829	C108212	Type 1 diabetes mellitus with neurological complications	Feb-09

52104	C108300	Insulin dependent diabetes mellitus with multiple complicatn	Feb-09
108007	C108311	Type I diabetes mellitus with multiple complications	May-14
26855	C108400	Unstable insulin dependent diabetes mellitus	Feb-09
60107	C108411	Unstable type I diabetes mellitus	Feb-09
97474	C108412	Unstable type 1 diabetes mellitus	Aug-09
44443	C108500	Insulin dependent diabetes mellitus with ulcer	Feb-09
51957	C108511	Type I diabetes mellitus with ulcer	Feb-09
68390	C108512	Type 1 diabetes mellitus with ulcer	Feb-09
60499	C108600	Insulin dependent diabetes mellitus with gangrene	Feb-09
6509	C108700	Insulin dependent diabetes mellitus with retinopathy	Feb-09
38161	C108711	Type I diabetes mellitus with retinopathy	Feb-09
41049	C108712	Type 1 diabetes mellitus with retinopathy	Feb-09
6791	C108800	Insulin dependent diabetes mellitus - poor control	Feb-09
46850	C108811	Type I diabetes mellitus - poor control	Feb-09
45914	C108812	Type 1 diabetes mellitus - poor control	Feb-09
63017	C108911	Type I diabetes mellitus maturity onset	Feb-09
97446	C108912	Type 1 diabetes mellitus maturity onset	Aug-09
56448	C108A00	Insulin-dependent diabetes without complication	Feb-09
95992	C108A11	Type I diabetes mellitus without complication	Feb-09
24694	C108B00	Insulin dependent diabetes mellitus with mononeuropathy	Feb-09
99231	C108B11	Type I diabetes mellitus with mononeuropathy	Apr-10
41716	C108C00	Insulin dependent diabetes mellitus with polyneuropathy	Feb-09
57621	C108D00	Insulin dependent diabetes mellitus with nephropathy	Feb-09
66872	C108D11	Type I diabetes mellitus with nephropathy	Feb-09
44440	C108E00	Insulin dependent diabetes mellitus with hypoglycaemic coma	Feb-09
42729	C108E11	Type I diabetes mellitus with hypoglycaemic coma	Feb-09

70766	C108E12	Type 1 diabetes mellitus with hypoglycaemic coma	Feb-09
44260	C108F00	Insulin dependent diabetes mellitus with diabetic cataract	Feb-09
17545	C108F11	Type I diabetes mellitus with diabetic cataract	Feb-09
64446	C108G00	Insulin dependent diab mell with peripheral angiopathy	Feb-09
65616	C108H00	Insulin dependent diabetes mellitus with arthropathy	Feb-09
62352	C108H11	Type I diabetes mellitus with arthropathy	Feb-09
39809	C108J00	Insulin dependent diab mell with neuropathic arthropathy	Feb-09
60208	C108J11	Type I diabetes mellitus with neuropathic arthropathy	Feb-09
18230	C108J12	Type 1 diabetes mellitus with neuropathic arthropathy	Feb-09
1549	C10E.00	Type 1 diabetes mellitus	Feb-09
12455	C10E.11	Type I diabetes mellitus	Feb-09
51261	C10E.12	Insulin dependent diabetes mellitus	Feb-09
47582	C10E000	Type 1 diabetes mellitus with renal complications	Feb-09
102946	C10E012	Insulin-dependent diabetes mellitus with renal complications	Sep-11
47649	C10E100	Type 1 diabetes mellitus with ophthalmic complications	Feb-09
99311	C10E111	Type I diabetes mellitus with ophthalmic complications	May-10
98071	C10E112	Insulin-dependent diabetes mellitus with ophthalmic comps	Nov-09
42831	C10E200	Type 1 diabetes mellitus with neurological complications	Feb-09
101735	C10E212	Insulin-dependent diabetes mellitus with neurological comps	Feb-11
47650	C10E300	Type 1 diabetes mellitus with multiple complications	Feb-09
91942	C10E311	Type I diabetes mellitus with multiple complications	Feb-09
45276	C10E312	Insulin dependent diabetes mellitus with multiple complicat	Feb-09
43921	C10E400	Unstable type 1 diabetes mellitus	Feb-09
49949	C10E411	Unstable type I diabetes mellitus	Feb-09

54600	C10E412	Unstable insulin dependent diabetes mellitus	Feb-09
18683	C10E500	Type 1 diabetes mellitus with ulcer	Feb-09
93878	C10E511	Type I diabetes mellitus with ulcer	Feb-09
98704	C10E512	Insulin dependent diabetes mellitus with ulcer	Mar-10
69993	C10E600	Type 1 diabetes mellitus with gangrene	Feb-09
102112	C10E611	Type I diabetes mellitus with gangrene	Apr-11
18387	C10E700	Type 1 diabetes mellitus with retinopathy	Feb-09
95343	C10E711	Type I diabetes mellitus with retinopathy	Feb-09
93875	C10E712	Insulin dependent diabetes mellitus with retinopathy	Feb-09
35288	C10E800	Type 1 diabetes mellitus - poor control	Feb-09
105337	C10E811	Type I diabetes mellitus - poor control	Dec-12
72702	C10E812	Insulin dependent diabetes mellitus - poor control	Feb-09
40682	C10E900	Type 1 diabetes mellitus maturity onset	Feb-09
96235	C10E911	Type I diabetes mellitus maturity onset	Apr-09
69676	C10EA00	Type 1 diabetes mellitus without complication	Feb-09
62613	C10EA11	Type I diabetes mellitus without complication	Feb-09
99719	C10EA12	Insulin-dependent diabetes without complication	Jun-10
68105	C10EB00	Type 1 diabetes mellitus with mononeuropathy	Feb-09
46301	C10EC00	Type 1 diabetes mellitus with polyneuropathy	Feb-09
91943	C10EC11	Type I diabetes mellitus with polyneuropathy	Feb-09
101311	C10EC12	Insulin dependent diabetes mellitus with polyneuropathy	Jan-11
10418	C10ED00	Type 1 diabetes mellitus with nephropathy	Feb-09
102163	C10ED12	Insulin dependent diabetes mellitus with nephropathy	Apr-11
39070	C10EE00	Type 1 diabetes mellitus with hypoglycaemic coma	Feb-09
99716	C10EE12	Insulin dependent diabetes mellitus with hypoglycaemic coma	Jun-10
49554	C10EF00	Type 1 diabetes mellitus with diabetic cataract	Feb-09

100770	C10EF12	Insulin dependent diabetes mellitus with diabetic cataract	Nov-10
93468	C10EG00	Type 1 diabetes mellitus with peripheral angiopathy	Feb-09
18642	C10EH00	Type 1 diabetes mellitus with arthropathy	Feb-09
54008	C10EJ00	Type 1 diabetes mellitus with neuropathic arthropathy	Feb-09
30323	C10EK00	Type 1 diabetes mellitus with persistent proteinuria	Feb-09
30294	C10EL00	Type 1 diabetes mellitus with persistent microalbuminuria	Feb-09
102620	C10EL11	Type I diabetes mellitus with persistent microalbuminuria	Jul-11
10692	C10EM00	Type 1 diabetes mellitus with ketoacidosis	Feb-09
62209	C10EM11	Type I diabetes mellitus with ketoacidosis	Feb-09
40837	C10EN00	Type 1 diabetes mellitus with ketoacidotic coma	Feb-09
66145	C10EN11	Type I diabetes mellitus with ketoacidotic coma	Feb-09
22871	C10EP00	Type 1 diabetes mellitus with exudative maculopathy	Feb-09
97894	C10EP11	Type I diabetes mellitus with exudative maculopathy	Oct-09
55239	C10EQ00	Type 1 diabetes mellitus with gastroparesis	Feb-09
108724	C10EQ11	Type I diabetes mellitus with gastroparesis	Oct-14

Prodcodes

**prodcode**	**Product name**	**bnfcode**	**Database build**	**Category**
479	Acarbose 50mg tablets	6010203	Feb-09	Acarbose
5174	Acarbose 100mg tablets	6010203	Feb-09	Acarbose
5621	Glucobay 50mg tablets (Bayer Plc)	6010203	Feb-09	Acarbose
9105	Glucobay 100mg tablets (Bayer Plc)	6010203	Feb-09	Acarbose
35022	Sitagliptin 100mg tablets	6010203	Feb-09	DPP-4 Inhibitor

35462	Januvia 100mg tablets (Merck Sharp & Dohme Ltd)	6010203	Feb-09	DPP-4 Inhibitor
37874	Vildagliptin 50mg / Metformin 850mg tablets	6010203	Feb-09	DPP-4 Inhibitor
37875	Vildagliptin 50mg tablets	6010200	Feb-09	DPP-4 Inhibitor
37902	Vildagliptin 50mg / Metformin 1g tablets	6010203	Feb-09	DPP-4 Inhibitor
38551	Eucreas 50mg/1000mg tablets (Novartis Pharmaceuticals UK Ltd)	6010203	Feb-09	DPP-4 Inhibitor
39149	Galvus 50mg tablets (Novartis Pharmaceuticals UK Ltd)	6010200	Apr-09	DPP-4 Inhibitor
39203	Eucreas 50mg/850mg tablets (Novartis Pharmaceuticals UK Ltd)	6010203	Apr-09	DPP-4 Inhibitor
41204	Saxagliptin 5mg tablets	6010203	Feb-10	DPP-4 Inhibitor
41431	Onglyza 5mg tablets (AstraZeneca UK Ltd)	6010203	Mar-10	DPP-4 Inhibitor
43619	Metformin 1g / Sitagliptin 50mg tablets	6010203	Oct-10	DPP-4 Inhibitor
43684	Janumet 50mg/1000mg tablets (Merck Sharp & Dohme Ltd)	6010203	Oct-10	DPP-4 Inhibitor
45775	Saxagliptin 2.5mg tablets	6010203	Aug-11	DPP-4 Inhibitor
45821	Onglyza 2.5mg tablets (Bristol-Myers Squibb Pharmaceuticals Ltd)	6010203	Aug-11	DPP-4 Inhibitor
46665	Linagliptin 5mg tablets	6010203	Jan-12	DPP-4 Inhibitor

46716	Trajenta 5mg tablets (Boehringer Ingelheim Ltd)	6010203	Jan-12	DPP-4 Inhibitor
48401	Sitagliptin 50mg tablets	6010203	Jan-13	DPP-4 Inhibitor
48533	Sitagliptin 25mg tablets	6010203	Jan-13	DPP-4 Inhibitor
50087	Januvia 50mg tablets (Merck Sharp & Dohme Ltd)	6010203	Jan-13	DPP-4 Inhibitor
50124	Januvia 25mg tablets (Merck Sharp & Dohme Ltd)	6010203	Jan-13	DPP-4 Inhibitor
50682	Jentadueto 2.5mg/1000mg tablets (Boehringer Ingelheim Ltd)	06010202/06010203	Jan-13	DPP-4 Inhibitor
54150	Jentadueto 2.5mg/850mg tablets (Boehringer Ingelheim Ltd)	06010202/06010203	Mar-13	DPP-4 Inhibitor
54891	Saxagliptin 2.5mg / Metformin 1g tablets	6010203	May-13	DPP-4 Inhibitor
54973	Saxagliptin 2.5mg / Metformin 850mg tablets	6010203	May-13	DPP-4 Inhibitor
56965	Komboglyze 2.5mg/1000mg tablets (AstraZeneca UK Ltd)	6010203	Sep-13	DPP-4 Inhibitor
58865	Komboglyze 2.5mg/850mg tablets (AstraZeneca UK Ltd)	6010203	Feb-14	DPP-4 Inhibitor
59177	Alogliptin 25mg tablets	6010200	Mar-14	DPP-4 Inhibitor
59385	Vipdomet 12.5mg/1000mg tablets (Takeda UK Ltd)	6010200	Apr-14	DPP-4 Inhibitor
59809	Alogliptin 6.25mg tablets	6010200	May-14	DPP-4 Inhibitor
60328	Alogliptin 12.5mg tablets	6010200	Jul-14	DPP-4 Inhibitor
60497	Alogliptin 12.5mg / Metformin 1g tablets	6010200	Jul-14	DPP-4 Inhibitor

60681	Vipidia 12.5mg tablets (Takeda UK Ltd)	6010200	Aug-14	DPP-4 Inhibitor
60682	Vipidia 25mg tablets (Takeda UK Ltd)	6010200	Aug-14	DPP-4 Inhibitor
54182	Dapagliflozin 10mg tablets	6010203	Mar-13	SGLT-2 inhibitor
54203	Forxiga 10mg tablets (Bristol-Myers Squibb Pharmaceuticals Ltd)	6010203	Mar-13	SGLT-2 inhibitor
54265	Dapagliflozin 5mg tablets	6010203	Mar-13	SGLT-2 inhibitor
54480	Forxiga 5mg tablets (AstraZeneca UK Ltd)	6010203	Apr-13	SGLT-2 inhibitor
60012	Dapagliflozin 5mg / Metformin 1g tablets	6010202	May-14	SGLT-2 inhibitor
60066	Invokana 100mg tablets (Janssen-Cilag Ltd)	6010203	May-14	SGLT-2 inhibitor
60073	Canagliflozin 100mg tablets	6010203	Jun-14	SGLT-2 inhibitor
60211	Canagliflozin 100mg tablets	6010203	Jun-14	SGLT-2 inhibitor
60379	Invokana 300mg tablets (Janssen-Cilag Ltd)	6010203	Jul-14	SGLT-2 inhibitor
60386	Canagliflozin 300mg tablets	6010203	Jul-14	SGLT-2 inhibitor
60430	Invokana 100mg tablets (Janssen-Cilag Ltd)	6010203	Jul-14	SGLT-2 inhibitor
60643	Xigduo 5mg/1000mg tablets (AstraZeneca UK Ltd)	6010202	Jul-14	SGLT-2 inhibitor
35144	Byetta 5micrograms/0.02ml solution for injection 1.2ml pre-filled disposable devices (AstraZeneca UK Ltd)	6010203	Feb-09	GLP1 agonist

35149	Exenatide 10micrograms/0.04ml solution for injection 2.4ml pre-filled disposable devices	6010203	Feb-09	GLP1 agonist
35150	Byetta 10micrograms/0.04ml solution for injection 2.4ml pre-filled disposable devices (AstraZeneca UK Ltd)	6010203	Feb-09	GLP1 agonist
35251	Exenatide 5micrograms/0.02ml solution for injection 1.2ml pre-filled disposable devices	6010203	Feb-09	GLP1 agonist
40642	Victoza 6mg/ml solution for injection 3ml pre-filled pen (Novo Nordisk Ltd)	6010203	Oct-09	GLP1 agonist
40693	Liraglutide 6mg/ml solution for injection 3ml pre-filled disposable devices	6010203	Nov-09	GLP1 agonist
46458	Exenatide 2mg powder and solvent for suspension for injection vials	6010203	Nov-11	GLP1 agonist
46469	Bydureon 2mg powder and solvent for suspension for injection vials (Bristol-Myers Squibb Pharmaceuticals Ltd)	6010203	Nov-11	GLP1 agonist
55413	Lixisenatide 20micrograms/0.2ml solution for injection 3ml pre-filled disposable devices	6010203	May-13	GLP1 agonist

55459	Lixisenatide 10micrograms/0.2ml solution for injection 3ml pre-filled disposable devices	6010203	Jun-13	GLP1 agonist
55723	Lixisenatide 10micrograms/0.2ml solution for injection 3ml pre-filled disposable devices and Lixisenatide 20micrograms/0.2ml solution for injection 3ml pre-filled disposable devices	6010203	Jun-13	GLP1 agonist
55728	Lyxumia 10micrograms/0.2ml solution for injection 3ml pre-filled pen (Sanofi)	6010203	Jun-13	GLP1 agonist
55729	Lyxumia 20micrograms/0.2ml solution for injection 3ml pre-filled pen (Sanofi)	6010203	Jun-13	GLP1 agonist
55767	Lyxumia 10micrograms/0.2ml solution for injection 3ml pre-filled pen and Lyxumia 20micrograms/0.2ml solution for injection 3ml pre-filled pen (Sanofi)	6010203	Jun-13	GLP1 agonist
321	INSULIN HUMAN ACTRAPID (NEUTRAL) 40 I/U INJ	0	Feb-09	Insulin

322	Humalog 100units/ml solution for injection 1.5ml cartridges (Eli Lilly and Company Ltd)	0	Feb-09	Insulin
1587	Monotard 100units/ml suspension for injection 10ml vials (Novo Nordisk Ltd)	0	Feb-09	Insulin
1588	Actrapid 100iu/ml Injection (Novo Nordisk Ltd)	06010101/06010300	Feb-09	Insulin
1589	U100 Insulin syringe 0.5ml	6010103	Feb-09	Insulin
1591	U100 Insulin syringe 1ml	6010103	Feb-09	Insulin
1592	Actrapid penfill 100 100iu/ml Penfill (Novo Nordisk Ltd)	06010101/06010300	Feb-09	Insulin
1593	Insulatard penfill 100 100iu/ml Penfill (Novo Nordisk Ltd)	6010102	Feb-09	Insulin
1594	Actrapid NovoLet 100units/ml solution for injection (Novo Nordisk Ltd)	0	Feb-09	Insulin
1595	Insulatard NovoLet 100units/ml suspension for injection (Novo Nordisk Ltd)	6010102	Feb-09	Insulin
1643	INSULIN NOVO MONOTARD MC 100 I/U INJ	0	Feb-09	Insulin
1645	INSULIN NOVO ACTRAPID MC 100 I/U INJ	0	Feb-09	Insulin

1649	Human actraphane 100iu/ml Injection (Novo Nordisk Ltd)	6010102	Feb-09	Insulin
1751	Hypodermic U100 insulin syringe glass reusable 1ml	0	Feb-09	Insulin
1805	Mixtard 30/70 100unit/ml Injection (Novo Nordisk Ltd)	6010102	Feb-09	Insulin
1806	Penmix 30/70 100iu/ml Penfill (Novo Nordisk Ltd)	6010102	Feb-09	Insulin
1839	INSULIN HUMULIN I (ISOPHANE) 100 I/U INJ	0	Feb-09	Insulin
1840	Humulin s 100unit/ml Injection (Eli Lilly and Company Ltd)	06010101/06010300	Feb-09	Insulin
1842	Pork velosulin 100unit/ml Injection (Novo Nordisk Ltd)	06010101/06010300	Feb-09	Insulin
1843	Pork Insulatard 100units/ml suspension for injection 10ml vials (Novo Nordisk Ltd)	0	Feb-09	Insulin
1844	Ultratard 100units/ml suspension for injection 10ml vials (Novo Nordisk Ltd)	0	Feb-09	Insulin
1886	Insulatard 100iu/ml GE injection (Novo Nordisk Ltd)	6010102	Feb-09	Insulin
2220	Penmix 20/80 Pen (Novo Nordisk Ltd)	6010102	Feb-09	Insulin

2221	Mixtard 30 NovoLet 100units/ml suspension for injection (Novo Nordisk Ltd)	0	Feb-09	Insulin
2321	Insulin 0.5ml disposable syringe	59010200	Feb-09	Insulin
2373	INSULIN HUMAN VELOSULIN 100 I/U INJ	0	Feb-09	Insulin
2454	Mixtard 30 penfill 100 100iu/ml Penfill (Novo Nordisk Ltd)	6010102	Feb-09	Insulin
2455	Mixtard 20 NovoLet 100units/ml suspension for injection (Novo Nordisk Ltd)	0	Feb-09	Insulin
2456	Mixtard 10 NovoLet 100units/ml suspension for injection (Novo Nordisk Ltd)	0	Feb-09	Insulin
2459	Pork Mixtard 30 100units/ml suspension for injection 10ml vials (Novo Nordisk Ltd)	0	Feb-09	Insulin
2808	INSULIN LENTARD INJ	0	Feb-09	Insulin
2812	Mixtard 40 NovoLet 100units/ml suspension for injection (Novo Nordisk Ltd)	0	Feb-09	Insulin
2929	Mixtard 30 100iu/ml GE injection (Novo Nordisk Ltd)	6010102	Feb-09	Insulin
3076	NovoFine hypodermic insulin needles for pre-filled / reusable pen injectors	71190600	Feb-09	Insulin

	screw on 6mm/30gauge (Novo Nordisk Ltd)			
3396	Penmix 10/90 Penfill (Novo Nordisk Ltd)	6010102	Feb-09	Insulin
3439	Penmix 10/90 Pen (Novo Nordisk Ltd)	6010102	Feb-09	Insulin
3550	Mixtard 40 penfill 100 100iu/ml Penfill (Novo Nordisk Ltd)	6010102	Feb-09	Insulin
3551	Mixtard 20 penfill 100 100iu/ml Penfill (Novo Nordisk Ltd)	6010102	Feb-09	Insulin
4093	Humulin M2 100units/ml suspension for injection 3ml cartridges (Eli Lilly and Company Ltd)	0	Feb-09	Insulin
4129	Insulin soluble porcine 100units/ml solution for injection 1.5ml cartridges	0	Feb-09	Insulin
4163	Rapitard MC 100unit/ml Injection (Novo Nordisk Ltd)	6010102	Feb-09	Insulin
4198	Humulin m3 100unit/ml M3 injection (Eli Lilly and Company Ltd)	6010102	Feb-09	Insulin
4199	Humulin m1 100unit/ml M1 injection (Eli Lilly and Company Ltd)	6010102	Feb-09	Insulin
4247	Insulin isophane porcine 100units/ml suspension for injection 1.5ml cartridges	0	Feb-09	Insulin

4248	INSULIN NOVO ULTRATARD MC 100 I/U INJ	0	Feb-09	Insulin
4706	Velosulin 100units/ml solution for injection 10ml vials (Novo Nordisk Ltd)	0	Feb-09	Insulin
4715	Humalog mix 25 25/75 100units/ml Injection (Eli Lilly and Company Ltd)	6010102	Feb-09	Insulin
4760	Humulin i 100unit/ml Injection (Eli Lilly and Company Ltd)	6010102	Feb-09	Insulin
4784	Lentard mc 100unit/ml Injection (Novo Nordisk Ltd)	6010102	Feb-09	Insulin
4790	Mixtard 50 penfill 100 100iu/ml Penfill (Novo Nordisk Ltd)	6010102	Feb-09	Insulin
4896	Monoject hypodermic U100 insulin syringe sterile single use / single patient use 1ml with 12mm needle 0.36mm/28gauge (Covidien (UK) Commercial Ltd)	71190300	Feb-09	Insulin
5015	NovoFine hypodermic insulin needles for pre-filled / reusable pen injectors screw on 8mm/30gauge (Novo Nordisk Ltd)	71190600	Feb-09	Insulin
5021	NovoRapid Penfill 100units/ml solution for	6010101	Feb-09	Insulin

	injection 3ml cartridges (Novo Nordisk Ltd)			
5022	NovoFine hypodermic insulin needles for pre-filled / reusable pen injectors screw on 12mm/28gauge (Novo Nordisk Ltd)	71190600	Feb-09	Insulin
5059	NovoPen 3 Classic hypodermic insulin injection pen reusable for 3ml cartridge 1 unit dial up / range 2-70 units (Novo Nordisk Ltd)	0	Feb-09	Insulin
5121	BD Micro-Fine + hypodermic insulin needles for pre-filled / reusable pen injectors screw on 12.7mm/29gauge (Becton, Dickinson UK Ltd)	71190600	Feb-09	Insulin
5142	BD Micro-Fine + hypodermic insulin needles for pre-filled / reusable pen injectors screw on 8mm/31gauge (Becton, Dickinson UK Ltd)	71190600	Feb-09	Insulin
5164	NovoPen 3 Demi hypodermic insulin injection pen reusable for 3ml cartridge 0.5 unit dial up /	71190900	Feb-09	Insulin

	range 1-35 units (Novo Nordisk Ltd)			
5214	Insulin lispro 100units/ml solution for injection 1.5ml cartridges	0	Feb-09	Insulin
5250	Insulin biphasic lispro human prb 25:75; 100 units/ml Injection	6010102	Feb-09	Insulin
5255	Mixtard 10 penfill 100 100iu/ml Penfill (Novo Nordisk Ltd)	6010102	Feb-09	Insulin
5267	BD Micro-Fine + hypodermic U100 insulin syringe sterile single use / single patient use 0.5ml with 8mm needle 0.3mm/30gauge (Becton, Dickinson UK Ltd)	71190300	Feb-09	Insulin
5345	Monoject hypodermic U100 insulin syringe sterile single use / single patient use 0.5ml with 12mm needle 0.36mm/28gauge (Covidien (UK) Commercial Ltd)	71190300	Feb-09	Insulin
5421	NovoFine hypodermic insulin needles for pre-filled / reusable pen injectors screw on 6mm/31gauge (Novo Nordisk Ltd)	71190600	Feb-09	Insulin

5501	Insuman basal 100iu/ml Injection (Aventis Pharma)	6010102	Feb-09	Insulin
5557	NovoPen 3 Fun hypodermic insulin injection pen reusable for 3ml cartridge 1 unit dial up / range 2-70 units Blue (Novo Nordisk Ltd)	0	Feb-09	Insulin
5620	U100 Insulin syringe 0.3ml	6010103	Feb-09	Insulin
5634	Hypodermic U100 insulin syringe sterile single use / single patient use 1ml with 12mm needle 0.36mm/28gauge	71190300	Feb-09	Insulin
5649	Autopen hypodermic insulin injection pen reusable for 1.5ml cartridge 1 unit dial up / range 1-16 units (Owen Mumford Ltd)	0	Feb-09	Insulin
5742	Unifine Pentips hypodermic insulin needles for pre-filled / reusable pen injectors screw on 8mm/30gauge (Owen Mumford Ltd)	71190600	Feb-09	Insulin
5769	Hypodermic U100 insulin syringe sterile single use / single patient use 0.5ml with 12mm needle 0.4mm/27gauge	71190300	Feb-09	Insulin

5789	Hypodermic U100 insulin syringe sterile single use / single patient use 0.5ml with 8mm needle 0.3mm/30gauge	71190300	Feb-09	Insulin
5845	Mixtard 30 InnoLet 100units/ml suspension for injection 3ml pre-filled pen (Novo Nordisk Ltd)	6010151	Feb-09	Insulin
5850	Mylife Clickfine hypodermic insulin needles for pre-filled / reusable pen injectors snap on 6mm/31gauge (Ypsomed Ltd)	71190700	Feb-09	Insulin
5873	BD Micro-Fine + hypodermic insulin needles for pre-filled / reusable pen injectors screw on 5mm/31gauge (Becton, Dickinson UK Ltd)	71190600	Feb-09	Insulin
5891	Insulatard FlexPen 100units/ml suspension for injection (Novo Nordisk Ltd)	6010102	Feb-09	Insulin
5892	NovoRapid FlexPen 100units/ml solution for injection 3ml pre-filled pen (Novo Nordisk Ltd)	6010101	Feb-09	Insulin
5933	Mixtard 50 NovoLet 100units/ml suspension for injection (Novo Nordisk Ltd)	0	Feb-09	Insulin

5953	Insulin glargine 100iu/ml Injection	6010102	Feb-09	Insulin
5962	Humapen inj device Injection device (Eli Lilly and Company Ltd)	6010103	Feb-09	Insulin
5966	Mylife Clickfine hypodermic insulin needles for pre-filled / reusable pen injectors snap on 8mm/31gauge (Ypsomed Ltd)	71190700	Feb-09	Insulin
5967	Hypodermic U100 insulin syringe sterile single use / single patient use 0.5ml with not less than 12mm needle 0.33mm/29gauge	71190300	Feb-09	Insulin
6009	Myjector hypodermic U100 insulin syringe sterile single use / single patient use 0.5ml with 12mm needle 0.4mm/27gauge (Terumo UK Ltd)	71190300	Feb-09	Insulin
6057	Lantus 100iu/ml Injection (Aventis Pharma)	6010102	Feb-09	Insulin
6060	Unifine Pentips hypodermic insulin needles for pre-filled / reusable pen injectors screw on 6mm/30gauge (Owen Mumford Ltd)	71190600	Feb-09	Insulin

6061	Novomix 30 30/70 100units/ml Injection (Novo Nordisk Ltd)	6010102	Feb-09	Insulin
6091	NovoPen 3 Fun hypodermic insulin injection pen reusable for 3ml cartridge 1 unit dial up / range 2-70 units Red (Novo Nordisk Ltd)	0	Feb-09	Insulin
6138	HumaPen Ergo hypodermic insulin injection pen reusable for 3ml cartridge 1 unit dial up / range 1-60 units Burgundy (Eli Lilly and Company Ltd)	0	Feb-09	Insulin
6209	NovoRapid 100units/ml solution for injection 10ml vials (Novo Nordisk Ltd)	6010101	Feb-09	Insulin
6228	BD Micro-Fine + hypodermic U100 insulin syringe sterile single use / single patient use 0.3ml with 8mm needle 0.3mm/30gauge (Becton, Dickinson UK Ltd)	71190300	Feb-09	Insulin
6233	Mylife Clickfine hypodermic insulin needles for pre-filled / reusable pen injectors snap on 10mm/29gauge (Ypsomed Ltd)	71190700	Feb-09	Insulin

6378	Hypodermic U100 insulin syringe sterile single use / single patient use 0.3ml with 8mm needle 0.3mm/30gauge	71190300	Feb-09	Insulin
6447	Insulin aspart human pyr 100 iu/ml Injection	6010101	Feb-09	Insulin
6470	Autopen 24 hypodermic insulin injection pen reusable for 3ml cartridge 2 unit dial up / range 2-42 units (Owen Mumford Ltd)	71190900	Feb-09	Insulin
6554	Autopen 24 hypodermic insulin injection pen reusable for 3ml cartridge 1 unit dial up / range 1-21 units (Owen Mumford Ltd)	71190900	Feb-09	Insulin
6724	U100 Insulin syringe 0.3ml	6010103	Feb-09	Insulin
6730	Hypodermic U100 insulin syringe sterile single use / single patient use 1ml with not less than 12mm needle 0.33mm/29gauge	71190300	Feb-09	Insulin
6753	Novopen Junior hypodermic insulin injection pen reusable for 3ml cartridge 0.5 unit dial up / range 1-35 units Green (Novo Nordisk Ltd)	71190900	Feb-09	Insulin

6781	Autopen Classic hypodermic insulin injection pen reusable for 3ml cartridge 2 unit dial up / range 2-42 units (Owen Mumford Ltd)	71190900	Feb-09	Insulin
6831	HumaPen Ergo hypodermic insulin injection pen reusable for 3ml cartridge 1 unit dial up / range 1-60 units Teal (Eli Lilly and Company Ltd)	0	Feb-09	Insulin
6958	Levemir FlexPen 100units/ml solution for injection 3ml pre-filled pen (Novo Nordisk Ltd)	6010102	Feb-09	Insulin
6965	Levemir Penfill 100units/ml solution for injection 3ml cartridges (Novo Nordisk Ltd)	6010102	Feb-09	Insulin
6981	Novopen Junior hypodermic insulin injection pen reusable for 3ml cartridge 0.5 unit dial up / range 1-35 units Yellow (Novo Nordisk Ltd)	71190900	Feb-09	Insulin
6991	BD Micro-Fine + hypodermic U100 insulin syringe sterile single use / single patient use 0.5ml with 12.7mm needle	71190300	Feb-09	Insulin

	0.33mm/29gauge (Becton, Dickinson UK Ltd)			
7062	Myjector hypodermic U100 insulin syringe sterile single use / single patient use 1ml with 12mm needle 0.4mm/27gauge (Terumo UK Ltd)	71190300	Feb-09	Insulin
7075	Optipen Pro 1 hypodermic insulin injection pen reusable for 3ml cartridge 1 unit dial up / range 1-60 units (Sanofi)	71190900	Feb-09	Insulin
7127	BD Micro-Fine + hypodermic U100 insulin syringe sterile single use / single patient use 1ml with 8mm needle 0.3mm/30gauge (Becton, Dickinson UK Ltd)	71190300	Feb-09	Insulin
7164	Unifine Pentips hypodermic insulin needles for pre-filled / reusable pen injectors screw on 6mm/31gauge (Owen Mumford Ltd)	71190600	Feb-09	Insulin
7203	Unifine Pentips hypodermic insulin needles for pre-filled / reusable pen injectors screw on 8mm/31gauge (Owen Mumford Ltd)	71190600	Feb-09	Insulin

7228	NovoMix 30 FlexPen 100units/ml suspension for injection 3ml pre-filled pen (Novo Nordisk Ltd)	6010151	Feb-09	Insulin
7231	Mixtard 30 Penfill 100units/ml suspension for injection 3ml cartridges (Novo Nordisk Ltd)	6010151	Feb-09	Insulin
7237	Lantus 100units/ml solution for injection 3ml pre-filled OptiSet pen (Sanofi)	6010102	Feb-09	Insulin
7266	Lantus 100units/ml solution for injection 3ml cartridges (Sanofi)	6010102	Feb-09	Insulin
7267	NovoMix 30 Penfill 100units/ml suspension for injection 3ml cartridges (Novo Nordisk Ltd)	6010151	Feb-09	Insulin
7300	Mixtard 30 100units/ml suspension for injection 10ml vials (Novo Nordisk Ltd)	6010151	Feb-09	Insulin
7318	Humalog 100units/ml solution for injection 3ml cartridges (Eli Lilly and Company Ltd)	6010101	Feb-09	Insulin
7319	Mixtard 20 Penfill 100units/ml suspension for injection 3ml cartridges (Novo Nordisk Ltd)	0	Feb-09	Insulin

7349	Actrapid 100units/ml solution for injection 10ml vials (Novo Nordisk Ltd)	6010101	Feb-09	Insulin
7350	Insulin isophane porcine 100units/ml suspension for injection 10ml vials	6010102	Feb-09	Insulin
7393	Insulin glargine 100units/ml solution for injection 3ml cartridges	6010102	Feb-09	Insulin
7400	Insulin glargine 100units/ml solution for injection 3ml pre-filled disposable devices	6010102	Feb-09	Insulin
7402	Lantus 100units/ml solution for injection 10ml vials (Sanofi)	6010102	Feb-09	Insulin
7412	U100 Insulin syringe 0.5ml	6010103	Feb-09	Insulin
7537	Humulin Zn 100units/ml suspension for injection 10ml vials (Eli Lilly and Company Ltd)	0	Feb-09	Insulin
7757	INSULIN NEULENTE (ZINC SUSP)(PURIFIED) 100 I/U INJ	0	Feb-09	Insulin
7763	INSULIN NEUPHANE (ISOPHANE)(PURIFIED) 100 I/U INJ	0	Feb-09	Insulin
7764	INSULIN NEUSULIN (NEUTRAL)(PURIFIED) 100 I/U INJ	0	Feb-09	Insulin
7765	INSULIN NEUTRAL (HUMAN) 100 I/U INJ	0	Feb-09	Insulin

7771	Human protaphane penfill 100 100unit/ml Penfill (Novo Nordisk Ltd)	6010102	Feb-09	Insulin
7772	Human protaphane 100unit/ml Injection (Novo Nordisk Ltd)	6010102	Feb-09	Insulin
7783	INSULIN ISOPHANE (HUMAN) 100 I/U INJ	0	Feb-09	Insulin
7793	HumaJect M3 Pen 100units/ml suspension for injection (Eli Lilly and Company Ltd)	0	Feb-09	Insulin
7861	INSULIN HUMULIN S (NEUTRAL) CARTRIDGE 100 I/U	0	Feb-09	Insulin
7959	INSULIN MIXTARD 30/70 40 I/U INJ	0	Feb-09	Insulin
8118	Humaject i 100iu/ml Pen (Eli Lilly and Company Ltd)	6010102	Feb-09	Insulin
8203	Penmix 50/50 100iu/ml Penfill (Novo Nordisk Ltd)	6010102	Feb-09	Insulin
8322	Insulin zinc suspension mixed human pyr 100unit/ml Injection	6010102	Feb-09	Insulin
8354	INSULIN ISOPHANE 70%/NEUTRAL 30% 100 I/U INJ	0	Feb-09	Insulin
8376	INSULIN ISOPHANE 100 I/U	0	Feb-09	Insulin
8483	MONOJECT INSULIN NEEDLES	0	Feb-09	Insulin

8646	INSULIN ZINC CRYSTALLINE susp 100 I/U INJ	0	Feb-09	Insulin
8838	INSULIN SEMITARD 40 I/U INJ	0	Feb-09	Insulin
8839	INSULIN SEMITARD 100 I/U INJ	0	Feb-09	Insulin
8841	Humulin M5 100units/ml suspension for injection 10ml vials (Eli Lilly and Company Ltd)	0	Feb-09	Insulin
8895	Initard 50/50 100unit/ml Injection (Novo Nordisk Ltd)	6010102	Feb-09	Insulin
9079	INSULIN SOLUBLE 100 I/U INJ	0	Feb-09	Insulin
9108	TOLBUTAMIDE 250 MG TAB	0	Feb-09	Insulin
9341	Insulin biphasic isophane human prb 30:70; 100 units/ml Injection	6010102	Feb-09	Insulin
9363	U100 Insulin syringe 0.5ml	6010103	Feb-09	Insulin
9376	Insulin zinc suspension crystalline human pyr 100unit/ml long acting Injection	6010102	Feb-09	Insulin
9503	Hypurin Bovine Protamine Zinc 100units/ml suspension for injection 10ml vials (Wockhardt UK Ltd)	6010102	Feb-09	Insulin
9521	Pork Actrapid 100units/ml solution for injection 10ml vials (Novo Nordisk Ltd)	6010101	Feb-09	Insulin

9565	HumaJect S Pen 100units/ml solution for injection (Eli Lilly and Company Ltd)	0	Feb-09	Insulin
9578	Unifine Pentips hypodermic insulin needles for pre-filled / reusable pen injectors screw on 12mm/29gauge (Owen Mumford Ltd)	71190600	Feb-09	Insulin
9618	Hypurin Porcine 30/70 Mix 100units/ml suspension for injection 1.5ml cartridges (C P Pharmaceuticals Ltd)	0	Feb-09	Insulin
9619	Bd Ultra Pen 1.5ml Insulin pen (Becton, Dickinson UK Ltd)	6010103	Feb-09	Insulin
9702	BD Micro-Fine + hypodermic U100 insulin syringe sterile single use / single patient use 1ml with 12.7mm needle 0.33mm/29gauge (Becton, Dickinson UK Ltd)	71190300	Feb-09	Insulin
9737	Insulatard innolet 100iu/ml Injection (Novo Nordisk Ltd)	6010102	Feb-09	Insulin
9834	Mylife Clickfine hypodermic insulin needles for pre-filled / reusable pen injectors snap on 12mm/29gauge (Ypsomed Ltd)	71190700	Feb-09	Insulin

10001	Humalog Mix50 Pen 100units/ml suspension for injection 3ml pre-filled pen (Eli Lilly and Company Ltd)	6010102	Feb-09	Insulin
10067	Insulin biphasic aspart human pyr 30:70; 100 units/ml Injection	6010102	Feb-09	Insulin
10133	U100 Insulin syringe 1ml	6010103	Feb-09	Insulin
10145	Humapen luxura insulin pen 3ml/1-60 units Insulin pen 3ml/1-60 units (Eli Lilly and Company Ltd)	6010103	Feb-09	Insulin
10175	Insulin isophane human 100units/ml suspension for injection 1.5ml cartridges	0	Feb-09	Insulin
10184	Insulin detemir 100 iu/ml Solution for injection	6010102	Feb-09	Insulin
10207	Insulin isophane human 100units/ml suspension for injection 3ml cartridges	6010102	Feb-09	Insulin
10208	Insulatard InnoLet 100units/ml suspension for injection 3ml pre-filled pen (Novo Nordisk Ltd)	6010102	Feb-09	Insulin
10225	Lantus 100units/ml solution for injection 3ml OptiClik cartridges (Sanofi)	6010102	Feb-09	Insulin
10229	Humulin I Pen 100units/ml suspension for injection 3ml	6010102	Feb-09	Insulin

	pre-filled pen (Eli Lilly and Company Ltd)			
10242	Hypodermic insulin needles for pre-filled / reusable pen injectors screw on 6mm/31gauge	71190600	Feb-09	Insulin
10243	Humalog Mix25 100units/ml suspension for injection 3ml cartridges (Eli Lilly and Company Ltd)	6010102	Feb-09	Insulin
10244	Mixtard 40 Penfill 100units/ml suspension for injection 3ml cartridges (Novo Nordisk Ltd)	0	Feb-09	Insulin
10245	Mixtard 10 Penfill 100units/ml suspension for injection 3ml cartridges (Novo Nordisk Ltd)	0	Feb-09	Insulin
10258	Hypodermic insulin needles for pre-filled / reusable pen injectors screw on 8mm/31gauge	71190600	Feb-09	Insulin
10259	Insulin glargine 100units/ml solution for injection 10ml vials	6010102	Feb-09	Insulin
10264	Humalog Pen 100units/ml solution for injection 3ml pre-filled pen (Eli Lilly and Company Ltd)	6010101	Feb-09	Insulin

10277	Humulin M3 100units/ml suspension for injection 3ml cartridges (Eli Lilly and Company Ltd)	6010151	Feb-09	Insulin
10484	Penmix 20/80 Penfill (Novo Nordisk Ltd)	6010102	Feb-09	Insulin
10545	INSULIN HUMULIN M4 CARTRIDGE 100 I/U	0	Feb-09	Insulin
10546	INSULIN HUMULIN M4 100 I/U INJ	0	Feb-09	Insulin
10547	Humulin Lente 100units/ml suspension for injection 10ml vials (Eli Lilly and Company Ltd)	0	Feb-09	Insulin
10566	INSULIN HUMULIN M CARTRIDGE 100 I/U	0	Feb-09	Insulin
10572	Insulin soluble bovine 100unit/ml Injection	06010101/06010300	Feb-09	Insulin
10691	INSULIN ISOPHANE (NPH) 100 I/U INJ	0	Feb-09	Insulin
10887	Penmix 40/60 100iu/ml Penfill (Novo Nordisk Ltd)	6010102	Feb-09	Insulin
10910	Humaject m2 100iu/ml M2 pen (Eli Lilly and Company Ltd)	6010102	Feb-09	Insulin
10915	Humaject m1 100iu/ml M1 pen (Eli Lilly and Company Ltd)	6010102	Feb-09	Insulin

11055	Insulin biphasic isophane human pyr 20:80; 100 units/ml Injection	6010102	Feb-09	Insulin
11056	Insulin biphasic isophane human pyr 30:70; 100 units/ml Injection	6010102	Feb-09	Insulin
11080	Insulin isophane human prb 100iu/ml Injection	6010102	Feb-09	Insulin
11086	B-d u-100 0.5ml Insulin syringe (Becton, Dickinson UK Ltd)	6010103	Feb-09	Insulin
11107	Humulin m4 100unit/ml M4 injection (Eli Lilly and Company Ltd)	6010102	Feb-09	Insulin
11245	B-d u-100 0.3ml Insulin syringe (Becton, Dickinson UK Ltd)	6010103	Feb-09	Insulin
11271	B-d u-100 1ml Insulin syringe (Becton, Dickinson UK Ltd)	6010103	Feb-09	Insulin
11337	NovoRapid Novolet 100units/ml solution for injection (Novo Nordisk Ltd)	0	Feb-09	Insulin
11345	Bd Ultra Pen 3ml Insulin pen (Becton, Dickinson UK Ltd)	6010103	Feb-09	Insulin
11346	B-d u-100 0.3ml Insulin syringe (Becton, Dickinson UK Ltd)	6010103	Feb-09	Insulin
11408	Autopen Classic hypodermic insulin injection pen	71190900	Feb-09	Insulin

	reusable for 3ml cartridge 1 unit dial up / range 1-21 units (Owen Mumford Ltd)			
11521	Hypodermic U100 insulin syringe sterile single use / single patient use 1ml with 12mm needle 0.4mm/27gauge	71190300	Feb-09	Insulin
11878	Hypodermic U100 insulin syringe sterile single use / single patient use 1ml with 8mm needle 0.3mm/30gauge	71190300	Feb-09	Insulin
12035	Insulin zinc mixed bovine 100units/ml suspension for injection 10ml vials	6010102	Feb-09	Insulin
12060	INSULIN QUICKSOL (SOLUBLE NEUTRAL) 100 I/U INJ	0	Feb-09	Insulin
12244	INSULIN ZINC BOVINE susp 100 I/U INJ	0	Feb-09	Insulin
12297	Hypurin bovine neutral 100unit/ml Injection (C P Pharmaceuticals Ltd)	06010101/06010300	Feb-09	Insulin
12299	Semitard mc 100unit/ml Injection (Novo Nordisk Ltd)	6010102	Feb-09	Insulin
12300	SYRINGE INSULIN (BS1619/1) 2ML	0	Feb-09	Insulin
12455	Rastinon 500mg Tablet (Hoechst Marion Roussel)	6010201	Feb-09	Insulin

12638	Insulin soluble human pyr 100unit/ml Injection	06010101/06010300	Feb-09	Insulin
12654	Insulin soluble human prb 100unit/ml Injection	06010101/06010300	Feb-09	Insulin
12818	Human Mixtard 50 100units/ml suspension for injection 10ml vials (Novo Nordisk Ltd)	0	Feb-09	Insulin
12840	B-d u-100 0.5ml Insulin syringe (Becton, Dickinson UK Ltd)	6010103	Feb-09	Insulin
12892	Clinipak u100 single use ins syr+12mm need28g 0.5ml [rand] 0.5ml Insulin syringe with 28gauge needle 12mm (Rand Rocket Ltd)	6010103	Feb-09	Insulin
13009	Hypodermic U100 insulin syringe sterile single use / single patient use 0.5ml with 12mm needle 0.36mm/28gauge	71190300	Feb-09	Insulin
13036	Unifine hypodermic U100 insulin syringe sterile single use / single patient use 0.5ml with 12mm needle 0.33mm/29gauge (Owen Mumford Ltd)	71190300	Feb-09	Insulin
13096	Autopen hypodermic insulin injection pen reusable for 1.5ml cartridge 2 unit dial	0	Feb-09	Insulin

	up / range 2-32 units (Owen Mumford Ltd)			
13108	Autopen Special Edition hypodermic insulin injection pen reusable for 3ml cartridge 1 unit dial up / range 1-21 units (Owen Mumford Ltd)	0	Feb-09	Insulin
13274	Clinipak u100 single use ins syr+12mm need28g 1ml [rand] 1ml Insulin syringe with 28gauge needle 12mm (Rand Rocket Ltd)	6010103	Feb-09	Insulin
13277	Mixtard 50 Penfill 100units/ml suspension for injection 3ml cartridges (Novo Nordisk Ltd)	0	Feb-09	Insulin
13331	Euglucon 5mg tablets (Sanofi)	6010201	Feb-09	Insulin
13416	Insulin biphasic 100 units/ml Injection	6010102	Feb-09	Insulin
13474	U100 Insulin syringe Sp.36 [2A] 1ml	6010103	Feb-09	Insulin
13516	Hypurin bovine isophane 100unit/ml Injection (C P Pharmaceuticals Ltd)	6010102	Feb-09	Insulin
13550	INSULIN BP 100 I/U	0	Feb-09	Insulin
13622	Hypurin porcine neutral 100unit/ml Injection (C P Pharmaceuticals Ltd)	06010101/06010300	Feb-09	Insulin

13729	Insulin isophane human emp 100unit/ml Injection	6010102	Feb-09	Insulin
13819	Hypurin Porcine Isophane 100units/ml suspension for injection 1.5ml cartridges (C P Pharmaceuticals Ltd)	0	Feb-09	Insulin
13837	Insulin biphasic isophane human prb 10:90; 100 units/ml Injection	6010102	Feb-09	Insulin
13969	U100 Insulin syringe 0.3ml	6010103	Feb-09	Insulin
14191	Autopen Junior hypodermic insulin injection pen reusable for 3ml cartridge 2 unit dial up / range 2-42 units (Owen Mumford Ltd)	0	Feb-09	Insulin
14270	Humalog Mix25 Pen 100units/ml suspension for injection 3ml pre-filled pen (Eli Lilly and Company Ltd)	6010102	Feb-09	Insulin
14290	Insulatard Penfill 100units/ml suspension for injection 3ml cartridges (Novo Nordisk Ltd)	6010102	Feb-09	Insulin
14299	Insulin glulisine 100units/ml solution for injection 3ml cartridges	6010101	Feb-09	Insulin
14301	Insulin detemir 100units/ml solution for injection 3ml cartridges	6010102	Feb-09	Insulin

14313	Insulin lispro 100units/ml solution for injection 3ml cartridges	6010101	Feb-09	Insulin
14330	Insulin detemir 100units/ml solution for injection 3ml pre-filled disposable devices	6010102	Feb-09	Insulin
14339	Hypurin Bovine Neutral 100units/ml solution for injection 10ml vials (Wockhardt UK Ltd)	6010101	Feb-09	Insulin
14340	Hypurin Bovine Isophane 100units/ml suspension for injection 10ml vials (Wockhardt UK Ltd)	6010102	Feb-09	Insulin
14345	Apidra 100units/ml solution for injection 3ml cartridges (Sanofi)	6010101	Feb-09	Insulin
14357	Humulin I 100units/ml suspension for injection 3ml cartridges (Eli Lilly and Company Ltd)	6010102	Feb-09	Insulin
14362	Insulin lispro 100units/ml solution for injection 3ml pre-filled disposable devices	6010101	Feb-09	Insulin
14504	INSULIN HYPURIN PROTAMINE ZINC 100 I/U INJ	0	Feb-09	Insulin
14505	Insulin protamine zinc bovine 100units/ml	6010102	Feb-09	Insulin

	suspension for injection 10ml vials			
14506	INSULIN BOVINE PROTAMINE ZINC 100 I/U INJ	0	Feb-09	Insulin
14619	Insulin isophane biphasic porcine 30/70 100units/ml suspension for injection 1.5ml cartridges	0	Feb-09	Insulin
14642	Bd m 0.3ml Insulin syringe with 29gauge needle 12mm (Becton, Dickinson UK Ltd)	6010103	Feb-09	Insulin
14644	Insulin biphasic isophane human prb 20:80; 100 units/ml Injection	6010102	Feb-09	Insulin
14646	Hypodermic U100 insulin syringe sterile single use / single patient use 0.3ml with 12mm needle 0.33mm/29gauge	71190300	Feb-09	Insulin
14649	Insulin biphasic isophane human pyr 10:90; 100 units/ml Injection	6010102	Feb-09	Insulin
14887	Hypodermic insulin needles for pre-filled / reusable pen injectors screw on 5mm/31gauge	71190600	Feb-09	Insulin
14918	Humulin I 100units/ml suspension for injection	6010102	Feb-09	Insulin

	10ml vials (Eli Lilly and Company Ltd)			
14925	Insulin isophane human vial 100unit/ml Sterile suspension injection	6010102	Feb-09	Insulin
14928	Insulatard 100units/ml suspension for injection 10ml vials (Novo Nordisk Ltd)	6010102	Feb-09	Insulin
14930	Hypurin Porcine Neutral 100units/ml solution for injection 3ml cartridges (Wockhardt UK Ltd)	6010101	Feb-09	Insulin
14933	Hypurin Porcine Isophane 100units/ml suspension for injection 3ml cartridges (Wockhardt UK Ltd)	6010102	Feb-09	Insulin
14938	Insulin soluble bovine cartridge 100unit/ml Solution for injection	06010101/06010300	Feb-09	Insulin
14944	Humulin S 100units/ml solution for injection 3ml cartridges (Eli Lilly and Company Ltd)	6010101	Feb-09	Insulin
15040	INSULIN MONOPHANE (ISOPHANE) 100 I/U INJ	0	Feb-09	Insulin
15199	Insuman comb 25 100iu/ml Injection (Aventis Pharma)	6010102	Feb-09	Insulin
15294	Innovo hypodermic insulin injection pen reusable for	0	Feb-09	Insulin

	3ml cartridge 1 unit dial up / range 1-70 units Green (Novo Nordisk Ltd)			
15484	Insulin isophane bovine 100units/ml suspension for injection 1.5ml cartridges	0	Feb-09	Insulin
15624	INSULIN ISOPHANE (HIGHLY PURIFIED) 100 I/U INJ	0	Feb-09	Insulin
15710	Insulin soluble human emp 100unit/ml Injection	06010101/06010300	Feb-09	Insulin
15895	Innovo hypodermic insulin injection pen reusable for 3ml cartridge 1 unit dial up / range 1-70 units Orange (Novo Nordisk Ltd)	0	Feb-09	Insulin
15951	Autopen Junior hypodermic insulin injection pen reusable for 3ml cartridge 1 unit dial up / range 1-21 units (Owen Mumford Ltd)	0	Feb-09	Insulin
15961	Insulin isophane human crb 100iu/ml Injection	6010102	Feb-09	Insulin
16129	Insulin soluble human 100units/ml solution for injection 3ml cartridges	6010101	Feb-09	Insulin
16142	Insulin aspart 100units/ml solution for injection 3ml cartridges	6010101	Feb-09	Insulin
16152	Insulin isophane biphasic human 30/70 100units/ml	6010151	Feb-09	Insulin

	suspension for injection 3ml cartridges			
16160	Humulin M3 Pen 100units/ml suspension for injection 3ml pre-filled pen (Eli Lilly and Company Ltd)	0	Feb-09	Insulin
16209	INSULIN HYPURIN SOLUBLE 100 I/U INJ	0	Feb-09	Insulin
16389	Myjector hypodermic U100 insulin syringe sterile single use / single patient use 1ml with 12mm needle 0.33mm/29gauge (Terumo UK Ltd)	71190300	Feb-09	Insulin
16682	Tempulin 100unit/ml Injection (Knoll Ltd)	6010102	Feb-09	Insulin
16700	Insulin zinc mixed bovine vial 100unit/ml Sterile suspension injection	6010102	Feb-09	Insulin
16866	Unifine hypodermic U100 insulin syringe sterile single use / single patient use 0.3ml with 12mm needle 0.33mm/29gauge (Owen Mumford Ltd)	71190300	Feb-09	Insulin
16959	Hypodermic U100 insulin syringe sterile single use / single patient use 1ml with 12mm needle 0.3mm/30gauge	71190300	Feb-09	Insulin

17076	Omnican 100 hypodermic U100 insulin syringe sterile single use / single patient use 1ml with 8mm needle 0.3mm/30gauge (B.Braun Medical Ltd)	71190300	Feb-09	Insulin
17336	Novopen 100unit/ml Injection device (Novo Nordisk Ltd)	06010101/06010300	Feb-09	Insulin
17377	Novopen inj device Injection device (Novo Nordisk Ltd)	6010103	Feb-09	Insulin
17405	Novopen classic inj device Injection device (Novo Nordisk Ltd)	6010103	Feb-09	Insulin
17643	Autopen Special Edition hypodermic insulin injection pen reusable for 3ml cartridge 2 unit dial up / range 2-42 units (Owen Mumford Ltd)	0	Feb-09	Insulin
17712	Hypurin Bovine Lente 100units/ml suspension for injection 10ml vials (Wockhardt UK Ltd)	6010102	Feb-09	Insulin
17731	Penmix 50/50 100iu/ml Injection (Novo Nordisk Ltd)	6010102	Feb-09	Insulin
17809	Humaject m4 100iu/ml M4 pen (Eli Lilly and Company Ltd)	6010102	Feb-09	Insulin

18149	Monoject u100 insulin syringe 12mm(29g)0.5ml 12mm29G 0.5ml Insulin syringe (Covidien (UK) Commercial Ltd)	6010103	Feb-09	Insulin
18195	Hypodermic insulin needles for pre-filled / reusable pen injectors snap on 6mm/31gauge	71190700	Feb-09	Insulin
18208	Hypodermic insulin needles for pre-filled / reusable pen injectors snap on 10mm/29gauge	71190700	Feb-09	Insulin
18224	Humalog 100units/ml solution for injection 10ml vials (Eli Lilly and Company Ltd)	6010101	Feb-09	Insulin
18301	INSULIN SOLUBLE INJ I/U^2	0	Feb-09	Insulin
18446	Unifine hypodermic U100 insulin syringe sterile single use / single patient use 1ml with 12mm needle 0.33mm/29gauge (Owen Mumford Ltd)	71190300	Feb-09	Insulin
18461	Insulin zinc mixed human 100units/ml suspension for injection 10ml vials	0	Feb-09	Insulin
18590	Insulin isophane bovine 100units/ml suspension for injection 10ml vials	6010102	Feb-09	Insulin

18592	Insulin soluble bovine 100units/ml solution for injection 10ml vials	6010101	Feb-09	Insulin
18593	Humalog Mix50 100units/ml suspension for injection 3ml cartridges (Eli Lilly and Company Ltd)	6010102	Feb-09	Insulin
18645	INSULIN NEUTRAL (PURIFIED) 100 I/U INJ	0	Feb-09	Insulin
18931	Insulin zinc crystalline human 100units/ml suspension for injection 10ml vials	0	Feb-09	Insulin
19029	SYRINGE PRE-SET INSULIN FOR BLIND 2ML	0	Feb-09	Insulin
19271	Hypodermic insulin needles for pre-filled / reusable pen injectors screw on 8mm/30gauge	71190600	Feb-09	Insulin
19491	Apidra 100units/ml solution for injection 10ml vials (Sanofi)	6010101	Feb-09	Insulin
19513	Humulin M3 100units/ml suspension for injection 10ml vials (Eli Lilly and Company Ltd)	6010151	Feb-09	Insulin
19707	INSULIN HUMULIN S (NEUTRAL SOLUBLE)	0	Feb-09	Insulin
19829	INSULIN NOVO MONOTARD MC	0	Feb-09	Insulin

19877	Insulin aspart 100units/ml solution for injection 3ml pre-filled disposable devices	6010101	Feb-09	Insulin
19878	Insulin isophane biphasic human 30/70 100units/ml suspension for injection 3ml pre-filled disposable devices	6010151	Feb-09	Insulin
19977	Omnican 50 hypodermic U100 insulin syringe sterile single use / single patient use 0.5ml with 12mm needle 0.3mm/30gauge (B.Braun Medical Ltd)	71190300	Feb-09	Insulin
20195	INSULIN BOVINE PROTAMINE ZINC 40 I/U INJ	0	Feb-09	Insulin
20196	INSULIN SOLUBLE 40 I/U INJ	0	Feb-09	Insulin
20422	Insuman comb 15 100iu/ml Injection (Aventis Pharma)	6010102	Feb-09	Insulin
20671	INSULIN HUM/ACTRAPHANE	0	Feb-09	Insulin
20672	INSULIN HUM/ACTRAPID	0	Feb-09	Insulin
20995	Hypurin Porcine 30/70 Mix 100units/ml suspension for injection 3ml cartridges (Wockhardt UK Ltd)	6010151	Feb-09	Insulin
21110	Insulin biphasic isophane human prb 50:50; 100 units/ml Injection	6010102	Feb-09	Insulin
21223	U100 Insulin syringe 0.3ml	6010103	Feb-09	Insulin

21232	Insulin isophane biphasic human 30/70 100units/ml suspension for injection 10ml vials	6010151	Feb-09	Insulin
21235	Humulin S 100units/ml solution for injection 10ml vials (Eli Lilly and Company Ltd)	6010101	Feb-09	Insulin
21347	Penmix 40/60 100iu/ml Injection (Novo Nordisk Ltd)	6010102	Feb-09	Insulin
21374	Insulin biphasic isophane human prb 40:60; 100 units/ml Injection	6010102	Feb-09	Insulin
21395	Insulin biphasic isophane human pyr 40:60; 100 units/ml Injection	6010102	Feb-09	Insulin
21422	Insulin isophane biphasic human 40/60 100units/ml suspension for injection 3ml cartridges	0	Feb-09	Insulin
21459	SYRINGE INSULIN U100 S/U+8MM NEEDLE	0	Feb-09	Insulin
21554	Insuman comb 50 100iu/ml Injection (Aventis Pharma)	6010102	Feb-09	Insulin
21583	Apidra 100units/ml solution for injection 3ml pre-filled OptiSet pen (Sanofi)	6010101	Feb-09	Insulin
21590	Insulin glulisine 100units/ml solution for injection 3ml pre-filled disposable devices	6010101	Feb-09	Insulin

21945	INSULIN PORK INSULATARD	0	Feb-09	Insulin
22058	Pur-in mix 15/85 Injection (C P Pharmaceuticals Ltd)	6010102	Feb-09	Insulin
22060	Insulin 0.5 0.5ml Syringe	59010200	Feb-09	Insulin
22094	INSULIN HUMULIN M2 VIAL	0	Feb-09	Insulin
22155	Humaject m5 100iu/ml M5 pen (Eli Lilly and Company Ltd)	6010102	Feb-09	Insulin
22161	INSULIN HUMULIN M1 VIAL	0	Feb-09	Insulin
22328	Unifine single use Insulin syringe with 30gauge needle 8mm 0.5ml (Owen Mumford Ltd)	6010103	Feb-09	Insulin
22496	INSULIN ZINC LENTE PURIFIED SUSPENSION	0	Feb-09	Insulin
22697	Insulin isophane biphasic human 50/50 100units/ml suspension for injection 1.5ml cartridges	0	Feb-09	Insulin
22806	INSULIN PORK ACTRAPID	0	Feb-09	Insulin
22823	INSULIN ISOPHANE (PURIFIED) 100 I/U INJ	0	Feb-09	Insulin
22945	Insuman rapid 100iu/ml Injection (Aventis Pharma)	06010101/06010300	Feb-09	Insulin
22946	Omnican 100 hypodermic U100 insulin syringe sterile single use / single patient use 1ml with 12mm needle	71190300	Feb-09	Insulin

	0.3mm/30gauge (B.Braun Medical Ltd)			
22974	Hypodermic insulin needles for pre-filled / reusable pen injectors snap on 8mm/31gauge	71190700	Feb-09	Insulin
22983	Insuman Rapid 100units/ml solution for injection 3ml cartridges (Sanofi)	6010101	Feb-09	Insulin
22987	Hypodermic insulin needles for pre-filled / reusable pen injectors snap on 12mm/29gauge	71190700	Feb-09	Insulin
23003	INSULIN ISOPHANE (NPH) 40 I/U	0	Feb-09	Insulin
23099	Insulin aspart biphasic 30/70 100units/ml suspension for injection 3ml pre-filled disposable devices	6010151	Feb-09	Insulin
23231	Hypurin Bovine Neutral 100units/ml solution for injection 3ml cartridges (Wockhardt UK Ltd)	6010101	Feb-09	Insulin
23437	Hypodermic insulin needles for pre-filled / reusable pen injectors screw on 12mm/28gauge	71190600	Feb-09	Insulin
23636	Insulin 1ml disposable syringe	59010200	Feb-09	Insulin

23992	Insuman Basal 100units/ml suspension for injection 3ml pre-filled OptiSet pen (Sanofi)	6010102	Feb-09	Insulin
23993	Insuman Rapid 100units/ml solution for injection 3ml pre-filled OptiSet pen (Sanofi)	6010101	Feb-09	Insulin
24002	Insuman Comb 25 100units/ml suspension for injection 5ml vials (Sanofi)	6010151	Feb-09	Insulin
24485	INSULIN ZINC ANIMAL SUSPENSION	0	Feb-09	Insulin
24554	Unifine single use Insulin syringe with 30gauge needle 8mm 0.3ml (Owen Mumford Ltd)	6010103	Feb-09	Insulin
24593	Neutral insulin bovine 100unit/ml Injection	06010101/06010300	Feb-09	Insulin
24722	INSULIN ISOPHANE 50%/NEUTRAL 50% 100 I/U INJ	0	Feb-09	Insulin
24795	Insulin aspart biphasic 30/70 100units/ml suspension for injection 3ml cartridges	6010151	Feb-09	Insulin
24800	Hypurin Porcine 30/70 Mix 100units/ml suspension for injection 10ml vials (Wockhardt UK Ltd)	6010151	Feb-09	Insulin

24845	INSULIN PUR-IN ISOPHANE 100 I/U INJ	0	Feb-09	Insulin
24846	Pur-in neutral 100unit/ml Injection (C P Pharmaceuticals Ltd)	06010101/06010300	Feb-09	Insulin
24866	INSULIN INSULATARD (LEO RETARD) 40 I/U INJ	0	Feb-09	Insulin
24993	Insuman Comb 25 100units/ml suspension for injection 3ml cartridges (Sanofi)	6010151	Feb-09	Insulin
25006	INSULIN HUMAN ACTRAPID (NEUTRAL)	0	Feb-09	Insulin
25133	Insuman Comb 25 100units/ml suspension for injection 3ml pre-filled OptiSet pen (Sanofi)	6010151	Feb-09	Insulin
25422	Hypoguard u100 click/count 1ml Insulin syringe (Hypoguard Ltd)	6010103	Feb-09	Insulin
25479	Insulin soluble porcine 100units/ml solution for injection 3ml cartridges	6010101	Feb-09	Insulin
25735	Insulin isophane biphasic human 20/80 100units/ml suspension for injection 3ml cartridges	0	Feb-09	Insulin
25736	Insulin isophane biphasic human 10/90 100units/ml	0	Feb-09	Insulin

	suspension for injection 3ml cartridges			
25786	Comfort Point hypodermic insulin needles for pre-filled / reusable pen injectors screw on 6mm/31gauge (Disposable Medical Equipment Ltd)	71190600	Feb-09	Insulin
25812	Insulin isophane human 100units/ml suspension for injection 3ml pre-filled disposable devices	6010102	Feb-09	Insulin
26060	Insulin lispro 100units/ml solution for injection 10ml vials	6010101	Feb-09	Insulin
26098	Hypurin Porcine Neutral 100units/ml solution for injection 10ml vials (Wockhardt UK Ltd)	6010101	Feb-09	Insulin
26338	Injex starter set 60500 (Ocon Chemicals Ltd)	71190100	Feb-09	Insulin
26403	Pur-in mix 25/75 Injection (C P Pharmaceuticals Ltd)	6010102	Feb-09	Insulin
26498	Insulin zinc suspension mixed bovine and porcine 100unit/ml Injection	6010102	Feb-09	Insulin
26621	Insulin soluble human crb 100iu/ml Injection	06010101/06010300	Feb-09	Insulin
26784	INSULIN ZINC SEMILENTE SUSP BP 100 I/U INJ	0	Feb-09	Insulin

26795	SYRINGE INSULIN DISPOSABLE	0	Feb-09	Insulin
27149	NOVOPEN INJ DEVICE	0	Feb-09	Insulin
27151	SYRINGE INSULIN U100 S/U+8MM NEEDLE	0	Feb-09	Insulin
27177	Insulin biphasic lispro human prb 50:50; 100 units/ml Injection	6010102	Feb-09	Insulin
27280	Insulin isophane biphasic porcine 30/70 100units/ml suspension for injection 10ml vials	6010151	Feb-09	Insulin
27396	Insulin soluble porcine 100units/ml solution for injection 10ml vials	6010101	Feb-09	Insulin
27402	Insulin soluble human 100units/ml solution for injection 10ml vials	6010101	Feb-09	Insulin
27461	Insuman Basal 100units/ml suspension for injection 3ml cartridges (Sanofi)	6010102	Feb-09	Insulin
27614	Penmix 30/70 100iu/ml Injection (Novo Nordisk Ltd)	6010102	Feb-09	Insulin
27911	INSULIN HUMAN ACTRAPID PENFILL	0	Feb-09	Insulin
28096	Insulin isophane biphasic human 50/50 100units/ml suspension for injection 3ml cartridges	6010151	Feb-09	Insulin

28101	Insulin glulisine 100units/ml solution for injection 10ml vials	6010101	Feb-09	Insulin
28183	Hypurin Porcine Isophane 100units/ml suspension for injection 10ml vials (Wockhardt UK Ltd)	6010102	Feb-09	Insulin
28185	Insulin lispro biphasic 25/75 100units/ml suspension for injection 3ml cartridges	6010102	Feb-09	Insulin
28442	Insulin glulisine 100unit/ml Solution for injection	6010101	Feb-09	Insulin
28588	Hypurin Bovine Isophane 100units/ml suspension for injection 3ml cartridges (Wockhardt UK Ltd)	6010102	Feb-09	Insulin
28666	Omnican 50 hypodermic U100 insulin syringe sterile single use / single patient use 0.5ml with 8mm needle 0.3mm/30gauge (B.Braun Medical Ltd)	71190300	Feb-09	Insulin
28723	INSULIN ZINC BOVINE SUSPENSION	0	Feb-09	Insulin
28978	INSULIN PUR-IN MIX 15/85 100 I/U INJ	0	Feb-09	Insulin
29090	Novopen fun inj device Injection device (Novo Nordisk Ltd)	6010103	Feb-09	Insulin

29567	Insulin aspart 100units/ml solution for injection 10ml vials	6010101	Feb-09	Insulin
29837	Insulin biphasic isophane human prb 25:75; 100 units/ml Injection	6010102	Feb-09	Insulin
29953	Apidra 100units/ml solution for injection 3ml OptiClik cartridges (Sanofi)	6010101	Feb-09	Insulin
30209	Actrapid mc 100unit/ml Injection (Arun Products Ltd)	06010101/06010300	Feb-09	Insulin
30236	Isophane insulin 100iu/ml Injection	6010102	Feb-09	Insulin
30305	Hypodermic U100 insulin syringe sterile single use / single patient use 0.5ml with 12mm needle 0.3mm/30gauge	71190300	Feb-09	Insulin
30686	Insulin isophane porcine 100units/ml suspension for injection 3ml cartridges	6010102	Feb-09	Insulin
30819	Insuman Comb 15 100units/ml suspension for injection 3ml pre-filled OptiSet pen (Sanofi)	6010151	Feb-09	Insulin
30861	INSULIN ZINC HUMAN SUSPENSION	0	Feb-09	Insulin
30918	ABCare hypodermic U100 insulin syringe glass	0	Feb-09	Insulin

	reusable 0.5ml (Rand Rocket Ltd)			
31205	Insuman Comb 50 100units/ml suspension for injection 3ml pre-filled OptiSet pen (Sanofi)	6010151	Feb-09	Insulin
31258	Insulin lispro biphasic 25/75 100units/ml suspension for injection 3ml pre-filled disposable devices	6010102	Feb-09	Insulin
31267	INSULIN PUR-IN MIX 50/50 100 I/U INJ	0	Feb-09	Insulin
31464	Exubera kit (Pfizer Ltd)	0	Feb-09	Insulin
31465	Exubera 1mg inhalation powder blisters (Pfizer Ltd)	0	Feb-09	Insulin
31466	Exubera insulin release unit (Pfizer Ltd)	0	Feb-09	Insulin
31467	Exubera 3mg inhalation powder blisters (Pfizer Ltd)	0	Feb-09	Insulin
31699	ABCare hypodermic U100 insulin syringe glass reusable 1ml (Rand Rocket Ltd)	0	Feb-09	Insulin
32053	INSULIN HUMALOG MIX 25	0	Feb-09	Insulin
33101	Hypodermic insulin needles for pre-filled / reusable pen injectors screw on 12.7mm/29gauge	71190600	Feb-09	Insulin

33167	Insulin biphasic isophane human crb 25:75; 100 units/ml Injection	6010102	Feb-09	Insulin
33232	Insulin isophane biphasic human 50/50 100units/ml suspension for injection 5ml vials	0	Feb-09	Insulin
33356	Hypodermic insulin needles for pre-filled / reusable pen injectors screw on 12mm/29gauge	71190600	Feb-09	Insulin
33914	Omnican 30 12mm 29G 0.3ml Syringe (B.Braun Medical Ltd)	6010103	Feb-09	Insulin
33966	Insulatard 100unit/ml Injection (Novo Nordisk Ltd)	6010102	Feb-09	Insulin
34031	Monotard mc 100unit/ml Injection (Novo Nordisk Ltd)	6010102	Feb-09	Insulin
34097	Human initard 50/50 100unit/ml Injection (Novo Nordisk Ltd)	6010102	Feb-09	Insulin
34713	Insulin 1 ml syringe	59010200	Feb-09	Insulin
35017	Optipen pro 1 white insulin pen 3ml/1-60 units 3ml/1-60 units Insulin pen (Aventis Pharma)	6010103	Feb-09	Insulin
35057	OptiClik hypodermic insulin injection pen reusable for 3ml cartridge 1 unit dial up /	71190900	Feb-09	Insulin

	range 1-80 units Blue (Sanofi)			
35078	Optipen pro 1 blue insulin pen 3ml/1-60 units 3ml/1-60 units Insulin pen (Aventis Pharma)	6010103	Feb-09	Insulin
35081	OptiClik hypodermic insulin injection pen reusable for 3ml cartridge 1 unit dial up / range 1-80 units Grey (Sanofi)	71190900	Feb-09	Insulin
35143	NovoFine Autocover hypodermic insulin needles for pre-filled / reusable pen injectors screw on 8mm/30gauge (Novo Nordisk Ltd)	71190600	Feb-09	Insulin
35218	Optipen pro 1 yellow insulin pen 3ml/1-60 units 3ml/1-60 units Insulin pen (Aventis Pharma)	6010103	Feb-09	Insulin
35253	Insuman Comb 50 100units/ml suspension for injection 3ml cartridges (Sanofi)	6010151	Feb-09	Insulin
35260	Levemir InnoLet 100units/ml solution for injection 3ml pre-filled pen (Novo Nordisk Ltd)	6010102	Feb-09	Insulin

35454	Comfort Point hypodermic insulin needles for pre-filled / reusable pen injectors screw on 8mm/31gauge (Disposable Medical Equipment Ltd)	71190600	Feb-09	Insulin
35468	Insuman Basal 100units/ml suspension for injection 5ml vials (Sanofi)	6010102	Feb-09	Insulin
35701	Insulin lispro biphasic 50/50 100units/ml suspension for injection 3ml pre-filled disposable devices	6010102	Feb-09	Insulin
36031	Insulin isophane biphasic porcine 30/70 100units/ml suspension for injection 3ml cartridges	6010151	Feb-09	Insulin
36043	Exubera chamber (Pfizer Ltd)	0	Feb-09	Insulin
36066	Insulin isophane bovine 100units/ml suspension for injection 3ml cartridges	6010102	Feb-09	Insulin
36146	Insulin lispro biphasic 50/50 100units/ml suspension for injection 3ml cartridges	6010102	Feb-09	Insulin
36194	Insulin isophane biphasic human 25/75 100units/ml suspension for injection 3ml cartridges	6010151	Feb-09	Insulin

36355	Insulin human 1mg inhalation powder blisters	0	Feb-09	Insulin
36356	Insulin human 3mg inhalation powder blisters	0	Feb-09	Insulin
36430	Insulin soluble human 100units/ml solution for injection 3ml pre-filled disposable devices	6010101	Feb-09	Insulin
36513	Velosulin cartridge 100unit/ml Injection (Novo Nordisk Ltd)	06010101/06010300	Feb-09	Insulin
36853	Lantus 100units/ml solution for injection 3ml pre-filled SoloStar pen (Sanofi)	6010102	Feb-09	Insulin
36920	Apidra 100units/ml solution for injection 3ml pre-filled SoloStar pen (Sanofi)	6010101	Feb-09	Insulin
36959	Comfort Point hypodermic insulin needles for pre-filled / reusable pen injectors screw on 12mm/29gauge (Exel Int. (Europe) Ltd)	71190600	Feb-09	Insulin
37055	Comfort Point hypodermic U100 insulin syringe sterile single use / single patient use 0.5ml with 12mm needle 0.33mm/29gauge (Exel Int. (Europe) Ltd)	71190300	Feb-09	Insulin
37427	HumaPen Luxura HD hypodermic insulin injection	71190900	Feb-09	Insulin

	pen reusable for 3ml cartridge 0.5 unit dial up / range 1-30 units (Eli Lilly and Company Ltd)			
38093	Insulin 1ml pre-set syringe	59010200	Feb-09	Insulin
38236	Comfort Point hypodermic U100 insulin syringe sterile single use / single patient use 1ml with 12mm needle 0.33mm/29gauge (Disposable Medical Equipment Ltd)	71190300	Feb-09	Insulin
38422	Isophane 100iu/ml Injection (Celltech Pharma Europe Ltd)	6010102	Feb-09	Insulin
38774	NovoPen 4 hypodermic insulin injection pen reusable for 3ml cartridge 1 unit dial up / range 1-60 units Blue (Novo Nordisk Ltd)	71190900	Feb-09	Insulin
38808	NovoPen 4 hypodermic insulin injection pen reusable for 3ml cartridge 1 unit dial up / range 1-60 units Silver (Novo Nordisk Ltd)	71190900	Feb-09	Insulin
38986	Humalog KwikPen 100units/ml solution for	6010101	Feb-09	Insulin

	injection 3ml pre-filled pen (Eli Lilly and Company Ltd)			
39006	Humalog Mix25 KwikPen 100units/ml suspension for injection 3ml pre-filled pen (Eli Lilly and Company Ltd)	6010102	Feb-09	Insulin
39086	Humalog Mix50 KwikPen 100units/ml suspension for injection 3ml pre-filled pen (Eli Lilly and Company Ltd)	6010102	Apr-09	Insulin
39150	Hypodermic U100 insulin syringe sterile single use / single patient use 1ml with 12mm safety needle 0.33mm/29gauge	71190300	Apr-09	Insulin
40085	Insulin 2ml syringe	59010200	Aug-09	Insulin
40555	HumaPen Memoir hypodermic insulin injection pen reusable for 3ml cartridge 1 unit dial up / range 1-60 units (Eli Lilly and Company Ltd)	71190900	Oct-09	Insulin
41120	Insulin isophane biphasic human 50/50 100units/ml suspension for injection 3ml pre-filled disposable devices	6010151	Jan-10	Insulin
41834	Insulin zinc suspension lente 100iu/ml Injection (Celltech Pharma Europe Ltd)	6010102	Apr-10	Insulin

41959	Penject 100unit/ml Injection device (Hypoguard Ltd)	06010101/06010300	Apr-10	Insulin
42305	Injex 10ml vial adaptor pack 60502 (Ocon Chemicals Ltd)	71190100	May-10	Insulin
42395	Humalog Mix25 100units/ml suspension for injection 10ml vials (Eli Lilly and Company Ltd)	6010102	May-10	Insulin
42797	Injex ampoule pack 60501 (Ocon Chemicals Ltd)	71190100	Jul-10	Insulin
42954	Insulin isophane biphasic human 25/75 100units/ml suspension for injection 5ml vials	6010151	Jul-10	Insulin
43489	BD Micro-Fine Ultra hypodermic insulin needles for pre-filled / reusable pen injectors screw on 4mm/32gauge (Becton, Dickinson UK Ltd)	71190600	Sep-10	Insulin
43568	ClikSTAR hypodermic insulin injection pen reusable for 3ml cartridge 1 unit dial up / range 1-80 units Silver (Sanofi)	71190900	Sep-10	Insulin
43670	Hypodermic insulin needles for pre-filled / reusable pen injectors screw on 4mm/32gauge	71190600	Oct-10	Insulin

43833	ClikSTAR hypodermic insulin injection pen reusable for 3ml cartridge 1 unit dial up / range 1-80 units Blue (Sanofi)	71190900	Nov-10	Insulin
43950	Humulin I KwikPen 100units/ml suspension for injection 3ml pre-filled pen (Eli Lilly and Company Ltd)	6010102	Nov-10	Insulin
43953	Insulin lispro biphasic 25/75 100units/ml suspension for injection 10ml vials	6010102	Nov-10	Insulin
43991	Humulin M3 KwikPen 100units/ml suspension for injection 3ml pre-filled pen (Eli Lilly and Company Ltd)	6010151	Dec-10	Insulin
44251	Insulin zinc suspension mixed porcine 100unit/ml Injection	6010102	Jan-11	Insulin
44378	Insulin isophane biphasic human 25/75 100units/ml suspension for injection 3ml pre-filled disposable devices	6010151	Jan-11	Insulin
44480	Insuman Comb 25 100units/ml suspension for injection 3ml pre-filled SoloStar pen (Sanofi)	6010151	Feb-11	Insulin
44601	Injex 4 monthly refill pack 60504 (Ocon Chemicals Ltd)	71190100	Feb-11	Insulin

44810	Hypodermic insulin needles for pre-filled / reusable pen injectors screw on 5mm/32gauge	71190600	Mar-11	Insulin
45045	NovoTwist hypodermic insulin needles for pre-filled / reusable pen injectors screw on 5mm/32gauge (Novo Nordisk Ltd)	71190600	Apr-11	Insulin
45158	Insuman Comb 15 100units/ml suspension for injection 3ml cartridges (Sanofi)	6010151	May-11	Insulin
45639	IME-FINE hypodermic insulin needles for pre-filled / reusable pen injectors screw on 6mm/31gauge (Arctic Medical Ltd)	71190600	Jul-11	Insulin
46001	Insuman Basal 100units/ml suspension for injection 3ml pre-filled SoloStar pen (Sanofi)	6010102	Aug-11	Insulin
46666	NovoRapid FlexTouch 100units/ml solution for injection 3ml pre-filled pen (Novo Nordisk Ltd)	6010101	Jan-12	Insulin
47360	Neutral insulin 100unit/ml Injection (Celltech Pharma Europe Ltd)	06010101/06010300	Jun-12	Insulin
47588	Insulin 1ml click count	59010200	Aug-12	Insulin

47751	TS-A Safety hypodermic U100 insulin syringe sterile single use / single patient use 1ml with 12mm safety needle 0.33mm/29gauge (Minsmed)	71190300	Sep-12	Insulin
47856	Neuphane 100unit/ml Injection (Wellcome Medical Division)	6010102	Oct-12	Insulin
48342	HumaPen Luxura hypodermic insulin injection pen reusable for 3ml cartridge 1 unit dial up / range 1-60 units Burgundy (Eli Lilly and Company Ltd)	71190900	Jan-13	Insulin
48435	BD AutoShield hypodermic insulin needles for pre-filled / reusable pen injectors screw on 5mm/29gauge (Becton, Dickinson UK Ltd)	71190600	Jan-13	Insulin
48501	HumaPen Luxura hypodermic insulin injection pen reusable for 3ml cartridge 1 unit dial up / range 1-60 units Champagne (Eli Lilly and Company Ltd)	71190900	Jan-13	Insulin
48576	BD AutoShield Duo hypodermic insulin needles for pre-filled / reusable pen	71190600	Jan-13	Insulin

	injectors screw on 5mm/30gauge (Becton, Dickinson UK Ltd)			
48633	Comfort Point hypodermic insulin needles for pre-filled / reusable pen injectors screw on 4mm/31gauge (Disposable Medical Equipment Ltd)	71190600	Jan-13	Insulin
48765	Unifine Pentips hypodermic insulin needles for pre-filled / reusable pen injectors screw on 5mm/31gauge (Owen Mumford Ltd)	71190900	Jan-13	Insulin
48771	Unifine Pentips Plus hypodermic insulin needles for pre-filled / reusable pen injectors screw on 5mm/31gauge (Owen Mumford Ltd)	71190000	Jan-13	Insulin
48811	Unifine Pentips Plus hypodermic insulin needles for pre-filled / reusable pen injectors screw on 6mm/31gauge (Owen Mumford Ltd)	71190000	Jan-13	Insulin
48829	NovoPen Echo hypodermic insulin injection pen reusable for 3ml cartridge 0.5 unit dial up / range 0.5-	71190900	Jan-13	Insulin

	30 units Blue (Novo Nordisk Ltd)			
49052	Unifine Pentips Plus hypodermic insulin needles for pre-filled / reusable pen injectors screw on 8mm/31gauge (Owen Mumford Ltd)	71190000	Jan-13	Insulin
49108	NovoRapid Penfill 100units/ml solution for injection 3ml cartridges (Necessity Supplies Ltd)	6010101	Jan-13	Insulin
49172	NovoPen Echo hypodermic insulin injection pen reusable for 3ml cartridge 0.5 unit dial up / range 0.5-30 units Red (Novo Nordisk Ltd)	71190900	Jan-13	Insulin
49307	Mylife Clickfine hypodermic insulin needles for pre-filled / reusable pen injectors snap on 4.5mm/31gauge (Ypsomed Ltd)	71190000/71190600	Jan-13	Insulin
49451	Mylife Clickfine AutoProtect hypodermic insulin needles for pre-filled / reusable pen injectors screw on 8mm/29gauge (Ypsomed Ltd)	71190600	Jan-13	Insulin

49479	Hypodermic insulin needles for pre-filled / reusable pen injectors screw on 4mm/31gauge	71190600	Jan-13	Insulin
49506	Insujet needle free starter kit Starter kit (European Pharma Group BV)	6010103	Jan-13	Insulin
49507	InsuJet nozzle pack 012070GB15 (Spirit Healthcare Ltd)	71190100	Jan-13	Insulin
49508	InsuJet 10ml vial adaptor pack 01207GB1510 (Spirit Healthcare Ltd)	71190100	Jan-13	Insulin
49509	InsuJet starter set 012005GBSP Blue (Spirit Healthcare Ltd)	71190100	Jan-13	Insulin
49831	Lantus 100units/ml solution for injection 3ml pre-filled SoloStar pen (Necessity Supplies Ltd)	6010102	Jan-13	Insulin
50633	Lantus 100units/ml solution for injection 3ml cartridges (Necessity Supplies Ltd)	6010102	Jan-13	Insulin
50691	Human Mixtard 20 Penfill 100units/ml suspension for injection 1.5ml cartridges (Novo Nordisk Ltd)	0	Jan-13	Insulin
50798	Hypodermic insulin injection pen reusable for	71190900	Jan-13	Insulin

	3ml cartridge 0.5 unit dial up / range 1-35 units			
51107	Hypodermic insulin injection pen reusable for 3ml cartridge 2 unit dial up / range 2-42 units	71190900	Jan-13	Insulin
51182	InsuJet starter set 012003GBSP Lime (Spirit Healthcare Ltd)	71190100	Jan-13	Insulin
51612	Hypodermic insulin needles for pre-filled / reusable pen injectors screw on 5mm/29gauge	71190600	Jan-13	Insulin
51650	Omnican Fine hypodermic insulin needles for pre-filled / reusable pen injectors screw on 6mm/31gauge (B.Braun Medical Ltd)	71190600	Jan-13	Insulin
51743	NovoRapid Penfill 100units/ml solution for injection 3ml cartridges (Sigma Pharmaceuticals Plc)	6010101	Jan-13	Insulin
51836	InsuJet 3ml cartridge adaptor pack 012071GB1503 (Spirit Healthcare Ltd)	71190100	Jan-13	Insulin
51881	Omnican Fine hypodermic insulin needles for pre-filled / reusable pen injectors	71190600	Jan-13	Insulin

	screw on 8mm/31gauge (B.Braun Medical Ltd)			
52232	Hypodermic insulin injection pen reusable for 3ml cartridge 0.5 unit dial up / range 1-30 units	71190900	Jan-13	Insulin
52319	Hypodermic insulin injection pen reusable for 3ml cartridge 1 unit dial up / range 1-80 units	71190900	Jan-13	Insulin
52522	Humalog Mix50 KwikPen 100units/ml suspension for injection 3ml pre-filled pen (Doncaster Pharmaceuticals Ltd)	6010102	Jan-13	Insulin
52722	Human Mixtard 30 Penfill 100units/ml suspension for injection 1.5ml cartridges (Novo Nordisk Ltd)	0	Jan-13	Insulin
52748	Insulatard Penfill 100units/ml suspension for injection 3ml cartridges (Waymade Healthcare Plc)	6010102	Jan-13	Insulin
53118	NovoRapid FlexPen 100units/ml solution for injection 3ml pre-filled pen (Mawdsley-Brooks & Company Ltd)	6010101	Jan-13	Insulin
53148	Hypodermic insulin injection pen reusable for	71190900	Jan-13	Insulin

	3ml cartridge 1 unit dial up / range 1-60 units			
53251	NovoRapid Penfill 100units/ml solution for injection 3ml cartridges (DE Pharmaceuticals)	6010101	Jan-13	Insulin
53437	Hypodermic insulin needles for pre-filled / reusable pen injectors screw on 5mm/30gauge	71190600	Jan-13	Insulin
53710	Insulin human 500units/ml solution for injection 20ml vials	6010100	Feb-13	Insulin
54027	Hypodermic insulin needles for pre-filled / reusable pen injectors snap on 4.5mm/31gauge	71190000/71190600	Mar-13	Insulin
54028	Unifine Pentips Plus hypodermic insulin needles for pre-filled / reusable pen injectors screw on 12mm/29gauge (Owen Mumford Ltd)	71190000	Mar-13	Insulin
54462	Insulin biphasic isophane human emp 25:75; 100 units/ml Injection	6010102	Apr-13	Insulin
54573	Hypodermic insulin injection pen reusable for 3ml cartridge 1 unit dial up / range 1-21 units	71190900	Apr-13	Insulin

54629	Insupen hypodermic insulin needles for pre-filled / reusable pen injectors screw on 8mm/31gauge (Spirit Healthcare Ltd)	71190600	Apr-13	Insulin
54885	Hypodermic insulin needles for pre-filled / reusable pen injectors screw on 8mm/32gauge	71190000	May-13	Insulin
54886	Hypodermic insulin needles for pre-filled / reusable pen injectors screw on 6mm/32gauge	71190000	May-13	Insulin
55234	Tresiba FlexTouch 200units/ml solution for injection 3ml pre-filled pen (Novo Nordisk Ltd)	6010102	May-13	Insulin
55462	Tresiba FlexTouch 100units/ml solution for injection 3ml pre-filled pen (Novo Nordisk Ltd)	6010102	Jun-13	Insulin
55517	Insulin isophane human 100units/ml suspension for injection 10ml vials	6010102	Jun-13	Insulin
55521	Insupen hypodermic insulin needles for pre-filled / reusable pen injectors screw on 4mm/32gauge (Spirit Healthcare Ltd)	71190600	Jun-13	Insulin

55603	Humalog KwikPen 100units/ml solution for injection 3ml pre-filled pen (Doncaster Pharmaceuticals Ltd)	6010101	Jun-13	Insulin
55618	Levemir FlexPen 100units/ml solution for injection 3ml pre-filled pen (Waymade Healthcare Plc)	6010102	Jun-13	Insulin
55627	Insupen hypodermic insulin needles for pre-filled / reusable pen injectors screw on 5mm/31gauge (Spirit Healthcare Ltd)	71190600	Jun-13	Insulin
55687	Insulin degludec 100units/ml solution for injection 3ml pre-filled disposable devices	6010102	Jun-13	Insulin
55746	Insupen hypodermic insulin needles for pre-filled / reusable pen injectors screw on 8mm/32gauge (Spirit Healthcare Ltd)	71190000	Jun-13	Insulin
55907	Insulin degludec 100units/ml solution for injection 3ml cartridges	6010102	Aug-13	Insulin
55910	Tresiba Penfill 100units/ml solution for injection 3ml cartridges (Novo Nordisk Ltd)	6010102	Aug-13	Insulin

56115	Human Actrapid Penfill 100units/ml solution for injection 1.5ml cartridges (Novo Nordisk Ltd)	0	Aug-13	Insulin
56352	HumaPen Savvio hypodermic insulin injection pen reusable for 3ml cartridge 1 unit dial up / range 1-60 units Green (Eli Lilly and Company Ltd)	71190000	Aug-13	Insulin
56489	NovoMix 30 Penfill 100units/ml suspension for injection 3ml cartridges (Waymade Healthcare Plc)	6010151	Aug-13	Insulin
56495	Lantus 100units/ml solution for injection 3ml pre-filled OptiSet pen (Waymade Healthcare Plc)	0	Aug-13	Insulin
56502	Actrapid Penfill 100units/ml solution for injection 3ml cartridges (Novo Nordisk Ltd)	0	Aug-13	Insulin
56624	Kendall Magellan hypodermic U100 insulin syringe sterile single use / single patient use 0.5ml with 12mm safety needle 0.3mm/30gauge (Covidien (UK) Commercial Ltd)	71190300	Aug-13	Insulin

56639	HumaPen Savvio hypodermic insulin injection pen reusable for 3ml cartridge 1 unit dial up / range 1-60 units Blue (Eli Lilly and Company Ltd)	71190000	Aug-13	Insulin
56642	Microdot Droplet hypodermic insulin needles for pre-filled / reusable pen injectors screw on 6mm/31gauge (Cambridge Sensors Ltd)	71190600	Aug-13	Insulin
56656	Kendall Magellan hypodermic U100 insulin syringe sterile single use / single patient use 0.3ml with 8mm safety needle 0.3mm/30gauge (Covidien (UK) Commercial Ltd)	71190300	Aug-13	Insulin
56691	Insulin degludec 200units/ml solution for injection 3ml pre-filled disposable devices	6010102	Aug-13	Insulin
56785	HumaPen Savvio hypodermic insulin injection pen reusable for 3ml cartridge 1 unit dial up / range 1-60 units Red (Eli Lilly and Company Ltd)	71190000	Aug-13	Insulin

56808	Omnican Fine hypodermic insulin needles for pre-filled / reusable pen injectors screw on 12mm/29gauge (B.Braun Medical Ltd)	71190600	Aug-13	Insulin
56857	Insulin isophane biphasic human 15/85 100units/ml suspension for injection 3ml cartridges	6010151	Aug-13	Insulin
56879	HumaPen Savvio hypodermic insulin injection pen reusable for 3ml cartridge 1 unit dial up / range 1-60 units Graphite (Eli Lilly and Company Ltd)	71190000	Aug-13	Insulin
56939	HumaPen Savvio hypodermic insulin injection pen reusable for 3ml cartridge 1 unit dial up / range 1-60 units Pink (Eli Lilly and Company Ltd)	71190000	Aug-13	Insulin
56983	Insupen hypodermic insulin needles for pre-filled / reusable pen injectors screw on 6mm/31gauge (Spirit Healthcare Ltd)	71190600	Sep-13	Insulin
57153	Kendall Magellan hypodermic U100 insulin syringe sterile single use / single patient use 0.5ml	71190300	Sep-13	Insulin

	with 8mm safety needle 0.3mm/30gauge (Covidien (UK) Commercial Ltd)			
57243	Omnican Fine hypodermic insulin needles for pre-filled / reusable pen injectors screw on 10mm/30gauge (B.Braun Medical Ltd)	71190600	Sep-13	Insulin
57387	Kendall Magellan hypodermic U100 insulin syringe sterile single use / single patient use 0.3ml with 12mm safety needle 0.33mm/29gauge (Covidien (UK) Commercial Ltd)	71190300	Oct-13	Insulin
57388	Kendall Magellan hypodermic U100 insulin syringe sterile single use / single patient use 1ml with 8mm safety needle 0.3mm/30gauge (Covidien (UK) Commercial Ltd)	71190300	Oct-13	Insulin
57451	Microdot Droplet hypodermic insulin needles for pre-filled / reusable pen injectors screw on 8mm/31gauge (Cambridge Sensors Ltd)	71190600	Oct-13	Insulin
57493	HumaPen Savvio hypodermic insulin injection	71190000	Oct-13	Insulin

	pen reusable for 3ml cartridge 1 unit dial up / range 1-60 units Silver (Eli Lilly and Company Ltd)			
57529	Humalog 100units/ml solution for injection 10ml vials (Dowelhurst Ltd)	6010101	Oct-13	Insulin
57564	Humalog KwikPen 100units/ml solution for injection 3ml pre-filled pen (Waymade Healthcare Plc)	6010101	Oct-13	Insulin
57620	Humulin M3 100units/ml suspension for injection 10ml vials (Mawdsley-Brooks & Company Ltd)	0	Oct-13	Insulin
57622	Humalog Mix50 KwikPen 100units/ml suspension for injection 3ml pre-filled pen (Waymade Healthcare Plc)	6010102	Oct-13	Insulin
57744	Omnican Fine hypodermic insulin needles for pre-filled / reusable pen injectors screw on 4mm/31gauge (B.Braun Medical Ltd)	71190000	Oct-13	Insulin
58449	Unifine Pentips hypodermic insulin needles for pre-filled / reusable pen injectors screw on 4mm/32gauge (Owen Mumford Ltd)	71190900	Dec-13	Insulin

58578	Kendall Magellan hypodermic U100 insulin syringe sterile single use / single patient use 0.5ml with 12mm safety needle 0.33mm/29gauge (Covidien (UK) Commercial Ltd)	71190300	Jan-14	Insulin
58579	Hypodermic insulin needles for pre-filled / reusable pen injectors screw on 4mm/32.5gauge	71190000	Jan-14	Insulin
58581	GlucoRx FinePoint hypodermic insulin needles for pre-filled / reusable pen injectors screw on 5mm/31gauge (GlucoRx Ltd)	71190600	Jan-14	Insulin
58745	GlucoRx FinePoint hypodermic insulin needles for pre-filled / reusable pen injectors screw on 4mm/31gauge (GlucoRx Ltd)	71190600	Feb-14	Insulin
58754	BD SafetyGlide hypodermic U100 insulin syringe sterile single use / single patient use 1ml with 12mm safety needle 0.33mm/29gauge (Becton, Dickinson UK Ltd)	71190300	Feb-14	Insulin

58798	GlucoRx FinePoint hypodermic insulin needles for pre-filled / reusable pen injectors screw on 6mm/31gauge (GlucoRx Ltd)	71190600	Feb-14	Insulin
58801	GlucoRx FinePoint hypodermic insulin needles for pre-filled / reusable pen injectors screw on 10mm/29gauge (GlucoRx Ltd)	71190600	Feb-14	Insulin
58817	GlucoRx FinePoint hypodermic insulin needles for pre-filled / reusable pen injectors screw on 12mm/29gauge (GlucoRx Ltd)	71190600	Feb-14	Insulin
58878	Hypodermic insulin needles for pre-filled / reusable pen injectors screw on 10mm/29gauge	71190600	Feb-14	Insulin
58884	GlucoRx FinePoint hypodermic insulin needles for pre-filled / reusable pen injectors screw on 8mm/31gauge (GlucoRx Ltd)	71190600	Feb-14	Insulin
58961	Mylife Penfine Classic hypodermic insulin needles	06010103/71190600	Mar-14	Insulin

	for pre-filled / reusable pen injectors screw on 4mm/32gauge (Ypsomed Ltd)			
58995	BD SafetyGlide hypodermic U100 insulin syringe sterile single use / single patient use 0.5ml with 12mm safety needle 0.33mm/29gauge (Becton, Dickinson UK Ltd)	71190300	Mar-14	Insulin
59004	Mylife Penfine Classic hypodermic insulin needles for pre-filled / reusable pen injectors screw on 6mm/32gauge (Ypsomed Ltd)	06010103/71190600	Mar-14	Insulin
59005	Mylife Penfine Classic hypodermic insulin needles for pre-filled / reusable pen injectors screw on 8mm/31gauge (Ypsomed Ltd)	06010103/71190600	Mar-14	Insulin
59133	BD SafetyGlide hypodermic U100 insulin syringe sterile single use / single patient use 0.5ml with 8mm safety needle 0.3mm/30gauge (Becton, Dickinson UK Ltd)	71190300	Mar-14	Insulin
59243	BD SafetyGlide hypodermic U100 insulin syringe sterile	71190300	Mar-14	Insulin

	single use / single patient use 0.3ml with 8mm safety needle 0.25mm/31gauge (Becton, Dickinson UK Ltd)			
59311	Kendall Magellan hypodermic U100 insulin syringe sterile single use / single patient use 1ml with 12mm safety needle 0.33mm/29gauge (Covidien (UK) Commercial Ltd)	71190300	Apr-14	Insulin
59475	Nanopass hypodermic insulin needles for pre-filled / reusable pen injectors screw on 4mm/32.5gauge (Terumo UK Ltd)	71190000	Apr-14	Insulin
59500	Insulin isophane human 100units/ml suspension for injection 5ml vials	6010102	Apr-14	Insulin
59533	NovoRapid FlexPen 100units/ml solution for injection 3ml pre-filled pen (Sigma Pharmaceuticals Plc)	6010101	Apr-14	Insulin
59793	Microdot Droplet hypodermic insulin needles for pre-filled / reusable pen injectors screw on 4mm/32gauge (Cambridge Sensors Ltd)	71190600	May-14	Insulin

59846	Mylife Clickfine hypodermic insulin needles for pre-filled / reusable pen injectors snap on 4mm/32gauge (Ypsomed Ltd)	71190700	May-14	Insulin
60028	Hypodermic insulin needles for pre-filled / reusable pen injectors snap on 4mm/32gauge	71190700	May-14	Insulin
60609	Insupen hypodermic insulin needles for pre-filled / reusable pen injectors screw on 6mm/32gauge (Spirit Healthcare Ltd)	71190000	Jul-14	Insulin
60621	Hypodermic insulin injection pen reusable for 3ml cartridge 0.5 unit dial up / range 0.5-30 units	71190900	Jul-14	Insulin
60626	InsuJet starter set 012007GBSP Grey (Spirit Healthcare Ltd)	71190100	Jul-14	Insulin
60750	Hypodermic insulin needles for pre-filled / reusable pen injectors screw on 4mm/33gauge	71190600	Aug-14	Insulin
60933	Humulin M3 100units/ml suspension for injection 10ml vials (Sigma Pharmaceuticals Plc)	6010151	Aug-14	Insulin

60938	Mixtard 30 100units/ml suspension for injection 10ml vials (Waymade Healthcare Plc)	0	Aug-14	Insulin
60951	Insulin human 100units/ml solution for injection 10ml vials	6010100	Aug-14	Insulin
60967	Nanopass hypodermic insulin needles for pre-filled / reusable pen injectors screw on 8mm/32.5gauge (Terumo UK Ltd)	71190000	Aug-14	Insulin
61562	Hypodermic insulin needles for pre-filled / reusable pen injectors screw on 8mm/29gauge	71190600	Nov-14	Insulin
5678	Nateglinide 120mg tablets	6010203	Feb-09	Meglitinide
5989	Nateglinide 180mg tablets	6010203	Feb-09	Meglitinide
9707	Repaglinide 1mg tablets	6010203	Feb-09	Meglitinide
9748	Repaglinide 2mg tablets	6010203	Feb-09	Meglitinide
9865	Repaglinide 500microgram tablets	6010203	Feb-09	Meglitinide
11316	NovoNorm 500microgram tablets (Novo Nordisk Ltd)	6010203	Feb-09	Meglitinide
11321	NovoNorm 1mg tablets (Novo Nordisk Ltd)	6010203	Feb-09	Meglitinide
11366	NovoNorm 2mg tablets (Novo Nordisk Ltd)	6010203	Feb-09	Meglitinide
11483	Nateglinide 60mg tablets	6010203	Feb-09	Meglitinide

15955	Starlix 120mg tablets (Novartis Pharmaceuticals UK Ltd)	6010203	Feb-09	Meglitinide
23945	Starlix 60mg tablets (Novartis Pharmaceuticals UK Ltd)	6010203	Feb-09	Meglitinide
27125	Starlix 180mg tablets (Novartis Pharmaceuticals UK Ltd)	6010203	Feb-09	Meglitinide
35561	Prandin 2mg tablets (Novo Nordisk Ltd)	6010203	Feb-09	Meglitinide
36774	Prandin 1mg tablets (Novo Nordisk Ltd)	6010203	Feb-09	Meglitinide
36948	Prandin 0.5mg tablets (Novo Nordisk Ltd)	6010203	Feb-09	Meglitinide
52203	Enyglid 0.5mg tablets (Consilient Health Ltd)	6010203	Jan-13	Meglitinide
23	Metformin 500mg tablets	6010202	Feb-09	Metformin
93	Metformin 850mg tablets	6010202	Feb-09	Metformin
735	Metformin 100mg/ml Oral solution	6010202	Feb-09	Metformin
6855	Avandamet 2mg/500mg tablets (GlaxoSmithKline UK Ltd)	0	Feb-09	Metformin
7048	Metformin 500mg modified-release tablets	6010202	Feb-09	Metformin
7166	Glucophage 500mg tablets (Merck Serono Ltd)	6010202	Feb-09	Metformin

7325	Avandamet 4mg/1000mg tablets (GlaxoSmithKline UK Ltd)	0	Feb-09	Metformin
7375	Rosiglitazone 4mg / Metformin 1g tablets	0	Feb-09	Metformin
7610	Glucophage 850mg tablets (Merck Serono Ltd)	6010202	Feb-09	Metformin
11601	Rosiglitazone 2mg / Metformin 500mg tablets	0	Feb-09	Metformin
11604	Rosiglitazone 1mg / Metformin 500mg tablets	0	Feb-09	Metformin
11609	Metformin with rosiglitazone 500mg + 1mg Tablet	06010202/06010203	Feb-09	Metformin
11610	Metformin with rosiglitazone 500mg + 2mg Tablet	06010202/06010203	Feb-09	Metformin
11717	Rosiglitazone 2mg / Metformin 1g tablets	0	Feb-09	Metformin
11737	Metformin with rosiglitazone 1000mg + 4mg Tablet	06010202/06010203	Feb-09	Metformin
11760	Metformin with rosiglitazone 1000mg + 2mg Tablet	06010202/06010203	Feb-09	Metformin
11990	Metformin 500mg/5ml oral solution sugar free	6010202	Feb-09	Metformin
14164	Avandamet 2mg/1000mg tablets (GlaxoSmithKline UK Ltd)	0	Feb-09	Metformin

16044	Glucophage SR 500mg tablets (Merck Serono Ltd)	6010202	Feb-09	Metformin
17580	Avandamet 1mg/500mg tablets (GlaxoSmithKline UK Ltd)	0	Feb-09	Metformin
18220	Pioglitazone 15mg / Metformin 850mg tablets	6010203	Feb-09	Metformin
25678	Glucamet 500mg Tablet (Opus Pharmaceuticals Ltd)	6010202	Feb-09	Metformin
26258	Glucamet 850mg Tablet (Opus Pharmaceuticals Ltd)	6010202	Feb-09	Metformin
27501	Orabet 500mg Tablet (Lagap)	6010202	Feb-09	Metformin
30316	Metformin with pioglitazone 850mg + 15mg Tablet	06010202/06010203	Feb-09	Metformin
31077	Competact 15mg/850mg tablets (Takeda UK Ltd)	6010203	Feb-09	Metformin
31146	Metsol 500mg/5ml oral solution (Kappin Ltd)	6010202	Feb-09	Metformin
33087	Metformin 500mg tablets (Actavis UK Ltd)	6010202	Feb-09	Metformin
33674	Metformin 850mg tablets (A A H Pharmaceuticals Ltd)	6010202	Feb-09	Metformin
34004	Metformin 500mg tablets (IVAX Pharmaceuticals UK Ltd)	6010202	Feb-09	Metformin
34020	Metformin 850mg tablets (IVAX Pharmaceuticals UK Ltd)	6010202	Feb-09	Metformin

34135	Metformin 500mg Tablet (M & A Pharmachem Ltd)	6010202	Feb-09	Metformin
34323	Metformin 500mg tablets (A A H Pharmaceuticals Ltd)	6010202	Feb-09	Metformin
34504	Metformin 500mg tablets (Wockhardt UK Ltd)	6010202	Feb-09	Metformin
34598	Metformin 500mg tablets (Generics (UK) Ltd)	6010202	Feb-09	Metformin
34697	Metformin 850mg tablets (Wockhardt UK Ltd)	6010202	Feb-09	Metformin
34742	Metformin 850mg tablets (Teva UK Ltd)	6010202	Feb-09	Metformin
34836	Metformin 850mg tablets (Actavis UK Ltd)	6010202	Feb-09	Metformin
34917	Metformin 500mg tablets (Teva UK Ltd)	6010202	Feb-09	Metformin
37874	Vildagliptin 50mg / Metformin 850mg tablets	6010203	Feb-09	Metformin
37902	Vildagliptin 50mg / Metformin 1g tablets	6010203	Feb-09	Metformin
38355	Metformin 750mg modified-release tablets	6010202	Feb-09	Metformin
38400	Glucophage SR 750mg tablets (Merck Serono Ltd)	6010202	Feb-09	Metformin
38551	Eucreas 50mg/1000mg tablets (Novartis Pharmaceuticals UK Ltd)	6010203	Feb-09	Metformin
39203	Eucreas 50mg/850mg tablets (Novartis Pharmaceuticals UK Ltd)	6010203	Apr-09	Metformin

39560	Bolamyn SR 500mg tablets (Teva UK Ltd)	6010202	May-09	Metformin
39598	Metformin 1g modified-release tablets	6010202	May-09	Metformin
39729	Glucophage SR 1000mg tablets (Merck Serono Ltd)	6010202	Jun-09	Metformin
39988	Metformin 500mg oral powder sachets sugar free	6010202	Aug-09	Metformin
40007	Glucophage 1000mg oral powder sachets (Merck Serono Ltd)	6010202	Aug-09	Metformin
40110	Glucophage 500mg oral powder sachets (Merck Serono Ltd)	6010202	Aug-09	Metformin
40233	Metformin 1g oral powder sachets sugar free	6010202	Aug-09	Metformin
42161	Orabet 500mg Tablet (Sandoz Ltd)	6010202	Apr-10	Metformin
43270	Metformin 500mg/5ml oral solution sugar free (Rosemont Pharmaceuticals Ltd)	6010202	Aug-10	Metformin
43619	Metformin 1g / Sitagliptin 50mg tablets	6010203	Oct-10	Metformin
43684	Janumet 50mg/1000mg tablets (Merck Sharp & Dohme Ltd)	6010203	Oct-10	Metformin
44250	Metformin 500mg/5ml Oral solution (Hillcross Pharmaceuticals Ltd)	6010202	Jan-11	Metformin

45581	Metabet SR 500mg tablets (Morningside Healthcare Ltd)	6010202	Jun-11	Metformin
46989	Metabet SR 1000mg tablets (Morningside Healthcare Ltd)	6010202	Mar-12	Metformin
47939	Glucient SR 500mg tablets (Consilient Health Ltd)	6010202	Oct-12	Metformin
48149	Metformin 500mg tablets (Almus Pharmaceuticals Ltd)	6010202	Dec-12	Metformin
49502	Glucophage SR 500mg tablets (Mawdsley-Brooks & Company Ltd)	6010202	Jan-13	Metformin
49738	Metformin 1g modified-release tablets (A A H Pharmaceuticals Ltd)	6010202	Jan-13	Metformin
50570	Glucophage SR 500mg tablets (Lexon (UK) Ltd)	6010202	Jan-13	Metformin
50682	Jentadueto 2.5mg/1000mg tablets (Boehringer Ingelheim Ltd)	06010202/06010203	Jan-13	Metformin
50821	Metformin 850mg tablets (Pfizer Ltd)	6010202	Jan-13	Metformin
50970	Metformin 500mg tablets (Bristol Laboratories Ltd)	6010202	Jan-13	Metformin
51080	Metabet SR 1000mg tablets (Actavis UK Ltd)	6010202	Jan-13	Metformin
51135	Metformin 500mg modified-release tablets (A A H Pharmaceuticals Ltd)	6010202	Jan-13	Metformin

51527	Metformin 500mg tablets (Boston Healthcare Ltd)	6010202	Jan-13	Metformin
52221	Diagemet XL 500mg tablets (Thornton & Ross Ltd)	6010202	Jan-13	Metformin
52442	Metformin 500mg tablets (Pfizer Ltd)	6010202	Jan-13	Metformin
52634	Glucophage SR 500mg tablets (DE Pharmaceuticals)	6010202	Jan-13	Metformin
53478	Metformin 500mg modified-release tablets (Kent Pharmaceuticals Ltd)	6010202	Jan-13	Metformin
53774	Metabet SR 500mg tablets (Actavis UK Ltd)	6010202	Feb-13	Metformin
53867	Metformin 500mg tablets (Zentiva)	6010202	Feb-13	Metformin
54150	Jentadueto 2.5mg/850mg tablets (Boehringer Ingelheim Ltd)	06010202/06010203	Mar-13	Metformin
54442	Metformin (roi) 1000mg Tablet	6010202	Apr-13	Metformin
54891	Saxagliptin 2.5mg / Metformin 1g tablets	6010203	May-13	Metformin
54898	Metformin 850mg tablets (Almus Pharmaceuticals Ltd)	6010202	May-13	Metformin
54973	Saxagliptin 2.5mg / Metformin 850mg tablets	6010203	May-13	Metformin
55270	Duformin 500mg Tablet (Dumex Ltd)	6010202	May-13	Metformin

55711	Metformin 500mg tablets (Alliance Healthcare (Distribution) Ltd)	6010202	Jun-13	Metformin
55739	Metformin 500mg tablets (Tillomed Laboratories Ltd)	6010202	Jun-13	Metformin
56965	Komboglyze 2.5mg/1000mg tablets (AstraZeneca UK Ltd)	6010203	Sep-13	Metformin
57147	Bolamyn SR 1000mg tablets (Teva UK Ltd)	6010202	Sep-13	Metformin
57457	Metformin 500mg tablets (Aurobindo Pharma Ltd)	6010202	Oct-13	Metformin
58051	Metformin 500mg/5ml oral solution	6010202	Dec-13	Metformin
58607	Metformin 500mg/5ml oral solution sugar free (Zentiva)	6010202	Jan-14	Metformin
58865	Komboglyze 2.5mg/850mg tablets (AstraZeneca UK Ltd)	6010203	Feb-14	Metformin
59385	Vipdomet 12.5mg/1000mg tablets (Takeda UK Ltd)	6010200	Apr-14	Metformin
59620	Glucophage SR 500mg tablets (Waymade Healthcare Plc)	6010202	Apr-14	Metformin
60012	Dapagliflozin 5mg / Metformin 1g tablets	6010202	May-14	Metformin
60074	Metformin 1g modified-release tablets (Waymade Healthcare Plc)	6010202	Jun-14	Metformin
60286	Metformin 500mg/5ml oral suspension	6010202	Jun-14	Metformin

60497	Alogliptin 12.5mg / Metformin 1g tablets	6010200	Jul-14	Metformin
60643	Xigduo 5mg/1000mg tablets (AstraZeneca UK Ltd)	6010202	Jul-14	Metformin
60968	Metformin 500mg modified-release tablets (Actavis UK Ltd)	6010202	Aug-14	Metformin
61043	Sukkarto SR 1000mg tablets (Morningside Healthcare Ltd)	6010202	Sep-14	Metformin
61559	Sukkarto SR 500mg tablets (Morningside Healthcare Ltd)	6010202	Nov-14	Metformin
32	Gliclazide 80mg tablets	6010201	Feb-09	Sulphonylurea
547	Glipizide 2.5mg tablets	0	Feb-09	Sulphonylurea
1253	Chlorpropamide 100mg tablets	6010201	Feb-09	Sulphonylurea
1254	Glibenclamide 5mg tablets	6010201	Feb-09	Sulphonylurea
1847	Chlorpropamide 250mg tablets	6010201	Feb-09	Sulphonylurea
1964	Diamicron 80mg tablets (Servier Laboratories Ltd)	6010201	Feb-09	Sulphonylurea
1965	Tolbutamide 500mg tablets	6010201	Feb-09	Sulphonylurea
2219	Glibenclamide 2.5mg tablets	6010201	Feb-09	Sulphonylurea
4426	CHLORPROPAMIDE 500 MG TAB	0	Feb-09	Sulphonylurea

4862	Diabetamide 2.5mg tablets (Ashbourne Pharmaceuticals Ltd)	6010201	Feb-09	Sulphonylurea
5276	Glimepiride 1mg tablets	6010201	Feb-09	Sulphonylurea
5316	Glimepiride 4mg tablets	6010201	Feb-09	Sulphonylurea
5353	Glimepiride 2mg tablets	6010201	Feb-09	Sulphonylurea
5627	Gliclazide 30mg modified-release tablets	6010201	Feb-09	Sulphonylurea
5636	Glipizide 5mg tablets	6010201	Feb-09	Sulphonylurea
6337	Glimepiride 3mg tablets	6010201	Feb-09	Sulphonylurea
7284	Amaryl 2mg tablets (Zentiva)	6010201	Feb-09	Sulphonylurea
7332	Amaryl 1mg tablets (Zentiva)	6010201	Feb-09	Sulphonylurea
7409	Amaryl 3mg tablets (Zentiva)	6010201	Feb-09	Sulphonylurea
7744	Daonil 5mg tablets (Sanofi)	6010201	Feb-09	Sulphonylurea
7912	Semi-Daonil 2.5mg tablets (Sanofi)	6010201	Feb-09	Sulphonylurea
8034	Diabinese 100mg Tablet (Pfizer Ltd)	6010201	Feb-09	Sulphonylurea
8168	Diabinese 250mg Tablet (Pfizer Ltd)	6010201	Feb-09	Sulphonylurea
8390	Gliquidone 30mg tablets	0	Feb-09	Sulphonylurea
8976	Euglucon 2.5mg tablets (Aventis Pharma)	6010201	Feb-09	Sulphonylurea
9108	TOLBUTAMIDE 250 MG TAB	0	Feb-09	Sulphonylurea
10427	Tolazamide 250mg Tablet	6010201	Feb-09	Sulphonylurea

11284	Amaryl 4mg tablets (Zentiva)	6010201	Feb-09	Sulphonylurea
11695	Diamicron 30mg MR tablets (Servier Laboratories Ltd)	6010201	Feb-09	Sulphonylurea
11946	Tolbutamide 50mg/ml Injection	6010201	Feb-09	Sulphonylurea
12245	Glutril 25mg Tablet (Roche Products Ltd)	6010201	Feb-09	Sulphonylurea
12259	Glibornuride 25mg Tablet	6010201	Feb-09	Sulphonylurea
12455	Rastinon 500mg Tablet (Hoechst Marion Roussel)	6010201	Feb-09	Sulphonylurea
12513	Glibenese 5mg tablets (Pfizer Ltd)	6010201	Feb-09	Sulphonylurea
13331	Euglucon 5mg tablets (Sanofi)	6010201	Feb-09	Sulphonylurea
15374	Gliclazide 40mg/5ml oral suspension	6010201	Feb-09	Sulphonylurea
16211	TOLBUTAMIDE 100 MG TAB	0	Feb-09	Sulphonylurea
16602	Calabren 2.5mg Tablet (Berk Pharmaceuticals Ltd)	6010201	Feb-09	Sulphonylurea
17343	Gliclazide 80mg tablets (A A H Pharmaceuticals Ltd)	6010201	Feb-09	Sulphonylurea
17698	Minodiab 5mg tablets (Pfizer Ltd)	6010201	Feb-09	Sulphonylurea
17706	Minodiab 2.5mg tablets (Pfizer Ltd)	0	Feb-09	Sulphonylurea
19336	Tolazamide 100mg Tablet	6010201	Feb-09	Sulphonylurea
19658	Glurenorm 30mg tablets (Sanofi)	0	Feb-09	Sulphonylurea

19728	GONDAFON .5 GM TAB	0	Feb-09	Sulphonylurea
21424	Glibenclamide 5mg/5ml oral suspension	6010201	Feb-09	Sulphonylurea
21489	Tolanase 250mg Tablet (Pharmacia Ltd)	6010201	Feb-09	Sulphonylurea
21564	Gliclazide 80mg tablets (Wockhardt UK Ltd)	6010201	Feb-09	Sulphonylurea
21832	Diabetamide 5mg tablets (Ashbourne Pharmaceuticals Ltd)	6010201	Feb-09	Sulphonylurea
21870	RASTINON	0	Feb-09	Sulphonylurea
21892	Diaglyk 80mg tablets (Ashbourne Pharmaceuticals Ltd)	6010201	Feb-09	Sulphonylurea
22145	Tolanase 100mg Tablet (Pharmacia Ltd)	6010201	Feb-09	Sulphonylurea
22614	DAONIL 10 MG TAB	0	Feb-09	Sulphonylurea
22636	TOLBUTAMIDE 1 GM TAB	0	Feb-09	Sulphonylurea
22858	Acetohexamide 500mg tablets	6010201	Feb-09	Sulphonylurea
24848	Glymidine sodium 500mg Tablet	6010201	Feb-09	Sulphonylurea
25636	Libanil 2.5mg Tablet (Approved Prescription Services Ltd)	6010201	Feb-09	Sulphonylurea
26118	Dimelor 500mg Tablet (Eli Lilly and Company Ltd)	6010201	Feb-09	Sulphonylurea
26218	Calabren 5mg Tablet (Berk Pharmaceuticals Ltd)	6010201	Feb-09	Sulphonylurea

27969	Glymese 250mg Tablet (DDSA Pharmaceuticals Ltd)	6010201	Feb-09	Sulphonylurea
28708	Malix 2.5mg Tablet (Lagap)	6010201	Feb-09	Sulphonylurea
29326	Glipizide 5mg tablets (Generics (UK) Ltd)	6010201	Feb-09	Sulphonylurea
29939	Gliclazide 80mg tablets (Generics (UK) Ltd)	6010201	Feb-09	Sulphonylurea
30460	Malix 5mg Tablet (Lagap)	6010201	Feb-09	Sulphonylurea
31212	Gliclazide 80mg tablets (Actavis UK Ltd)	6010201	Feb-09	Sulphonylurea
31474	Libanil 5mg Tablet (Approved Prescription Services Ltd)	6010201	Feb-09	Sulphonylurea
33562	Duclazide 80mg Tablet (Dumex Ltd)	6010201	Feb-09	Sulphonylurea
33673	Tolbutamide 500mg tablets (Actavis UK Ltd)	6010201	Feb-09	Sulphonylurea
34399	Gliclazide 80mg tablets (IVAX Pharmaceuticals UK Ltd)	6010201	Feb-09	Sulphonylurea
34507	Glibenclamide 2.5mg tablets (Wockhardt UK Ltd)	6010201	Feb-09	Sulphonylurea
34563	Glibenclamide 5mg tablets (Wockhardt UK Ltd)	6010201	Feb-09	Sulphonylurea
34676	Glibenclamide 2.5mg tablets (A A H Pharmaceuticals Ltd)	6010201	Feb-09	Sulphonylurea
34706	Glibenclamide 2.5mg tablets (IVAX Pharmaceuticals UK Ltd)	6010201	Feb-09	Sulphonylurea

34802	Glipizide 5mg tablets (IVAX Pharmaceuticals UK Ltd)	6010201	Feb-09	Sulphonylurea
34932	Gliclazide 80mg tablets (Genus Pharmaceuticals Ltd)	6010201	Feb-09	Sulphonylurea
34957	Tolbutamide 500mg tablets (A A H Pharmaceuticals Ltd)	6010201	Feb-09	Sulphonylurea
36856	Gliclazide 80mg tablets (Sandoz Ltd)	6010201	Feb-09	Sulphonylurea
40365	Glimepiride 1mg tablets (Actavis UK Ltd)	6010201	Sep-09	Sulphonylurea
40425	Nazdol MR 30mg tablets (Teva UK Ltd)	6010201	Sep-09	Sulphonylurea
41558	Glibenclamide 5mg tablets (Teva UK Ltd)	6010201	Mar-10	Sulphonylurea
41559	Glibenclamide 5mg tablets (A A H Pharmaceuticals Ltd)	6010201	Mar-10	Sulphonylurea
41593	Glibenclamide 2.5mg tablets (Teva UK Ltd)	6010201	Mar-10	Sulphonylurea
41898	GLIBENCLAMIDE	0	Apr-10	Sulphonylurea
42790	Gliclazide 80mg Tablet (Merck Generics (UK) Ltd)	6010201	Jul-10	Sulphonylurea
43065	Gliclazide 40mg tablets	6010201	Aug-10	Sulphonylurea
43465	Zicron 40mg tablets (Bristol Laboratories Ltd)	6010201	Sep-10	Sulphonylurea
44304	Glyconon 500mg Tablet (DDSA Pharmaceuticals Ltd)	6010201	Jan-11	Sulphonylurea
44473	Edicil MR 30mg tablets (Ratiopharm UK Ltd)	6010201	Jan-11	Sulphonylurea

44738	Niddaryl 1mg tablets (Dee Pharmaceuticals Ltd)	6010201	Mar-11	Sulphonylurea
45215	Gliclazide 80mg Tablet (Neo Laboratories Ltd)	6010201	May-11	Sulphonylurea
45831	Dacadis MR 30mg tablets (Generics (UK) Ltd)	6010201	Aug-11	Sulphonylurea
46927	Tolbutamide 500mg tablets (Teva UK Ltd)	6010201	Mar-12	Sulphonylurea
47074	Gliclazide 80mg/5ml oral suspension	6010201	Apr-12	Sulphonylurea
47894	Nazdol MR 30mg tablets (Consilient Health Ltd)	6010201	Oct-12	Sulphonylurea
48056	Gliclazide 80mg tablets (Sovereign Medical Ltd)	6010201	Nov-12	Sulphonylurea
51955	Gliclazide 80mg tablets (Accord Healthcare Ltd)	6010201	Jan-13	Sulphonylurea
53288	Gliclazide 30mg modified-release tablets (A A H Pharmaceuticals Ltd)	6010201	Jan-13	Sulphonylurea
54764	Gliclazide 80mg tablets (Arrow Generics Ltd)	6010201	Apr-13	Sulphonylurea
55862	Gliclazide Oral solution	6010201	Aug-13	Sulphonylurea
56008	Gliclazide 80mg tablets (Almus Pharmaceuticals Ltd)	6010201	Aug-13	Sulphonylurea
56376	Rosiglitazone 4mg with glimepiride 4mg tablet	06010201/06010203	Aug-13	Sulphonylurea
56437	Gliclazide 60mg modified-release tablets	6010201	Aug-13	Sulphonylurea
57601	Daonil 5mg tablets (Dowelhurst Ltd)	6010201	Oct-13	Sulphonylurea

57830	Gliclazide 30mg modified-release tablets (Alliance Healthcare (Distribution) Ltd)	6010201	Dec-13	Sulphonylurea
58882	Gliclazide 120mg/5ml oral suspension	6010201	Feb-14	Sulphonylurea
60495	Gliclazide 80mg tablets (Teva UK Ltd)	6010201	Jul-14	Sulphonylurea
61311	Glimepiride 4mg tablets (Sigma Pharmaceuticals Plc)	6010201	Oct-14	Sulphonylurea
469	Rosiglitazone 4mg tablets	0	Feb-09	Thiazolidinedione
548	Pioglitazone 15mg tablets	6010203	Feb-09	Thiazolidinedione
5227	Rosiglitazone 8mg tablets	0	Feb-09	Thiazolidinedione
6855	Avandamet 2mg/500mg tablets (GlaxoSmithKline UK Ltd)	0	Feb-09	Thiazolidinedione
7325	Avandamet 4mg/1000mg tablets (GlaxoSmithKline UK Ltd)	0	Feb-09	Thiazolidinedione
7375	Rosiglitazone 4mg / Metformin 1g tablets	0	Feb-09	Thiazolidinedione
9662	Avandia 4mg tablets (GlaxoSmithKline UK Ltd)	0	Feb-09	Thiazolidinedione
9699	Pioglitazone 30mg tablets	6010203	Feb-09	Thiazolidinedione
10051	Pioglitazone 45mg tablets	6010203	Feb-09	Thiazolidinedione
11601	Rosiglitazone 2mg / Metformin 500mg tablets	0	Feb-09	Thiazolidinedione
11604	Rosiglitazone 1mg / Metformin 500mg tablets	0	Feb-09	Thiazolidinedione

11609	Metformin with rosiglitazone 500mg + 1mg Tablet	06010202/06010203	Feb-09	Thiazolidinedione
11610	Metformin with rosiglitazone 500mg + 2mg Tablet	06010202/06010203	Feb-09	Thiazolidinedione
11717	Rosiglitazone 2mg / Metformin 1g tablets	0	Feb-09	Thiazolidinedione
11737	Metformin with rosiglitazone 1000mg + 4mg Tablet	06010202/06010203	Feb-09	Thiazolidinedione
11760	Metformin with rosiglitazone 1000mg + 2mg Tablet	06010202/06010203	Feb-09	Thiazolidinedione
13628	Romozin 400mg Tablet (Glaxo Wellcome UK Ltd)	6010203	Feb-09	Thiazolidinedione
14164	Avandamet 2mg/1000mg tablets (GlaxoSmithKline UK Ltd)	0	Feb-09	Thiazolidinedione
15232	Avandia 8mg tablets (GlaxoSmithKline UK Ltd)	0	Feb-09	Thiazolidinedione
17580	Avandamet 1mg/500mg tablets (GlaxoSmithKline UK Ltd)	0	Feb-09	Thiazolidinedione
18220	Pioglitazone 15mg / Metformin 850mg tablets	6010203	Feb-09	Thiazolidinedione
19472	Actos 45mg tablets (Takeda UK Ltd)	6010203	Feb-09	Thiazolidinedione
20287	Actos 15mg tablets (Takeda UK Ltd)	6010203	Feb-09	Thiazolidinedione

20889	Actos 30mg tablets (Takeda UK Ltd)	6010203	Feb-09	Thiazolidinedione
30316	Metformin with pioglitazone 850mg + 15mg Tablet	06010202/06010203	Feb-09	Thiazolidinedione
31077	Competact 15mg/850mg tablets (Takeda UK Ltd)	6010203	Feb-09	Thiazolidinedione
37617	Rosiglitazone 2mg tablet	6010203	Feb-09	Thiazolidinedione
48120	Avandia 2mg Tablet (GlaxoSmithKline UK Ltd)	6010203	Dec-12	Thiazolidinedione
48139	Pioglitazone 30mg tablets (A A H Pharmaceuticals Ltd)	6010203	Dec-12	Thiazolidinedione
56208	Pioglitazone 15mg tablets (A A H Pharmaceuticals Ltd)	6010203	Aug-13	Thiazolidinedione
56376	Rosiglitazone 4mg with glimepiride 4mg tablet	06010201/06010203	Aug-13	Thiazolidinedione
56831	Troglitazone 200mg Tablet	6010203	Aug-13	Thiazolidinedione
57659	Pioglitazone 30mg tablets (Actavis UK Ltd)	6010203	Oct-13	Thiazolidinedione
